# Cohort profile: Bandar Kong prospective study of chronic non-communicable diseases

**DOI:** 10.1371/journal.pone.0265388

**Published:** 2022-05-12

**Authors:** Azim Nejatizadeh, Ebrahim Eftekhar, Mohammad Shekari, Hossein Farshidi, Seyed Hossein Davoodi, Mehdi Shahmoradi, Hossein Poustchi, Amin Ghanbarnejad, Teymour Aghamolaei, Hadi Yousefi, Shideh Rafati

**Affiliations:** 1 Molecular Medicine Research Center, Hormozgan University of Medical Sciences, Bandar Abbas, Iran; 2 Cardiovascular Research Center, Hormozgan University of Medical Sciences, Bandar Abbas, Iran; 3 Cancer Research Center, Shahid Beheshti Medical University, Tehran, Iran; 4 Liver and Pancreatobiliary Diseases Research Center, Digestive Diseases Research Institute, Tehran University of Medical Sciences, Tehran, Iran; 5 Social Determinants in Health Promotion Research Center, Faculty of Health, Hormozgan University of Medical Sciences, Bandar Abbas, Iran; 6 Department of Nursing, Faculty of Nursing and Midwifery, Hormozgan University of Medical Sciences, Bandar Abbas, Iran; All India Institute of Medical Sciences - Bhopal, INDIA

## Abstract

Chronic non-communicable diseases (NCDs), are the leading causes of death among adults worldwide. It is projected that half of the NCDs could be avoided by preventing measures. Under the prospective epidemiological research studies in Iran (PERSIAN), we established a prospective population-based cohort study in southern Iran. The present study was designed to observe changing pattern of lifestyle transition over time and investigate the incidence and prevalence of regional modifiable risk factors as well as their associations with major NCDs. At baseline, 4063 participants aged 35–70 years were recruited on Oct, 2016and planned to get re-evaluated every 5 years along with annual follow-up. Data using validated electronic questionnaire comprising 55 questions and 482 items including general, medical and nutrition queries was collected. Blood, hair, nails, urine specimens and anthropometric measurements were taken. The response rate was 99%. In the results; male and female participants were 42.5% and 57.5%, respectively. Of note, 30.4% of women and 16.1% of men were obese. The prevalence of hypertension in men and women was 14.6% and 21%; however, diabetic men and women were 17.4% and 12.4%, respectively. Living in rural areas increased the odds of having hypertension by 1.33 (AOR = 1.33, 95% CI:1–09, 1.61). Noteworthy, logistic regression displayed that aging could predispose individuals to be more overweight, hypertensive and diabetic. The prevalence of multimorbidity of 3 or more NCDs were 8% (No. 326) and 6% (No.240), respectively. Intake of fruits, vegetables and dairy was less than two servings per day in 9.2%, 13% and 58.3% of the participants. Lower cardiovascular diseases and serum level of FBS and higher HDL level in sailors/fishermen compared to other job groups were significant (p-value <0.001). The second annual follow-up was completed and now at the end of the third wave. Findings of the present study signified the high prevalence of behavioral risk factors and their associations with respective NCDs. Subsequently, it is essential to keep track lifestyle variations, the modifiable risk factors and NCDs trends by prospective population-based cohort studies.

## Introduction

Non-communicable diseases (NCDs), known as chronic diseases, resulting from a complex interaction of genetic, behavioral and environmental factors, being defined as diseases of long duration and slow development that have led to experience high morbidity and mortality, particularly, in low and middle income countries, account for almost more than 70% of all burden of diseases worldwide. The main force drivers to NCDs are rapid urbanization, industrialization, globalization, aging and mostly originate from low physical activity, morbid diet, smoking and harmful use of alcohol consumption leading to raised blood lipids and glucose, hypertension and excess body weight. Although, NCDs affect all age groups, adults and elderly often experience the common chronic diseases, with nearly equal distribution among men and women [1, 2]. According to WHO report, NCDs are responsible for 76.4% of all deaths so as cardiovascular diseases, cancers, respiratory disorders and diabetes mellitus, each account for 45.7%, 13.5%, 3.8% 2.2% of death, respectively. With respect to the non-modifiable risk factors including aging, gender, family history and ethnicity, the major culprit of morbidity and mortality being attributed to modifiable behavioral risk factors [3]. The "premature" death due to NCDs largely happen, between ages 30 to 69 years, in low- and middle-income countries [3]. Early detection, screening and timely management, are vital components to prevent and control NCDs [3, 4]. It is expected that more than half of NCDs burden could be evaded by averting the major modifiable risk factors. The World Health Organization’s Eastern Mediterranean region (EMR), has now declared that in Iran, NCDs is increasing and has got a critical condition leading to annual 2.2 million deaths and growing over time, emerging as a hot-spot of NCDs [5].

In the middle east, Iran with 1% of world’s population, has also encountered unhealthy behaviors and subsequently growing NCDs where overweight, obesity and cardiovascular diseases are on the rise [6]. The result of NCDs surveillance in Iran revealed that overweight/obesity, and hypertension as the main risk factors for NCDs, are the same as the world. Approximately 800 to 850 deaths daily happen, 89% of which are mainly due to Cardiovascular (46%), cancer (14%), respiratory (6%), perinatal diseases (6%) and accidents (17%) [7, 8]. The NCDs burden and mortality rate have been distributed unevenly at national level; therefore, mapping of regional NCDs according to climate and geographic areas is of importance. The latest study in southern Iran which was conducted in Hormozgan province revealed that 25% of the adult population was at risk of NCDs. This investigation also showed higher BMI (24.3%) in women, 17.2% with hypertension, 14% with consumption of less than 5 servings of fruits and vegetables (lower than national level), 28.6% with physical inactivity (higher than national level) and at least 25% of the subjects simultaneously carried 3 main risk factors contributing to NCDs [9]. Another study in the same province showed that the rate of cigarette smoking and Hookah was above 15% around 13%, respectively, which were significantly correlated with age and education level [10]. It is well recognized that adequate region-specific evidence to NCDs is required in order to prevent and control the modifiable risk factors and then manage the disease. Undoubtedly, implementation of prospective population-based cohort studies dedicated to local cultures and ethnicities could generate region-specific evidence to evaluate a complex network of health determinants in order to design and administer unique interventions tackling NCDs [11–13]. The north of Hormozgan province is mountainous, however, to the south, it is limited to the coastal plains. Environmental exposures and life style of the coastal residents are quite different compared to mountainous areas that itself could likely lead to the development of different region-specific risk factors [14, 15]. At a glance, some measured biochemical variables in PERSIAN cohort study were presented [16–20] (Table 1). Bandar Kong cohort study as a part of PERSIAN cohort study (http://persiancohort.com), being the first study in terms of size/design and perspectives to investigate the health profile of the inhabitants.

**Table 1:** Biochemical variables in various studies of PERSIAN cohort study.

Cohort	Hormozgan cohort	Fasa PERSIAN cohort (12)	Rafsanjan Cohort (13)	Great Khorasan Cohort (14)	PERSIAN Guilan cohort (15)	Azar cohort (16)
Variables						
**Sample size**	4040	9975	9990	2233	950	13099
**FBS (**mg/dL**)**	108.13±42.968	92.375±28.615	113.27±39.11	88.28±25.22	102.59±31.7	98.34±32.40
**SGOT (AST) (**U/L**)**	21.95±11.129	22.7±8.655	19.87±11.81	-	20.08±9.8	-
**SGPT (ALT) (**U/L**)**	27.17±21.4	25.19±14.27	21.55±15.37	-	21.68±16.4	-
**Triglycerides (TG) (**mg/dL**)**	136.59±87.547	132.24±82.81	168.88±109.22	132.74±97.34	172.83±94.5	149.12±84.25
**GGT (U/L)**	28.946±32.302	23.12±21.135	-	-	27.69±23.3	-
**HDL-C (**mg/dL**)**	47.952±10.7788	50.73±15.42	57.90±12.45	33.20±13.51	45.24±9.4	45.42±10.09
**LDL-C (**mg/dL**)**	127.56±34.574	107.505±32.435	108.04±30.50	123.55±38.61	121.51±30.9	-
**Total Cholesterol (TC) (**mg/dL**)**	202.39±42.375	184.605±38.67	198.78±41.89	189.18±46.33	201.43±37±0.2	-

We aimed to determine the impact of environmental differences and exposures at coastlines on inhabitants’ lifestyle and to estimate the prevalence of region-specific modifiable risk factors and their associations underlying NCDs with particular focus on sailors/fishermen, in southern Iran. Moreover, we launched and adopted a large biobank storing various human specimens under standard protocols for deciphering the role of molecular/ genomic biomarkers and environmental factors and their interactions in developing NCDs.

## Materials and methods

### Ethics and informed consent

The study was approved by the Ethical Review Board of Hormozgan University of Medical Sciences. Written informed consent was obtained from each participant, upon fulfillment of inclusion/exclusion criteria and willingness to participate.

### Geographic location of the cohort

**Fig 1:**
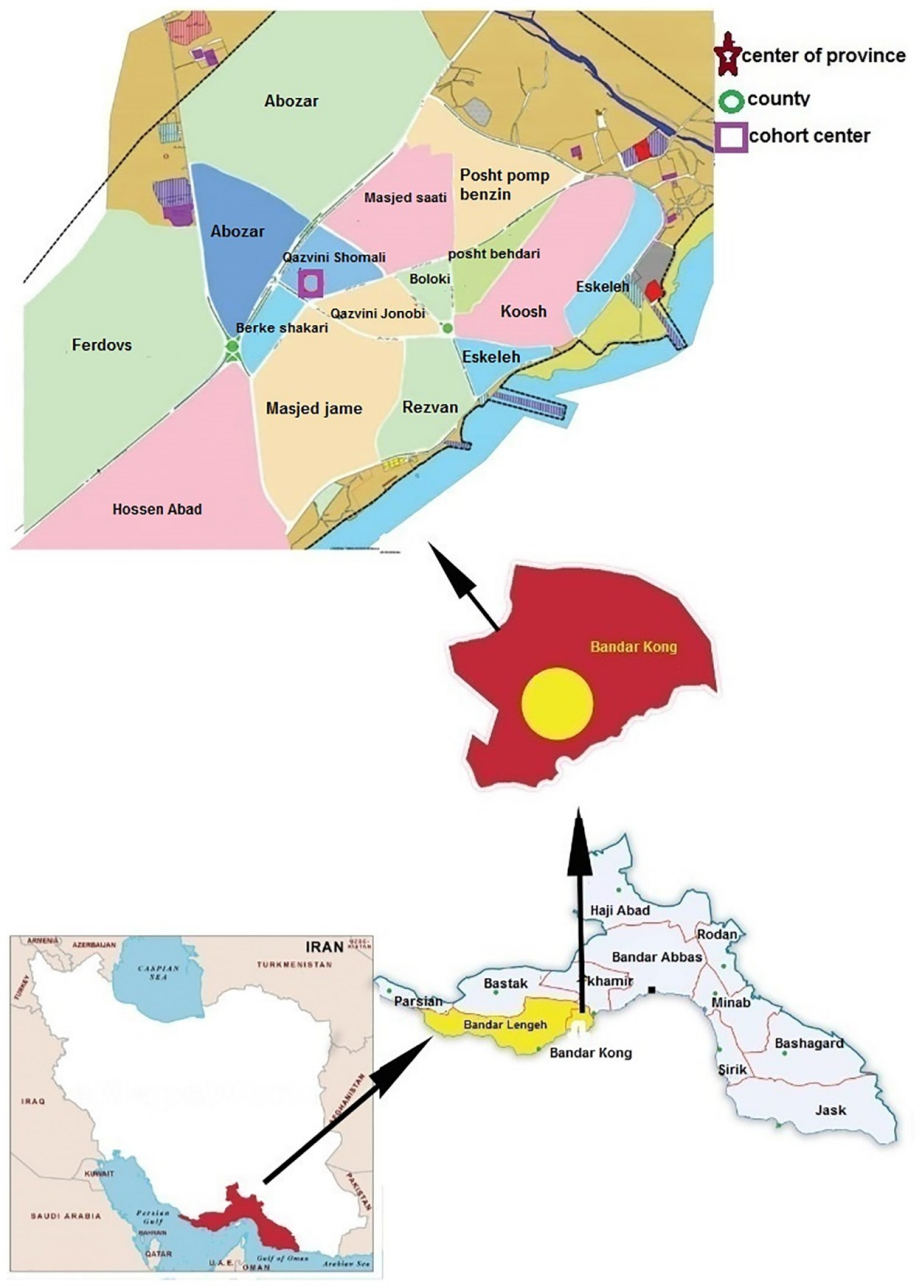
The map of Iran, Hormozgan province and geographic locations of Bandar Kong cohort study in southern Iran. Upper part of the figure approximately corresponds to the street map of cohort study field. [Republished from 1. Organization of Management and Planning of Hormozgan Province; https://www.ncc.gov.ir/images/docs/files; https://www.amar.org.ir/, under a CC BY license, with permission from the Mayor of the Bandar Kong and above-mentioned body, original copyright [2006]; 2. The image was also visualized by Google Maps (https://www.google.cat/maps/)].

Hormozgan, Iran’s wonderland, the southernmost coastline province, is one of the hot and humid regions of Iran, have got desert and semi desert climate. The province, facing Oman and UAE, has an area of 70697 square kilometers along with 1000 km of coastline [21, 22]. There is scanty precipitation year-round. According to the latest census in 2016, it possesses more than 1.7 million people, consisting 53% men and 47% women, of which, 54.7% are urban and 46.3% rural. The main food is made up of various aquatic animals. A big number of industries and infrastructures are located in the Province. Shahid Rajaee Port, Bandar Abbas refinery, Bandar Abbas power plant, Ship building industry, and a number of dams can be listed as a few. Fishing and 30% of Iran’s fishery produce, hand-built of Lenj vessels, sea transport and agriculture are the other activities of the coastal areas [23]. It has 8 coastal counties. Our Study was established in Bandar Kong county, a harbor city, in the southernmost point of Iran, standing on the height of 5 meters above sea level with hot summers and mild winters (Fig 1). The county is an ancient port with a history of ancient trade which has been registered in the UNESCO. Bandar Kong, placed in the west of Hormozgan province, is known as a historical port where was a dynamic port during Achaemenes Empire. The occupation of most people is related to the sea and sailing [24].

### Study design

The study was designed based on PERSIAN Cohort study (S3 File). Our study was launched in October 2016 to undertake baseline survey and annual follow-ups on the health status of the inhabitants of hormozgan province in southern coastlines of the country, at least for a period of 15years. It was planned to get repeated every 5 years. The cohort center was comprised well-designed rooms for reception, enrollment, sample taking, measurements and interview coupled with an equipped laboratory with a standard biobank. All the instruments and consumable materials were procured and set up by qualified experts according to PERSIAN Cohort Protocol [25, 26].

### Study population

At first, the scientific committee enrolled 4200 participants out of 6000 permanent residents aged 35–70 years in accordance with PERSIAN Cohort arrangement. Sample collection was carried out based on the respective health center statistics and urban/rural divisions. The required sample size was calculated by Fleiss with continuity correction formula, with a two-sided significance level(1-alpha) = 0.95, power (1-beta, % chance of detecting) = 85%, the ratio of sample size of unexposed/exposed = 1, percent of unexposed with outcome = 5%, and risk/ prevalence ratio or odds ratio = 1.5. So, it was estimated 2017 people for the exposed group, 2017 non-exposed group, and hence the total required sample size was estimated at 4034 people [27]. The inclusion criteria were: (i) willingness to participation, (ii) aged 35–75, (iii) residency for one year and at least nine months each year, (iv) written informed consent, (v) Iranian national. Exclusion criteria included: (i) no interest to attend, (ii) being guest, and (iii) mental or physical disabilities.

### The cohort staff

Local individuals were recruited and trained in cohort principles and interview techniques and procedures by professional researchers. The cohort staff were comprised a physician, six interviewers, a nurse, an administration support officer, a field manager, an epidemiologist, two nutritionists and a biochemist. The Study being supervised by the principal investigator and run by the executive team along with contribution of a panel of academics.

### Enrollment phase

**Fig 2:**
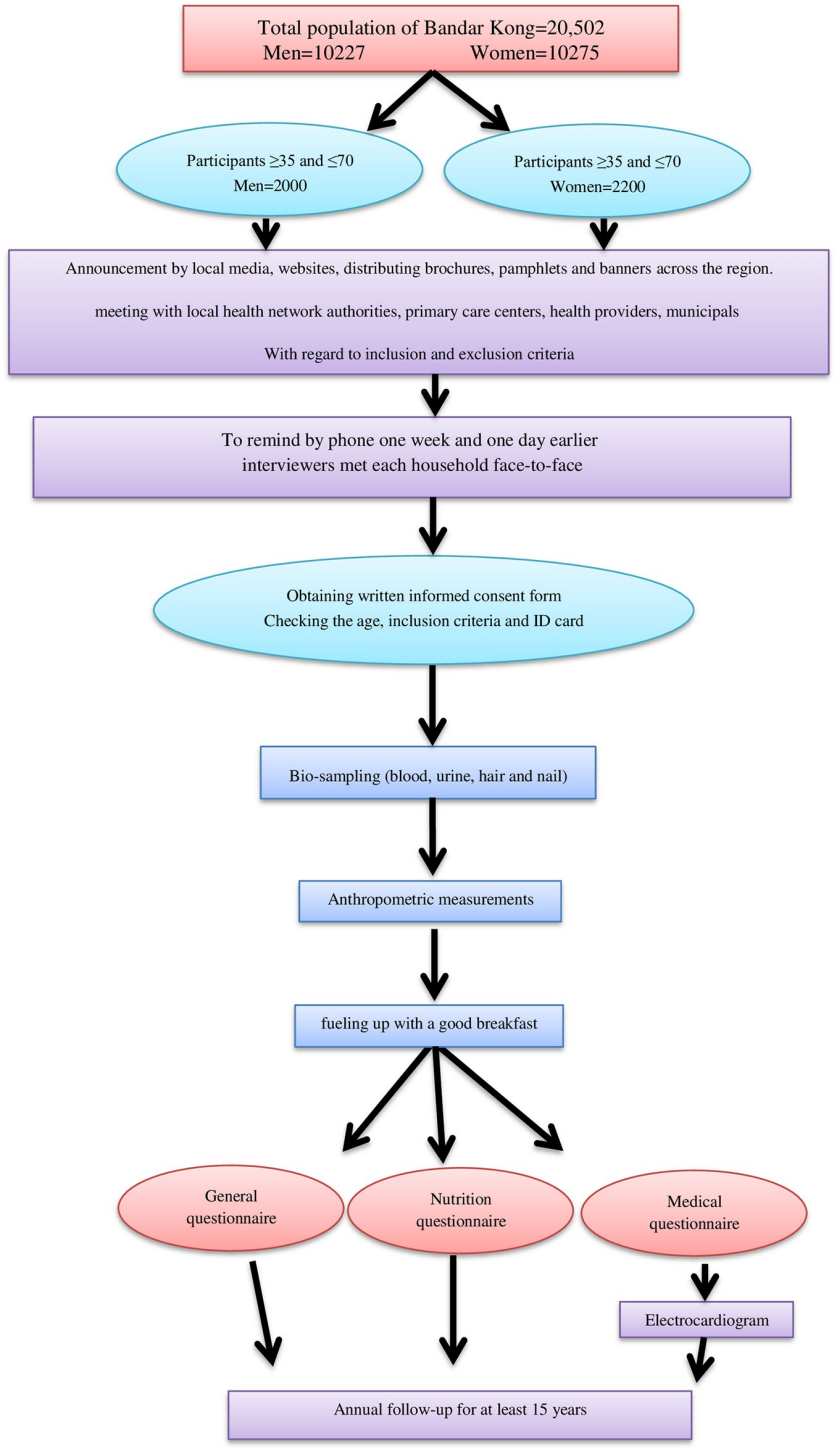
The phases of the data collection process of Kong cohort study.

The main phase of the study started on Oct., 27, 2016. The details were discussed by constructive meetings with local authorities, health care providers, stakeholders, city council, the mayor. We announced the benefits of the study to the public by awareness-raising activities including local media, websites, distributing brochures, pamphlets. Furthermore, a couple of meetings with local health network authorities, were held and technical details and procedures were deliberated. The health-defined block was selected from the nearest one to the primary health center. According to the assigned addresses, the interviewers met each household, explained the study and gave them a pamphlet for further details. After obtaining informed consent, each participant received a dated invitation and were reminded by phone one week and one day earlier. They were requested not to clip their nails 7–10 days before, and to keep clean the hair, as well as being fast at least for 10–12 hours overnight of the attendance day. They were invited in a designated time and location where trained staff initiated field-level data collection. A 11-digit code namely PERSIAN Cohort Identification (PCID) was assigned. Next, 25ml blood sample from each were stored in 1 to 1.5ml 2-D cryotube (Micronic, Lelystad, the Netherlands) at -80 C° and 15 mL of second voiding in the morning was requested. Around 500 hairs, 1–3 cm long and 1-mm nail clippings from all fingers were collected and kept in a dry and cold place. Weight (kg), height (cm), wrist, hip and waist circumferences (cm) were measured. The participants then were entertained with breakfast. The outline of the 4-step enrollment was summarized in the Fig 2.

### Data collection and assessment of exposures

Approximately 99% of the volunteers took part in the cohort study. When the participants attended the cohort center, A detailed electronic questionnaire (S1 File) including 55 questions and 482 items, divided into three key fields, viz., general, medical and nutritional components were administered by four interviewers (Table 2). Individuals who have smoked at least 100 cigarettes for whole life and or smoke every day or some days weekly, were considered current smoker. Regular exercise was considered as any physical activity performed at least 30 minutes for three or more days per week, except walking for work or life. Dietary pattern was measured through a food frequency questionnaire (FFQ) customized for the Iranians. A 132-item semi-quantitative FFQ was filled by trained nutritionists to assess the dietary intakes of the participants over the past year. Information of each food item was asked by two questions: 1) the frequency (number of times) of food intake per day, week, or month in the previous year, and 2) the amount of food (portion size based on the standard serving sizes commonly consumed by Iranians) commonly eaten every time. All dietary intakes were converted to grams per day by using household portion sizes of consumed foods (Table 2).

**Table 2:** Measures and tools administered at the enrollment phase.

Field	Interview	Measures
**Demographic and Socio-economics**	Questionnaire: 82 items	Age, sex, education, residency, employment status, ethnicity, socio-economic status, residential status, marriage status, landline and mobile phone access, fuel status at home, national and abroad journeys,
**Lifestyle**	Questionnaire: 69 items	Sleep quality, drug use, pesticide use, tea consumption, history of smoking, alcohol consumption, daily activity,
**Oral & dental health**	Questionnaire: 9 items	Tooth brushing, dental floss, mouthwash use, DMF status, Implant number/ status
**Physical activity**	Questionnaire: 28 items	Duration and intensity of physical activity, sport activity, daily activity at home and outside, spent hours for media
**Fertility History**	Questionnaire: 33 items	Menstruation, pregnancy, breastfeeding, contraceptive use
**History of chronic disease**	Questionnaire: 81 items	Diabetes mellitus, hypertension, cardiovascular disease, chronic lung disease, rheumatic disease, cancers, psychiatric disorders
**Family history of chronic disease**	Questionnaire: 27 items	history of chronic disease in the family
**Personal habits**	Questionnaire: 4 items	Cigarette and hookah smoking, alcohol and drug use, annual reading,
**Nutrition**	Questionnaire: 168 items	Food Frequency Questionnaire (FFQ), nutritional supplements, drinking water, food habits, preparation
**Physical examination and anthropometric**	Clinical measurements	Height, weight, waist and hip measurements, alopecia, spine disorders, Blood pressure

Following the questionnaire, at first, height was measured in centimeters, standardized to the nearest 0.5 cm, by means of a Height Rod Wall, FAZZINI S208, Italy, 2015. Weight was measured to the nearest 10 g, in kilograms via transportable Weighing scale, RGZ 160, China. The trained staff used SECA 201 retractable tape to measure waist and hip circumferences. Waist-hip ratio and waist-height ratio were calculated. Subsequently, physical examination was fulfilled by a trained physician. Systolic and diastolic blood pressure were measured as per the protocol. A standard mercury sphygmomanometer on the right arm twice so the first one done upon 5- minute rest in sitting position and the last one was taken 15 minutes later based on the protocols, and then the mean values were used for analysis. Pulse rate was also examined. Biochemical and hematological assays including of fasting blood glucose (mg/dl), serum cholesterol, low-density lipoprotein, high-density lipoprotein (HDL), triglycerides, urea, creatinine, uric acid, complete and differential blood count along with erythrocyte sedimentation rate (ESR) were performed. All measurements were according to a standard laboratory protocol using Pars Azmoon kits done by Hematology Cell counter (Mindray, China, 2017) and Biochemistry Auto Analyzer (BT1500, Italy, 2017). The remaining blood specimens were stored at -80C° until analysis in future studies (Table 3). Besides, the collected urine samples were kept in -20C° for macroscopic and microscopic analysis.

**Table 3:** Blood taken from each individual and stored.

Sample type	Aliquots	Storage
Whole blood	Two 1.5 ml Cryo Tubes	−80°^C^
Plasma	Two 1.5 ml, and four 1 ml Cryo Tubes	−80°^C^
Buffy coat	Two 1 ml Cryo Tubes	−80°^C^
Red Blood Cells	One 1 ml Cryo Tube	−80°^C^
Serum	Two 1.5 ml Cryo Tubes	−80°^C^
**Total**	25 ml	

### Annual follow-ups and re-surveys

Fresh and additional detailed data being measured for the follow-up investigations to adjust and improve the data collected at the baseline. Through in-person interview via phone, this phase aimed to look for the same objectives as at enrollment phase besides: a) to evaluate demographic transitions and renew the recorded socio-demographic characteristics and addresses, b) to study lifestyle changes and respective trends, c) to collect detailed information on personal and family medical history, d) to observe and review any recent treatment, hospitalization, risk factors and NCDs or death. In case of any emerging outcome or condition, the participants are requested for further evaluations, otherwise, they will get visited in their residence. Subsequently, three medical consultants autonomously evaluate the clinical findings and assign a diagnosis code to each outcome. Hence, the two disease codes are compared, and if they are unlike, a third consultant will review and finalize the diagnosis. We, now, completed the second annual follow-up and now at the end of the third with participation rate of 99%. We designed to re-survey the whole enrollment phase including 50% of the participants every 5-year for at least 15 years along with annual follow-up.

### Quality assurance (QA) and quality control (QC)

An epidemiologist was designated as in-charge of the quality assurance and quality control. Collected data was checked daily by each interviewer and field controller. A quality control manager observed the data for missing elements, completeness and consistency using a validated checklist on a daily basis. Subsequently, a biostatiscian was committed to regular surveillance of the data quality at the weekends. Furthermore, online data monitoring and interviewers’ performance were observed by central PERSIAN cohort team on a smart data server. Data clean-up being carried out monthly by local and central teams. To control and monitor the quality and accuracy of measuring devices, we calibrated all the concerned devices periodically according to the standard protocols. To prevent information bias, self-administered information was avoided and inconsistent records were corrected. Measurement methods were standardized too. Consistent measurement of inter-assay coefficients of variation was carried out just for normal levels on a regular basis (Table 4).

**Table 4:** Inter-assay coefficients of variation (CV) for biochemistry measurements.

Analyte	Level (normal/High)	CV (normal/High)^1^
**Fasting blood sugar (**mg/dL)	92.2	3.3%
	254	1.7%
**Blood Urea Nitrogen (**mg/dL)	20.51	4.0%
	62.6	4.6%
**Creatinine (**mg/dL)	1.59	3.1%
	4.32	3.4%
**Triglycerides (**mg/dL)	97	2.8%
	222	2.0%
**Total cholesterol (**mg/dL)	144	2.0%
	190	2.2%
**SGOT (U/L)**	51.3	3.8%
	171	4.8%
**SGPT (U/L)**	55.8	3.2%
	109	3.0%
**ALP (IU/L)**	121	3.8%
	296	4.9%
**HDL Cholesterol(**mg/dL)	43	1.8%
**Gamma-GlutamylTransferase (GGT) (IU/ml)**	29.7	4.0%
	75.4	3.0%

^1^The inter assay variation describes the variation of results obtained from repeated experiments. It is expressed by inter assay coefficient of variation (inter assay CV) to monitor the precision of results between different assays.

### Biostatistical analysis

Categorical variables using number, percent (%) and continuous variables by the mean and standard deviation (SD) have been described. The t-test was used to compare continuous variables in 2 groups. Moreover, the Chi-square test was used to examine the association between 2 categorical variables. The multivariable binary logistic regression was used to estimate the adjusted odds ratio (ORs) and determine the relationship between some of the factors with hypertension, diabetes, and heart diseases. We, here, reported the prevalence and 95% confidence intervals of major risk factors to NCDs. In present study, using statistical methods such as regression models, the relationship between independent variables and the desired outcomes in different categories of confounding variables were calculated separately or controlled by analysis stratification. All statistical tests were two-sided as well as all analyses were carried out using the SPSS software; A P < 0.05 was considered as statistically significant.

## Results

**Fig 3:**
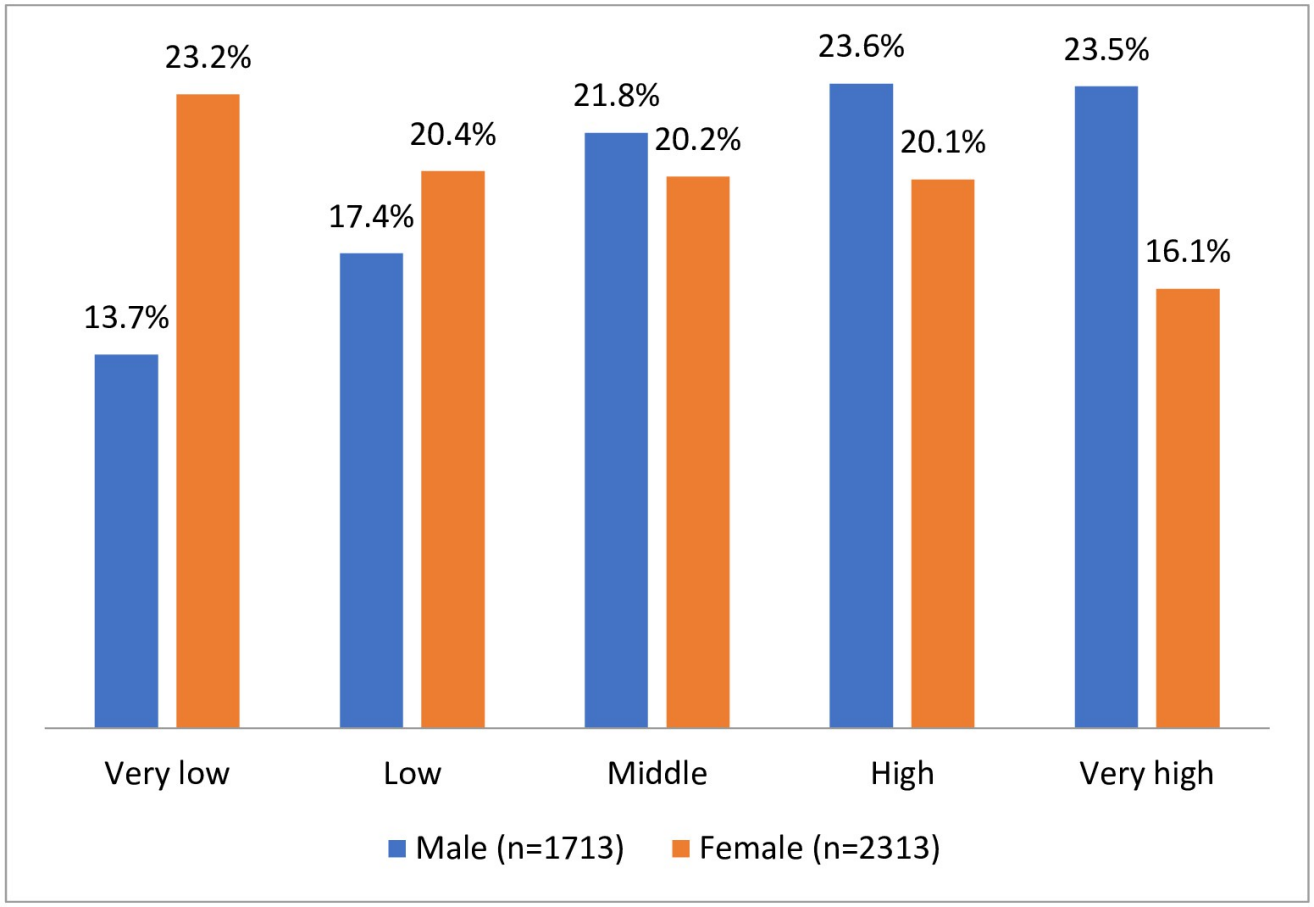
Socioeconomic status of population in the cohort of Bandar Kong.

Data was cleaned up and validated. Participation rate was almost 94% and declining to participate was due to unwillingness, lack of confidence and feeling unwell. The response rate was 99%. Baseline demographic characteristics were shown in Table 5. Out of 4063 participants, 42.5% were male, 57.5 female, 84.8% were resident of urban area and the remaining, 15.2%, of rural area. The marriage rate was 89.5% and 67.8% were illiterate or with inadequate literacy. One third of the participants was in the age group of 40–49 years. All the participants were native where 44.2% of them as an employee and remaining 47.7%, as housewife. Housing ownership was 85%. Participants’ baseline characteristics by gender and prevalence of self-reported health conditions have been displayed in Table 6. Of note, 35.4% of men and 37.0% of women were overweight, while, 30.4% of women were obese compared to 16.1% obesity in men. Hypertension was observed in 27.3% of the participants (27.3% (95% CI: 25.9–28.6%) of whom the highest proportion (64.7%) was found in age above 65 years. The mean systolic and diastolic BP were 118.7 ± 17.5 (SD) mmHg and 76.9 ± 10.4 (SD) mmHg, respectively. The prevalence of hypertension was increased with age (p < 0.001). Of the 1109 (27.3%) hypertensive participants, 731 (65.9%) were aware of their hypertension. As shown in Fig 3, mean hip and waist circumferences were less in men than in women. The most prevalent self-reported NCDs were listed in Table 6. We found that the majority of reported NCDs were more prevalent in women than in men. Disparities in health due to differences in socioeconomic status (SES) was identified as a strong social determinant of health. In the present study, the prevalence of multimorbidity of 3 or more NCDs were 8% (No. 326) and 6% (No.240), respectively. Association of the co-occurrence of obesity risk factors with NCD multimorbidity was more significant (clustering of 3 and more NCDs as 6% and 5%) compared with co-existence of high cholesterol (clustering of 3 and more NCDs as 1.5% and 1%) and smoking (clustering of 3 and more NCDs as 1,25% and 0.8%) risk factors with multimorbidity. Consistent with the literature [28–30], we calculated SES index based on owning car, car price, house ownership, house area, owning computer (PC & Notebook), owning Dishwasher machine, number of travels per year and job title categorized into five groups, as shown in Fig 3. By Dec 30, 2019, we completed the third wave of follow-up with a participation above 99%. An overview of outcomes has been displayed in Table 7.

**Table 5:** Baseline socio-demographic characteristics of participants.

Variable	No (%)	Mean ± SD
Sex		
Male	1725 (42.5)	
Female	2334 (57.5)	
Age Group		
35–39 yr	940 (23.2)	
40–44 yr	747 (18.4)	
45–49 yr	689 (17)	
50–54 yr	556 (13.7)	
55–59 yr	510 (12.6)	
60–64	370 (9.1)	
>= 65 yr	247 (6.1)	
Age		48.25 ± 9.4
Education		
illiterate	1798 (44.3)	
Primary School	954 (23.5)	
Secondary School	511 (12.6)	
Diploma	460 (11.3)	
University	336 (8.28)	
Education Years		5.82 ± 4.8
Marital Status		
Single	98 (2.4)	
Married	3631 (89.5)	
Widowed	254 (6.3)	
Divorced	76 (1.9)	
Age at first marriage		20.85 ± 5.2
Marriage with Relatives		
No	2626 (64.7)	
Yes—with first cousin	734 (18.1)	
Yes—with second cousin	598 (14.7)	
Missing	101 (2.5)	
Employment		
Employed	1794 (44.2)	
Unemployed	329 (8.1)	
Housewife	1936 (47.7)	
Housing Ownership		
Owned	3439 (84.7)	
Leased	333 (8.2)	
Other	262 (6.5)	
Missing	25 (0.6)	
Floor area of residence		
less than 100 m^2^	1386 (34.1)	
100–200 m^2^	2231 (55.0)	
more than 200 m^2^	417 (10.3)	
Missing	25 (0.6)	
Residential Status		
urban	3442 (84.8)	
rural	617 (15.2)	

**Table 6:** Participants’ baseline characteristics by gender and prevalence of self-reported health conditions.

Domain	Variable	Men	Women	P-value^1^
Lifestyle	Cigarette smoking	33.8%	0.3%	<0.001
	Hookah smoking	23.2%	17.3%	<0.001
	Alcohol drinking	12.5%	0.2%	<0.001
	Drug use	9.7%	0.5%	<0.001
	No toothbrushing	6.5%	3.7%	<0.001
Anthropometrics	BMI (Mean ± SD)	25.8 ± 4.5	27.8 ± 5.2	<0.001
	Underweight	5.6%	3.1%	<0.001
	Normal	43.0%	29.5%	
	Overweight	35.4%	37.0%	
	Obese	16.1%	30.4%	
Physical Examination	Systolic Blood Pressure (Mean ± SD)	120.51 ± 16.5	117.23 ± 18	<0.001
	Diastolic Blood Pressure (Mean ± SD)	78.40 ± 9.9	75.77 ± 10.8	<0.001
Laboratory Findings	Fasting Blood Sugar (Mean ± SD)	105.62 ± 37.7	109.96 ± 46.4	<0.001
	Total Cholesterol (Mean ± SD)	198.99 ± 42.3	204.79 ± 42.3	<0.001
	TG (mg/dL)	148.98 ± 42.3	127.23 ± 67.9	<0.001
	HDL (mg/dL)	44.41 ± 9.6	50.48 ± 10.9	<0.001
	LDL (mg/dL)	125.15 ± 33.3	129 ± 35.7	<0.001
Self-reported disease (diagnosed by physician)	Diabetes mellitus	17.4%	12.4%	<0.001
	Hypertension	14.6%	21.0%	<0.001
	MI	2.6%	1.0%	<0.001
	Stroke	0.8%	0.9%	0.725
	Renal Failure	0.4%	0.8%	0.109
	Fatty Liver	12.0%	11.1%	0.358
	Thyroid	3.2%	13.9%	<0.001
	Rheumatic disease	1.1%	5.5%	<0.001
	Chronic lung disease (Asthma/ Tuberculosis)	2.5%	3.7%	0.034
	Breast cancer	--	0.6%	--
	Cervical cancer	--	0.2%	--
	Prostate cancer	0.0%	--	--
	Colorectal cancer	0.2%	0.1%	0.229
	Depression	0.8%	1.7%	0.008
	Psychiatric disorders	2.0%	3.4%	0.006
	Osteoporosis	20 (0.5%)	262 (6.5%)	<0.001
	Fracture	106 (2.6%)	141(3.5%)	0.947

^1^The independent samples t-test was used to determine if there is a significant difference between the means of two groups, the chi-squared test was used to determine whether there is an association between 2 categorical variables.

**Table 7:** The most common chronic NCDs diagnosed during the first and second annual follow-ups.

Chronic NCDs	1^st^ anuual follow-up (%)	2^nd^ annual follow-ups (%)
Hypertension	68 (37%)	72 (53%)
Diabetes	48 (26%)	29 (21%)
Ischemic Heart disease	17 (9.4%)	11(8%)
Stroke	15 (8.3%)	10 (7%)
Cancers	7 (3.8%)	1 (0.7%)
COPD	6 (3.3%)	4 (3%)
Renal failure	1 (0.5%)	-
Others	6 (3.3%)	1 (0.7%)
Death (Due to NCDS)	12 (6.6%)	14 (10%)
Total outcome	180	136

### Determinates of some of factors affecting hypertension, diabetes, and cardiac vascular disease

Association between the increase of risk of hypertension and age, being married, being a widow/divorced, and living in a rural area was found by adjusted ORs (Table 8). Based on Table 2, the aged 55–64 people were more likely to be at risk of hypertension compared to the aged 35–44 people (AOR = 1.30, 95% CI: 1.07,1.57). The odds to be at risk of hypertension was 1.81 and 2.93 times higher for married and widowed/divorced people than the single ones, respectively (AOR = 1.81, 95% CI: 1.06,3.09; AOR = 2.93, 95% CI: 1.64,5.22). Besides, living in a rural area increased the odds of having hypertension by 1.33 (AOR = 1.33,95% CI: 1.09,1.61).

**Table 8:** Multivariable analysis of some of the factors affecting hypertension, diabetes, and heart diseases in the current study.

Variables			Hypertension^1^		Diabetes^2^		Cardiac Vascular Disease^3^	
			*Adjusted OR (95%CI)	P-value	*Adjusted OR (95%CI)	P-value	*Adjusted OR (95%CI)	P-value
Sex	(Ref: male)		0.94 (0.81,1.208)	0.391	1.38(1.16,1.64)	<0.001^+^	0.83(0.65,1.04)	0.118
Age	(Ref: 35–44)	45–54	1.03(0.87,1.23)	0.679	1.04(0.85,1.27)	0.684	1.14(0.86,1.52)	0.348
		55–64	1.30(1.07,1.57)	0.008^+^	1.09(0.87,1.38)	0.427	1.58(1.17,2.13)	0.003^+^
		≥65	1.31(0.96,1.79)	0.084	1.02(0.70,1.49)	0.888	1.35(0.84,2.17)	0.215
Marital status	(Ref: single)	Married	1.81(1.06,3.09)	0.030^+^	2.02(1.04,3.94)	0.038^+^	2.71(0.85,8.67)	0.091
		Widow/divorced	2.93(1.64,5.22)	<0.001^+^	2.59(1.27,5.28)	0.008^+^	4.42(1.33,14.72)	0.015^+^
Education	(Ref: >12years)	≤5 years	1.05(0.80,1.38)	0.707	1.06(0.76,1.47)	0.706	1.45(0.90,2.34)	0.122
		6-12years	0.86(0.64,1.15)	0.311	1.04(0.74,1.47)	0.808	1.05(0.63,1.76)	0.839
Residence	(Ref: Urban)		1.33(1.09,1.61)	0.004^+^	1.28(1.02,1.61)	0.027^+^	0.91(0.66,1.25)	0.576
BMI	(Ref:18.5–24.9)	≤ 18.4	0.71 (0.44,1.16)	0.176	0.90(0.52,1.55)	0.709	1.44(0.74,2.82)	0.280
		25.0–29.9	1.17(0.99,1.39)	0.053	1.13(0.93,1.38)	0.199	1.41(1.07,1.85)	0.014^+^
		30.0–34.9	1.06(0.86,1.31)	0.571	0.96(0.74,1.23)	0.759	1.46(1.05,2.03)	0.023^+^
		≥35	1.12(0.82,1.53)	0.446	1.28(0.91,1.82)	0.153	1.43(0.89,2.30)	0.136
Smoke Cigarette	(Ref:Non-smoker)	Current	0.92(0.71,1.20)	0.555	1.07(0.79,1.44)	0.633	1.03(0.69,1.55)	0.850
		ex-smoker	1.11(0.82,1.50)	0.467	1.21(0.86,1.71)	0.258	0.72(0.42,1.23)	0.240
Physical activity (Daily MET)^++^	(Ref: ≥45)	24–36.5	1.03(0.81,1.29)	0.831	1.22(0.92,1.62)	0.154	1.36(0.92,2.00)	0.115
		36.6–44.9	1.03(0.83,1.27)	0.771	1.10(0.86,1.42)	0.431	1.16(0.81,1.65)	0.407

*Adjusted Odds Ratio.

^+^ Significant at 0.05 level.

^++^Metabolic Equivalent of Task.

^1^Self-reported hypertension (HTN), anti-hypertensive medicine use, or a new hypertensive case were all used to define hypertension (HTN). People with a SBP of 140 mmHg or a DBP of 80 mmHg, or both, were considered as new hypertensive cases.

^2^T2DM was defined by the American Diabetes Association (ADA) as FPG 126 mg/dl in the first evaluation phase of study or the use of anti-hyperglycemic medications or self-reported disease.

^3^Cardiovascular disease (CVD) is defined by the presence coronary heart disease (CHD) or stroke by self-reporting. By definition, Osteoporosis and fracture were self-reported.

Moreover, association between an increase of risk of diabetes and being female, being married, being a widow/divorced, and living in a rural area was found. By adjusting the effect of other variables, being female was related to a higher risk of diabetes (AOR = 1.38, 95% CI: 1.16,1.64). The odds to be at risk of diabetes was 2.02 and 2.59 times higher for married and widowed/divorced people than the single ones, respectively (AOR = 2.02, 95% CI: 1.04,3.94; AOR = 2.93, 95% CI: 1.27,5.28). Moreover, living in a rural area increased the odds of having hypertension by 1.33 (AOR = 1.33,95% CI: 1.09,1.61).

In addition, age, being a widow/divorced and BMI increased the odds of having cardiac vascular disease. By controlling the effect of other variables, the aged 55–64 people were more likely to be at risk of cardiac vascular disease compared to the aged 35–44 people (AOR = 1.58, 95% CI: 1.17,2.13). Besides, the odds of having cardiac vascular disease was 4.42 times higher for widowed/divorced people than the single ones (AOR = 4.42, 95% CI: 1.33,14.72). Furthermore, the odds of having the cardiac vascular disease was higher for people who had BMI≥25 than people with 18.5≤BMI≤ 24.9. Lower Cardiovascular diseases, serum level of FBS and higher HDL level in sailors/fishermen compared to other job groups were significant (p-value <0.001) along with more findings in detailed have been represented and summarized in Table 9.

**Table 9:** Comparison of major clinical and laboratory findings among jobless people, sailors/fishermen, employees and other occupations.

	Jobless^1^ (n = 2254)	Sailor/Fisherman (n = 340)	Employee (n = 295)	Other jobs (n = 1157)	P- value^2^
Clinical findings					
Cardiac Disease, n (%)	229(10.2)	16(4.7)	10(3.4)	68(5.9)	<0.001
MI, n (%)	46(2.0)	6(1.8)	2(0.7)	18(1.6)	0.353
BPS(mean ± SD)	119.49±18.69	118.44±14.43	114.07±13.96	118.34±16.39	<0.001
BPD (mean ± SD)	76.93±10.79	77.74±9.25	75.65±10.08	76.88±10.12	0.092
Blood Analysis (mean ± SD)					
FBS	111.50±47.65	101.26±31.09	100.42±26.52	105.56±38.84	<0.001
Cholesterol	203.97±42.98	201.28±49.01	202.74±36.76	199.47±40.20	0.031
Triglycerides	130.36±70.76	156.03±158.24	140.74±94.55	141.86±85.22	<0.001
LDL cholesterol	128.50±36.16	126.64±31.81	128.00±30.12	125.84±33.20	0.186
HDL cholesterol	49.63±10.89	44.49±9.46	46.65±10.14	46.01±10.46	<0.001
S.G.O.T (AST)	20.89±11.08	23.76±9.45	24.29±15.53	22.87±10.10	<0.001
S.G.P.T (ALT)	23.77±19.02	33.78±22.99	33.88±27.58	30.16±22.26	<0.001
Alkaline Phosphatase	203.84±70.85	197.99±50.39	180.50±47.16	199.19±66.85	<0.001
Urine Analysis					
Blood (positive), n (%)	360(16.1)	34(10.1)	23(7.9)	136(11.8)	<0.001
Protein (positive), n (%)	125(5.6)	16(4.8)	5(1.7)	46(5.0)	<0.001
Glucose (positive),n(%)	235(10.5)	16(4.8)	15(5.2)	92(8.0)	<0.001

^1^ Unemployed / Housewife / Retired;

^2^ ANOVA was used to determine whether there are any statistically significant differences between the means of a continuous variable in 4 groups, and the chi-squared test was used to determine whether there is an association between 2 categorical variables.

As observed in Table 10, intake of fruit, vegetables, and dairy was less than two servings per day in 9.2%, 13%, and 58.3% of the participants, respectively. Eating less than one serving of fish per week was reported by 6.3% of the participants. Soft drinks were served more than two servings per week by 79.7% and 53.6% had more than four servings of sugar per day. Intake of palm oil was equal or more than one serving per day in 26.9% of the participants; 75.3% consumed date more than two servings per day.

**Table 10:** Baseline dietary intake of participants in Bandar Kong cohort study.

	All	Male, N (%)	Female, N (%)	P value
Eating less than two servings of fruits per day	371 (9.2)	121 (7.1)	250 (10.8)	<0.001
Eating less than two servings of vegetables per day	524 (13)	196 (11.4)	328 (14.2)	0.011
Eating less than two servings of dairy per day	2353 (58.3)	870 (50.7)	1483 (64)	<0.001
Eating less than one serving of fish per week	255 (6.3)	74 (4.3)	181 (7.8)	<0.001
Eating more than two servings of soft drink per week	3212 (79.7)	1162 (67.7)	2050 (88.5)	<0.001
Eating more than four servings of sugar per day	2162 (53.6)	1036 (60.4)	1126 (48.6)	<0.001
Eating equal or more than one serving of palm oil per day	1083 (26.9)	462 (26.9)	621 (26.8)	0.938
Eating more than two servings of date per day	3038 (75.3)	1350 (78.8)	1688 (78.9)	<0.001

## Strengths and limitations

Here, in order to improve the external validity and to increase the generalizability of the study results to the target community, the participants were enrolled from the both gender groups with diverse education and jobs as well as various socio-economic status.

The most important strengths of the study include: a) The first population-based prospective cohort investigation of NCDs in Southern coastlines, b) Establish of valuable infrastructure for future large studies, c) Prospective investigation on lifestyle and risk factors to NCDs in sailors/fishermen and Islands inhabitants through well-trained staff under close supervision, d) set up a large biobank of various specimens to multi-omics study, e) minimum loss to follow-up f) connecting with corresponding electronic health records (SIB system), g) being open cohort, h) extensive data collection at baseline. Our valuable experience may serve as a model for neighborhood countries in the Middle East and North Africa harboring similar climate, socioeconomic, cultural and life style status.

We realized the weaknesses of the study as follows: a) Sample size, b) Budget limitation, c) Cultural and religious restrictions to collect data on sexual transmitted diseases, d) lower social activities of housebound individuals, e) little information on family tree f) failure to collect stool to study the impact of gut microbiota in NCDs development, g) limited to participants aged 35–70, h) information bias due to self-reported questionnaire.

## Conclusion

Kong cohort study is the first and unique ongoing prospective population-based cohort study which has been undertaken in sea ports and coastal areas of southern Iran. Iran has undergone a period of demographic, health and life style transition. Prospective studies are the ideal tools to follow these transitions and their impacts on health status. Kong cohort study with well-defined roadmap and standard protocols, suitable data quality and persistent annual follow-ups in southern Iran provided proper setting for investigating NCDs.

Noteworthy, the participation and response rates were 94% and 99%, respectively, as compared to participation rate of 70% in the Golestan, 94.9% in the Yazd and 57.5% in the Tehran Lipid and Glucose (TCS) cohort studies [13, 31, 32]. Current study revealed that compared to men, physical inactivity, higher BMI, overweight and obesity were more prevalent in women, the same as reported worldwide [33–35]. Unhealthy life style including physical inactivity and smoking was common in southern Iran. In the present study, the alteration of nutrition pattern of the participants was reflected in the low intake of fruit, vegetables, dairy and fish associated with high servings of palm oil and sugar. These findings are in concordance with high incidence of NCDs in Kong cohort study and southern Iran.

We recorded 28 deaths during first and second annual follow-ups till now. The main point is the higher prevalence of cardiovascular diseases including above 40% of the recorded deaths. These findings are consistent with national estimates. Here, Women’s participation rate was higher, the same as happened in PERSIAN Cohort Studies, however, at baseline, we observed diverse demographic and clinical characteristics in current study as compared with other national cohorts. For instance, the prevalence of alcohol drinking was higher in Kong cohort compared to Golestan and TCS (12.5%, 0.7% and 7.9%, respectively) [28, 29]. Of note, our study showed higher prevalence of diabetes mellitus, while lower of that for hypertension as compared to Pars cohort study (17.4%, 9.4% for diabetes and 26.9%, 21% for hypertension, respectively) [36]. The prevalence of NCD multimorbidity is growing in low- and middle-income countries and being estimated 7.8%. In future, multimorbidity will be an extreme challenge for both developed and developing countries [37, 38]. Awareness on multimorbidity patterns is of important implications for prevention, diagnosis, and management. In the present study, our epidemiologic data revealed a high NCD multimorbidity that could be implicated in health policies and developing guidelines and designing strategies to reduce the burden of the disease.

The ongoing longitudinal cohort possesses the potential to survey and evaluate the lifestyle changes over the lifetime. We planned to increase the sample size and improve the cohort quality during annual follow-ups to generate fresh and up to date data for tackling NCDs. The key justification for running this cohort was to cover various geographical regions including mountains and coastlines in vicinity of each other with hot and humid climate almost throughout the year, which made it a unique environment likely leading to development of region-specific risk factors and NCDs.

In this study, the primary findings demonstrated high prevalence of behavioral modifiable risk factors. Therefore, conducting population-specific interventions plus nested case-control studies along with persistent monitoring the trend of development of chronic diseases could generate valuable evidence-based policies in controlling the risk factors. Prevention of premature deaths due to NCD and decrease of associated health care expenses will be the main objectives of health policy. The consequences of such investigations would aid policy makers to plan and implement cost-effective facility within the situation of primary health care in Iran, which could serve as a model for neighboring countries through.

At last, we effectively established a population-based prospective cohort of adults that highlighted the requirement for evolving NCD intervention programs, and launching active surveillance for operative planning, application and evaluation. Comprehensive control of NCD risk factors has turn into an absolute requisite worldwide. Blood specimens collected at baseline and in each follow-up will be a distinctive and potent resource for investigating the association between major risk factors with NCDs and exploring further original biomarkers. The ultimate goal is sustaining a healthy life style which would promise prevention and reduction the burden of NCDs.

## Supporting information

S1 File Questionnaire. (PDF)

S2 File Data dictionary for baseline variables. (PDF)

S3 File Rationale, objectives, and design. (PDF)

S4 File Questionnaire in original language. (PDF)

## Abbreviations

95% CI: 95% Confidence Interval
ESR: erythrocyte sedimentation rate
FFQ: food frequency questionnaire
NCDs: non-communicable diseases
OR: Odds Ratio
PCID: PERSIAN Cohort Identification
PERSIAN: Prospective Epidemiological Research Studies in IrAN
QA: Quality assurance
QC: Quality control
SES: socioeconomic status
WHO: World Health Organization

## References

[1] GBD 2015 Risk Factors Collaborators. Global, regional, and national comparative risk assessment of 79 behavioural, environmental and occupational, and metabolic risks or clusters of risks, 1990–2015: a systematic analysis for the Global Burden of Disease Study 2015. Lancet. 2016; 388:1659–1724. doi: 10.1016/S0140-6736(16)31679-8 27733284 PMC5388856

[2] WHO-EMRO. Non-communicable disease in MROhttp://www.emro.who.int/noncommunicablediseases/diseases/diseases.html. Date of access May 2016. 2014

[3] Global health estimates 2014 summary tables: Estimated deaths by cause, sex and WHO Member State 2012. Geneva: World Health Organization; 2014. (http://www.who.int/healthinfo/global_burden_disease/estimates/en/index1.html, accessed 12 October 2014).

[4] Hunter DJ, Reddy KS. Non-communicable diseases. N Engl J Med. 2013; 369: 1336–43. doi: 10.1056/NEJMra1109345 24088093

[5] World Health Organization. Global status report on non-communicable diseases (2014) (last accessed 14 July 2016). http://apps.who.int/iris/bitstream/10665/148114/1/9789241564854_eng.pdf

[6] Narayan KM, Ali MK, Koplan JP. Global non-communicable diseases-Where worlds meet. N Engl J Med. 2010; 363:1196–8. doi: 10.1056/NEJMp1002024 20860499

[7] Forouzanfar MH, Sepanlou SG, Shahraz S, Daniel D, Naghavi P, Pourmalek F, et al. Evaluating causes of death and morbidity in Iran, global burden of diseases, injuries, and risk factors study 2010. Arch Iran Med. 2014; 17:304–20. 24784860

[8] Naghavi M, Shahraz S, Sepanlou SG, Daniel D, Naghavi P, Pourmalek F, et al. Health transition in Iran toward chronic diseases based on results of Global burden of disease 2010. Arch Iran Med. 2014; 17:321–335 24784861

[9] Nekoei Moghadam M, Ahmadizadeh Fini E, Khanjani N, Vali L, Rafiezad E, Dashtyian MK, et al. Assessment of Non-Communicable Disease Risk Factors in Hormozgan Province. Life Sci J. 2013;10: 405–408.

[10] Ahmadizadeh Fini A, Aghamolaei T, Dehghani M, Zare S, Madani A. Environmental tobacco smoke (ETS) exposure in people over 15 years old, Bandar Abbas, Iran. Hormozgan Med J. 2011; 1:42–47.

[11] Sepanlou SG, Malekzadeh F, Naghavi M, Forouzanfar MH, Shahraz S, Moradi-Lakeh M, et al. Trend of Gastrointestinal and Liver Diseases in Iran: Results of the Global Burden of Disease Study, 2010. Middle East J Dig Dis. 2015;7:121–37. 26396715 PMC4560627

[12] Bijani A, Ghadimi R, Mikaniki E, Kheirkhah F, Mozaffarpur SA, Motallebnejad M, et al. Cohort Profile Update: The Amirkola Health and Ageing Project (AHAP). Caspian J Intern Med. 2017;8:205–212. doi: 10.22088/cjim.8.3.205 28932373 PMC5596192

[13] Azizi F, Rahmani M, Emami H, Mirmiran P, Hajipour R, Madjid M, et al. Cardiovascular risk factors in an Iranian urban population: Tehran lipid and glucose study (phase 1). Soz Praventivmed. 2002;47:408–26. doi: 10.1007/s000380200008 12643001

[14] Moghadam MN, Sadeghi V, Parva S. Weaknesses and challenges of primary healthcare system in Iran: a review. Int J Health Plann Manag. 2012; 27:121–31. doi: 10.1002/hpm.1105 22009801

[15] Countdown NCD 2030 collaborators. NCD Countdown 2030: Worldwide trends in non-communicable disease mortality and progress towards Sustainable Development Goal target 3.4. Lancet. 2018, 392, 1072–1088. doi: 10.1016/S0140-6736(18)31992-5 30264707

[16] Goorakani Y, Sedigh Rahimabadi M, Dehghan A, Kazemi M, Rostami Chijan M, Mostafa Bijani M, et al. Correlation of resting heart rate with anthropometric factors and serum biomarkers in a population-based study: Fasa PERSIAN cohort study. BMC Cardiovasc Disord. 2020;20:319. doi: 10.1186/s12872-020-01594-y 32631242 PMC7336436

[17] Hakimi H, Ahmadi J, Vakilian A, Jamalizadeh A, Kamyab Z, Mehran M, et al. The profile of Rafsanjan Cohort Study. Eur J Epidemiol. 28 July, 2020. Available from doi: 10.1007/s10654-020-00668-7 32725579

[18] Ghayour-Mobarhan M, Shapouri-Moghaddam A, Azimi-Nezhad M, Esmaeili H, Parizadeh SMR, Safarian M, et al. The relationship between established coronary risk factors and serum copper and zinc concentrations in a large Persian cohort. J Trace Elem Med Biol. 2009; 23:167–175. doi: 10.1016/j.jtemb.2009.03.006 19486826

[19] Mansour-Ghanaei R, Mansour-Ghanaei F, Naghipour M, Joukar F. Biochemical markers and lipid profile in nonalcoholic fatty liver disease patients in the PERSIAN Guilan cohort study (PGCS). Iran J Family Med Prim Care.2019;8:923–928. doi: 10.4103/jfmpc.jfmpc_243_18 31041226 PMC6482810

[20] Somi MH, Nikniaz Z, Ostadrahimi A, Eftekhar Sadat AT, Faramarzi E. Is normal body mass index a good indicator of metabolic health in Azar cohort population? J Cardiovasc Thorac Res. 2019;11(1):53–60. doi: 10.15171/jcvtr.2019.09 31024673 PMC6477109

[21] Management and program Organization of Hormozgan province. Statistic calendar of Hormozgan province, Country embassy, Province council of Hormozgan, Statistics and information department 2007.

[22] Moghimi A. Urban Geomorphology, 2ed edition, University of Tehran press, Tehran. 2008.

[23] Floor W. Bandar Abbas: The natural trade gateway of southeast Iran, Washington. 2011.

[24] Amanolahi S. “A note and ethnicity and ethnic groups in Iran”, in Iran and the Caucasus. Iran & the Caucasus. Brill.2005.

[25] Postchi H, Eghtesad S, Mohammadi M, Rozafzay F, Mahmoodi Z, Shayanrad A. Prospective epidemiologic research studies in Iran, PERSIAN cohort, study protocol. Edition one, editor. Tehran. Mir Mah publication. 2016.

[26] Postchi H, Eghtesad A, Kamangar F, Etemadi A, Hekmatdoost A, Keshtkar AA, et al. Prospective epidemiological research studies in IrAN (the PERSIAN cohort): rationale, objective and design. Am J Epidemiol. 2018; 187:647–55. doi: 10.1093/aje/kwx314 29145581 PMC6279089

[27] Dean AG, Sullivan KM, Soe MM. OpenEpi: Open Source Epidemiologic Statistics for Public Health, Version. www.OpenEpi.com, updated 2013/04/ 06, accessed 2017/05/06.

[28] Adler NE, Ostrove JM. Socioeconomic status and health: What we know and what we don’t. Ann N Y Acad Sci.1999; 896:3–15. doi: 10.1111/j.1749-6632.1999.tb08101.x 10681884

[29] Lund C, Breen A, Flisher AJ, Kakuma R, Corrigall J, Joska JA, et al. Poverty and common mental disorders in low- and middle-income countries: A systematic review. Soc Sci Med. 2010; 71:517–528. doi: 10.1016/j.socscimed.2010.04.027 20621748 PMC4991761

[30] Kagamimori S, Gaina A, Nasermoaddeli A. Socioeconomic status and health in the Japanese population. Soc Sci Med. 2009; 68:2152–2160. doi: 10.1016/j.socscimed.2009.03.030 19375838

[31] Pourshams A, Khademi H, Malekshah AF, Islami F, Nouraei M, Sadjadi AR, et al. Cohort Profile: The Golestan Cohort Study-a prospective study of oesophageal cancer in northern Iran. Int J Epidemiol. 2010; 39:52–9. doi: 10.1093/ije/dyp161 19332502 PMC3709199

[32] Mirzaei M, Salehi-Abargouei A, Mirzaei M, Mohsenpour MA. Cohort Profile: The Yazd Health Study (YaHS): a population-based study of adults aged 20–70 years (study design and baseline population data). Int J Epidemiol. 2017; Nov 24:1–10. doi: 10.1093/ije/dyx231 29186588

[33] Alhyas L, McKay A, Balasanthiran A, Majeed A. Prevalences of overweight, obesity, hyperglycaemia, hypertension and dyslipidaemia in the Gulf: systematic review. JRSMShort Rep. 2011; 2(7):55. 21847437 10.1258/shorts.2011.011019 PMC3147233

[34] Beaglehole R, Bonita R, Horton R, Adams C, Alleyne G, Asaria P, et al. Priority actions for the non-communicable disease crisis. The Lancet. 2011; 377(9775):1438–47. doi: 10.1016/S0140-6736(11)60393-0 21474174

[35] Sithey G, Li M, Thow AM. Strengthening noncommunicable disease policy with lessons from Bhutan: linking gross national happiness and health policy action. Journal of Public Health Policy. 2018;39(3):327–42. doi: 10.1057/s41271-018-0135-y 29950574

[36] Gandomkar A, Poustchi H, Moini M, Moghadami M, Imanieh H, Fattahi MR, et al. Pars cohort study of non-communicable diseases in Iran: protocol and preliminary results. Int J Public Health. 2017;62:397–406. doi: 10.1007/s00038-016-0848-2 27349480

[37] Prados-Torres A, Poblador-Plou B, Gimeno-Miguel A, Calderón-Larrañaga A, Poncel-Falcó A, Gimeno-Feliú L A, et al. Cohort profile: the epidemiology of chronic diseases and multimorbidity. The EpiChron Cohort Study. International journal of epidemiology. 2018; 47:382–384. doi: 10.1093/ije/dyx259 29346556 PMC5913592

[38] Wang Yuan-Pang, Nunes Bruno P., Coêlho Bruno M., Santana Geilson L., do Nascimento Carla F., Viana Maria Carmen, et al. Multilevel Analysis of the patterns of physical-Mental Multimorbidity in General population of são paulo Metropolitan Area, Brazil. Scientific report. 2019;9: 2390. doi: 10.1038/s41598-019-39326-8 30787376 PMC6382818

