# Correction: Cohort profile: Bandar Kong prospective study of chronic non-communicable diseases

**DOI:** 10.1371/journal.pone.0315430

**Published:** 2024-12-05

**Authors:** The PLOS ONE Staff

## Notice of Republication

This article was republished on January 5, 2023, to correct for errors in the Data Availability statement. The publisher apologizes for the errors. Please download this article again to view the correct version. The originally published, uncorrected article and the republished, corrected article are provided here for reference.

## Supporting information

S1 File Originally published, uncorrected article. (PDF)

S2 File Republished, corrected article. (PDF)
