# Supplementary material for: Cohort profile: Bandar Kong prospective study of chronic non-communicable diseases
Source: PLoS One. 2022 May 12;17(5):e0265388. doi: 10.1371/journal.pone.0265388 (PMC9098057; doi:10.1371/journal.pone.0265388)
Supplement: S1 File — (PDF) [file pone.0265388.s001.pdf]

PERSIAN Cohort Pilot Study
Main Questionnaire

Personal Information	
**Personal Identification**	
First Name:	Last Name:
National ID Number:	Father's Name:
Date of Birth:	Gender: 1. Male☐ 2. Female ☐
Paternal Ethnicity:<br>1. Fars, 2. Azari, 3. Balouch, 4. Kurd, 5. Lor, 6. Arab, 7, Turk, 8. Talesh, 9. Zabli, 10. Gilak, 11. Turk Nomads, 12. Arab Nomads	Maternal Ethnicity:<br>1. Fars, 2. Azari, 3. Balouch, 4. Kurd, 5. Lor, 6. Arab, 7, Turk, 8. Talesh, 9. Zabli, 10. Gilak, 11. Turk Nomads, 12. Arab Nomads
**Place of Birth**	
Province:	County:
City:	Village:
**General Information**	
Name of Individual Completing the Questionnaire (if not the participant him/herself):	IRPC: ☐☐☐☐☐☐☐☐☐
Home Phone: ☐☐☐☐☐☐☐☐☐☐	Zip Code: ☐☐☐☐☐☐☐☐☐☐
Mobile Phone: ☐☐☐☐☐☐☐☐☐☐	Email:
**Complete Address**	
Province:	County:
City:	Village:
Street:	Alley:
House Number:	
Longitude:	Latitude:
Years of Education: ___ ___	
Last educational degree obtained:<br>1. Elementary<br>2. Middle school<br>3. High school diploma<br>4. Associate degree<br>5. Bachelor's degree<br>6. Master's degree<br>7. PhD	
The interviewee's position/relationship in the household:<br>1. Father, 2. Mother, 3. Child, 4. Grandfather or grandmother, 5. Uncle/Aunt (father's side), 6. Uncle/aunt (mother's side),<br>7.Other _________________________	
Marital Status: 1. Single, 2. Married, 3. Widowed, 4. Divorced, 5. Other____________	

Contact information of two close family members/friends	
2.<br>Name_______________________________<br>Relationship to interviewee__________________<br>Home phone number______________________<br>Cell phone number________________________	1.<br>Name_______________________________<br>Relationship to interviewee__________________<br>Home phone number______________________<br>Cell phone number________________________

Socioeconomic Status

The family's current living situation:

1. Home owner
2. Lease or Rent
3. Company home (governmental or private company)
4. Relative's house (only if no charge is paid for rent)
5. Other________________________________

House Size (in m^2)—excluding any of the following: porches, gardens, yards, garage, or any place where house animals are kept: ___________________________

Number of bedrooms in current home (kitchen and living room not included):	Number of individuals living in your current home:

Do you have the following household items in your household?

Separate Freezer	Yes	No
Washing Machine	Yes	No
Dish Washer	Yes	No
Computer/ Laptop	Yes	No
Internet Access	Yes	No
Motorcycle	Yes	No
Car	Yes:<br>1.< 20m Tomans<br>2. 20-50m Tomans<br>3. 50-100m Tomans<br>4. >100m Tomans	No

PERSIAN Cohort

Vacuum Cleaner	Yes	No
Color TV	Yes<br>1. Regular<br>2. Plasma	No
Shower inside the house	Yes	No
Do use the following household items?		
Mobile Phone	Yes	No
Desktop Computer	Yes	No
Laptop	Yes	No
Internet Access	Yes	No
Car	Yes:<br>1. 20m Tomans<br>2. 20-50m Tomans<br>3. 50-100m Tomans<br>4. > 100m Tomans	No
Number of books you have read in the past year (excluding school books, those required for your job, and religious books) ___ ___ 0. None		
Number of international trips you have had:<br>1. Pilgrimage ☐☐ 2. Non-pilgrimage ☐☐ 0. None		
Number of trips you have made within Iran in the past 10 years (Pilgrimage or non-Pilgrimage, and at least 100 kilometers away from your home) ___ ___ 0. None		
**Occupational Status**		
Are you currently occupied:	Yes: Job title______________________<br>No	
Choose occupation category:<br>(Based on the ISCO-88 Major, sub-major, and minor groups—3 digit classification)		
What is your primary source of income?<br>1. Insurance 2. Self 3. Spouse 4. Child/Children 5. Welfare, Charity 6. Parents 7. Other_____		

List all job positions held for at least one year, starting with the first job. If you had multiple jobs at the same time, list all of them. In order to list a job, it is necessary that you at least spend 8 hours per week at that job. Any work done at home (housewife, or carpet weaving) should also be included.

From (age)	To (age)	Job title	Job category (ISCO Code)

Home Location and Type of Fuel

List all places of residence (birth-present), in which you have at least lived one year:

From (age)	To (age)	Province	City	Village	Type of House	Type of Fuel Used for Heating	Type of Fuel Used for Cooking	Heating System

Type of House Codes:

1. Bricks and Steel
2. Wood and Bricks
3. Cement/Concrete
4. Stone and chalk/charcoal
5. Other__________

Types of Fuel Codes:

1. Oil/Gasoline
2. Wood, Firewood
3. Animal Waste
4. Gas
5. Electricity
6. Other___________

Heating System Codes:

1. Electrical Heater
2. Heater with a chimney
3. Fire place
4. Heater without chimney
5. Gas Burner
6. Other___________

Life Style

What is your primary source of your drinking water?
1. Well water 2. River water 3. Spring water 4. Tap water 5. Mineral water 6. Water Tank 7. Underground water Cistern 8. Other______________

Have you always used tap water? 1. Yes 2. No

How many years have you been using tap water? (if at all) _____________________

What was your primary source of drinking water before tap water?
1. Well water   2. River water   3. Spring water   4. Mineral water   5. Tanker water
6. Cistern   7. Other

Was this water source approved for use by the health department?
1. Yes   2. No

Type of kitchen: ☐ 1. Closed kitchen inside the house
☐ 2. Open kitchen inside the house   ☐ 3. Outside of the house

Do you use a range hood/kitchen ventilator?
☐ 1. Always   ☐ 2. Sometimes   ☐ 3. Don't have one

Does the kitchen have a window?
☐ 1. It does   ☐ 2. It does not

Is it open when you are cooking?
☐ 1. Yes   ☐ 2. No

Have you ever had contact with animals? If yes, complete the following: 1. Yes   2. No

From (age)	To (age)	Degree of Contact	Type of Animal(s)

Contact Codes:
1. Sometimes (keeping animals 200 meters away from home or workplace)
2. At least one time every two weeks, but not every day
3. Daily
4. Close daily contact (feeding or cleaning animal housing)

Animal codes:
1. Horse, donkey, camel
2. Sheep, cow, goat
3. Dog
4. birds

Reproductive History (women only)

Have you ever had a menstrual cycle?
1. Yes   2. No   3. Don't Know
2. Age (in years) at menarche—if don't know, enter 88, if never had the menstrual cycle, enter 89

Are you currently pregnant?
1. Yes   2. No

Number of pregnancies ☐☐

Number of live pregnancies ☐☐

History of still birth? ☐ 1. Yes   ☐ 2. No

Mother's age at first live birth: ☐☐

Mother's age at first pregnancy : ☐☐

Number of Abortions: ☐☐

Age at first Abortion: ☐☐

Total lactation time (in months) ☐☐

History of oophorectomy ☐ 1. One side ☐ 2. Both sides ☐ 2. No history

Age at the time of oophorectomy (in years) ___ ___

History of Tubectomy:
1. Yes 2. No

History of hysterectomy: ☐1. Yes—age at the time of hysterectomy (in years) ☐☐
☐2. No history

History of infertility ☐1. Yes ☐2. No

History of infertility medication use: ☐1. Yes ☐2. No

Have you ever used contraceptives (OCP, DMPA , etc)? If yes, complete the following:
1. Yes 2. No

**From (age)**	**To (age)**	**Type of Contraceptive**	**Length of Use**

Type of Contraceptive:
1. Pills
2. Implants
3. Long-term progesterone Injection
4. Intrauterine Devices (IUD)

Have you reached menopause? 1. Yes 2. No
If yes, age at menopause:____________

Did menopause naturally? ☐ 1. Yes ☐ 2. No

Do you/Have you used hormone replacement therapy? If yes, complete the following:
1. Yes 2. No 3. I don't know

**From (age)**	**To (age)**	**Type**	**Length of Use**

Do you have a history of breast or uterine cancer screening? If yes, complete the following:
1. Yes 2. No 3. I don't know

	1. Yes<br>2. No	If Yes, age at last exam	Number of times examed
Breast Exam (by Dr)			
Mamography			
Pap Smear			

Chronic Disease	1. Yes 2. No	Age at Diagnosis	Treated?
Diabetes			
Hypertension			
Ischemic Heart Disease (Angina, heart failure)			
Myocardial Infarction (MI)			
Cerebrovascular Accident			
Renal Failure			
Fatty liver (diagnosed by Dr)			
Hepatitis B			
Hepatitis C			
Chronic Respiratory Disorders (Asthma, Tuberculosis)			
Thyroid Disorders			
Kidney Stones			
Gallbladder Stones			
Rheumatoid Disorders			
Skin Cancer			
Breast Cancer			
Stomach Cancer			
Colorectal Cancer			

PERSIAN Cohort

Bladder Cancer			
Blood Cancers			
Esophageal Cancer			
Prostate Cancer			
Lung Cancer			
Cancer of the Central Nervous System			
Epilepsy			
Chronic recurrent headaches			
Depression (diagnosed by physician)			
Any other psychiatric disorder			
Learning disorders interfering with educational progress			
Memory problems, impairing your activities of daily living			
Cancer of the Larynx			
Cancer of the Tongue			
Uterine / Cervical Cancer			
Ovarian Cancer			
Lupus			
Multiple Sclerosis			
Gestational Diabetes			
Gestational Hypertension			

PERSIAN Cohort

Medical History Part 2		
**Question**	**1. Yes<br>2. No**	**Description**
Do you feel an irritation in your chest as you walk fast, or walk uphill (burning sensation, sternal irritation)?		
Have you ever had any swelling and edema in your body especially feet?		
Have you ever had a change in your urine color? Especially blood in urine at night?		
Have you ever had a burning sensation while urinating, polyuria, nocturia, or a history of wetting the bed?		
Have you ever had an abnormal urinalysis? (blood, protein, leukycytes, etc in urine)		
In the past year, have you experienced a burning sensation underneath your sternum?		
In the past year, have you experienced food regurgitation?		
Have you ever been officially diagnosed as having acid reflux from your stomach to your esophagus?		
In the past year, have you experienced bloating and distension of your abdomen, especially after eating food?		1. Almost daily<br>2. Several times / week<br>3. Several times / month<br>4. Every once in a while
How often do you move your bowels?<br>1. ________times per day<br>2. ________times per week<br>3. ________times per month		

Have you ever had fresh blood in your stool?		
Have you ever had unintentional weight loss (more than 5kg in one month, without dieting)		
Have you ever experienced yellowing of the sclera or skin during your lifetime?		Age: ---------
In the past year, have you experienced a cough lasting for at least two weeks?		Type:<br>1. With Sputum<br>2. Dry
In the past year, have you experienced shortness of breath along with wheezing last for at least two weeks?		
Have you ever experienced any problems when walking, such as losing balance that has lasted more than one week?		
Have you had a short-term fainting or syncope episode occurring for no specific reason more than one time in your lifetime?		
Have you ever had a difficulty/disruption in thought, memory or speaking that lasted more than one week?		
Have you ever had an impaired vision, or double vision that has lasted more than one week, and resolved on its own?		
Have you ever had muscle weakness in any part of your body tat has lasted more than one week?		
Have you ever experienced impairment in movement, tremors in hands and feet, lasting more than one week?		
Have you ever lost sensation, or felt a tingling sensation in your feet, lasting more than one week?		

Have you ever had a blow to the head, resulting in loss of consciousness (even if the LOC lasted for a short period of time)?		
Have you ever experienced recurrent headaches (at least twice) lasting more than 4 hours each time?		
Have you ever experienced dizziness that has greatly impacted your activities of daily living?		
Have you ever had a continuous wheezing sound in your ear, lasting more than one week?		
Have you had a broken bone in the past 5 years?		Location:___________ ____________________
At what age did you last have a broken bone?		Age: _______________
Did your last bone break occur as a result of falling?		
How many times have you fallen in the past year?		Number of times:____________
Have you ever had a pelvic or femur fracture?		
Have you ever been diagnosed with osteoporosis, or have been told by a physician that you are at risk for developing it?		
Have you ever had a back pain last more than one week, in a way that it disrupted your activities of daily living?		
Have you ever experienced back pain or stiffness in the morning, lasting more than one hour?		
Have you ever had joint pain?		

Have you ever experienced joint pain or stiffness in the morning, lasting more than one hour?		
Have you experienced recurrent oral aphthous?		
Have you experienced recurrent genital aphthous?		
Have you ever been diagnosed with rheumatoid arthritis by a physician?		
Have you ever had any surgeries?		Number of times:
Have you ever been hospitalized?		Number of times:
Have you ever received a blood transfusion?		Number of times:

Medications

Enter the following information for any medications taken

**Name**	**Use**<br>1. **Daily**<br>2. **Weekly**<br>3. **Monthly**	**Number of Times Used**	**Length of Use**

Family Medical History

**Disorder**	1. **Yes**<br>2. **No**	**Family Relationship**	
Diabetes			
Hypertension			

Ischemic Heart Disease			
Myocardial Infarction			
Cerebrovascular Accident			
Stomach Cancer			
Colorectal Cancer			
Breast Cancer			
Prostate Cancer			
Skin Cancer			
Bladder Cancer			
Blood Cancers			
Esophageal Cancer			
Lung Cancer			
Cancer of the Central Nervous System			
Epilepsy			
Psychiatric Disorders			
Chronic recurrent headaches			
Alzheimer's Disease			
Pelvic or Femur Fracture			
Cancer of the Larynx			

Cancer of the tongue			
Uterine/Cervical Cancer			
Ovarian Cancer			
Lupus			
Multiple Sclerosis			

Relationship Codes:

1. Father **2. Mother** **3. Brother** **4. Sister** **5. Step-Brother**
6. Step-Sister **7. Son** **8. Daughter** **9. Grandparents** **10. Spouse**

Oral and Dental Health

Number of times you brush your teeth:
1. Once Daily 2. Twice Daily 3. Three times/day
4. Other: ____________ 5. I don't brush my teeth

DMF: Total number of teeth:
Number of decayed teeth:
Number of missing teeth:
Number of fillings:

Are there any lesions seen inside the mouth? (Examined)

Do you floss your teeth?	1. Yes<br>2. No	If yes, Times per week:<br>______________________
Do you have dentures?	1. Yes<br>2. No	If yes, age at which you started using dentures: _____

Number of times you brush your dentures:
1. Once Daily 2. Twice Daily 3. Three times/day
4. Other: ____________ 5. I don't brush my dentures

Do you use mouthwash?	1. Yes<br>2. No	If yes, Times per week:<br>______________________

Blood Pressure and Pulse Measurements

Blood Pressure and pulse should be measure twice

Blood Pressure:
- Right Arm:

1st Measurement: Systolic/Diastolic: |__| |__| |__|/ |__| |__| |__|
2nd Measurement: Systolic/Diastolic: |__| |__| |__|/ |__| |__| |__|

- Left Arm:

1st Measurement: Systolic/Diastolic |__| |__| |__|/ |__| |__| |__|

2nd Measurement: Systolic/Diastolic: |__| |__| |__|/ |__| |__| |__|

Pulse Rate:

1st Resting Heart Rate/ minute |__| |__| |__|

2nd Resting Heart Rate/minute |__| |__| |__|

Physical Assessment and Disabilities

<table>
<tr><td>Individual has baldness</td><td colspan="2">1. Yes
2. No</td></tr>
<tr><td colspan="3">If yes, type of baldness pattern (choose from pictures)
Male Patterns: Female Patterns:
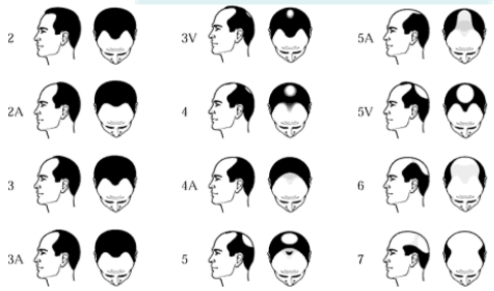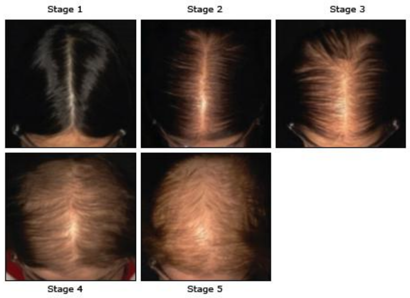</td></tr>
<tr><td>Presence of facial hair (in Women)</td><td colspan="2">1. Yes
2. No</td></tr>
<tr><td>Eye Color</td><td colspan="2">1. Brown/Dark Brown
2. Hazel
3. Green
4. Blue/Grey</td></tr>
<tr><td>Presence of Physical/Sensational Disability or Amputations:</td><td>1. Yes
2. No</td><td>If Yes, Reason:
1. Congenital
2. War Injury
3. Occupational/Accidental
4. Due to disease (Diabetes)
5. Polio
body part involved:
1. Right hand
2. Left hand
3. Right leg
4. Left leg
5. Finger/s
6. Toe/s
7. Eye
8. Ear</td></tr>
</table>

Spinal Cord Abnormalities	1. Yes<br>2. No	If Yes, type of Abnormality:<br>1. Scoliosis<br>2. Lordosis<br>3. Kyphosis

Anthropometric Measurements

Height and weight should be measured without shoes and in light clothing

Height in centimeters (cm)	|__||__||__|
Weight in kilograms (kg)	|__||__||__|
Waist circumference (cm)	|__||__||__|
Hip circumference (cm)	|__||__||__|
Wrist circumference (cm)	|__||__||__|

Physical Activity

**Physical Activity (All questions refer to the amount of time spent doing an activity in a typical 24 hour day)**	**Pattern 1: ___ Months / Year**		**Pattern 2: ___ Months / Year**	
	**Hours**	**Minute**	**Hours**	**Minute**
Sleeping at Night	__ __ :	__ __	__ __ :	__ __
Napping during the day	__ __ :	__ __	__ __ :	__ __
Lying in bed awake(for more than 10 minutes per day)	__ __ :	__ __	__ __ :	__ __
Watching TV, listening to music, Watching movies , etc.	__ __ :	__ __	__ __ :	__ __
Reading newspaper, books, magazines, etc.	__ __ :	__ __	__ __ :	__ __
Working at a table	__ __ :	__ __	__ __ :	__ __
Sitting at a table to eat, at meetings, etc.	__ __ :	__ __	__ __ :	__ __
Cooking, washing dishes, activities done standing	__ __ :	__ __	__ __ :	__ __
Driving, or other technical work that is done sitting down	__ __ :	__ __	__ __ :	__ __
Cleaning the house (laundry, dusting, vacuuming)	__ __ :	__ __	__ __ :	__ __
Selling goods (standing on foot in a store)	__ __ :	__ __	__ __ :	__ __
Taking a walk, light exercise, light dance	__ __ :	__ __	__ __ :	__ __
Speed walking, light aerobic exercise, riding a bicycle (for fun or to go to work)	__ __ :	__ __	__ __ :	__ __
Driving heavy machinery	__ __ :	__ __	__ __ :	__ __
Construction work (painting, molding, wood work, etc.)	__ __ :	__ __	__ __ :	__ __
Moving equipment, taking them up or down stairs, etc.	__ __ :	__ __	__ __ :	__ __

PERSIAN Cohort

Heavy mechanical work, smithing, carpentry, etc.	__ __ : __ __	__ __ : __ __
Heavy labor or field work	__ __ : __ __	__ __ : __ __
Body building, heavy aerobic, similar activities	__ __ : __ __	__ __ : __ __
**Total (Must add up to 24 hours)**	__ __ : __ __	__ __ : __ __

Average time spent per week, doing sports

Type of Sport	Hours	Minute
	__ __ : __ __	
	__ __ : __ __	

Sleep and Circadian Rhythm

What time do you usually fall asleep? __ __:__ __

On average, how long after you go to be do you actually fall asleep? __ __:__ __

What time do you usually wake up in the morning? __ __:__ __

What time would you like to be able to wake up in the mornings? __ __:__ __

Do you also take a day-time nap (more than three times per week)?
1. Yes, on average __________ minutes/nap 2. No

In the past year, have you had a night shift at work (defined as at least 6 hours between 9pm and 6 am)?
1. Yes, _______ times/month 2. No

Do you move your legs a lot in your sleep, waking up someone sleeping next to you?
1. Yes 2. No 3. I don't know

Do you fall asleep unintentionally during the day, at times when you don't have much to do? 1. Yes 2. No

Do you use sleeping pills on a regular basis (more than 2 times per week)
1. Yes 2. No

Personal Habits Drug and Alcohol Use)

Have you smoked at least 100 cigarettes in your life time?
1. Yes 2. No (Skip to ***) 3. I don't know

At what age did you smoke your first cigarette? ________ years old

At what age did you start smoking regularly? _________ years old

Do you currently smoke?
1. Yes, daily 2. Yes, occasionally 3. No

On average, how many times did/do you smoke in 24 hours? _________

At what age did you stop smoking daily? ________ years old

Please indicate your cigarette use since you started:

Days/Week	Number/day	Type (Use below)	From (Age)	To (Age)

Cigarette Type Codes: 1. Manufactured cigarettes 2. Hand-Made Cigarette 3. Cigar (Leaf)

***Are/ Were you exposed to smoke from a cigarette in your house (Second hand smoking)? 1. Yes, _______________Hours/day 2. No

How many hours per day are you exposed to cigarette smoke at your work?
1. I don't work outside the house 2. Almost never 3. __ __:__ __ hours /day

Growing up, did someone in your house smoke?
1. Yes 2. No

Have you ever used Naas, Hookah, pipe, or chopogh?
1. Yes (complete below) 2. No

Type	From (age)	To (Age)	Times/Day	Days/Week
Naas				
Chopogh				
Pipe				
Hookah				

Have you ever used ilicit drugs?
1. Yes (Complete below) 2. No

Type	Method	From (age)	To (Age)	Times/Day	Days/Week
Opium					
Heroin					
Sookhteh					
Shireh					
Shisheh					
Cocaine					
Crack					
Crystal					
Pan (Pan Parag)					
Others					

Method Codes: 1. Oral 2. Inhalation 3. Injection

Have you ever used alcoholic beverages?
1. Yes (Complete below) 2. No

Type	From (Age)	To (Age)	Average Amount Used each Time (cc)		Times/Month
Beer					
Drinks with >40% Alcohol (Vodka, Gin, Whisky, etc)					

PERSIAN Cohort

Home Made Drinks					
Others					

Food Frequency Questionnaire

Food Item	Portion Size	Number of Times Used per: Day	Week	Month	Year	Amount Used Each Time (Based on Portion Size)	Months in a Year	Comments
**Breads and Cereals**								
Lavash Bread	Palm of Hand/ Complete Bread							
Barbari / Taftoon	Palm of Hand/ Complete Bread							
Sangak	Palm of Hand/ Complete Bread							
Baguette	One Individual Size							1. White<br>2. Whole Wheat
Cooked Rice	One Skimmer							
Cooked Noodles	One Skimmer							
Cooked Oats	Table Spoon							
**Legumes (Measurements based on cooked amount)**								
Beans	Table Spoon							
Chickpeas	Table Spoon							
Lentils	Table Spoon							
Soy	Table Spoon							
Fava Beans	Table Spoon							
**Meats, Proteins, and Meat Products**								
Red Meat	Match Box (30g)							1. Low Fat<br>2. High Fat
Chicken	Match Box (30g)							1. With Skin<br>2. Skinless
Chicken Organs	Match Box (30g)							1. Fried<br>2. Boiled<br>3. Grilled
Eggs	One							
Fish	Cassette Size							

Tuna	Table Spoon							1. With Oil<br>2. Without Oil
Sausage / Salami	Use Album							
Hamburger	One							
Bovine Organs	Match Box (30g)							1. Fried<br>2. Boiled<br>3. Grilled
Sheep Brain	One							
Sheep Tongue	One							
Kaleh Pacheh	Bowl							
Pizza	Slice							
**Dairy**								
Milk	Cup							1. Low Fat<br>2. High Fat / Traditional
Yogurt	Cup							1. Low Fat<br>2. High Fat / Traditional<br>3. Creamy<br>4. Extracted
Cheese	Match Box (30g)							1. Regular<br>2. Creamy<br>3. Traditional
Doogh (Yogurt Drink)	Cup							
Kashk	Table Spoon							
Flavored Milk	Cup							
**Vegetables**								
Lettuce	Cup							
Cabbage	Cup							
Tomatoes	One Average Sized							
Cucumber	One Average Sized							
Raw Greens	Cup							
Cooked Greens	Table Spoon							
Eggplant	One Average Sized							
Celery	Cup							
Beets	One Average							

PERSIAN Cohort

	Sized							
Potatoes	One Average Sized							
carrots	One Average Sized							1. Raw<br>2. Cooked<br>3. Fried<br>4. Carrot Juice
Garlic	One Clove							
Onions	One Average Sized							1. Fried<br>2. Cooked<br>3. Raw
Bell Peppers	Cup							
Mushrooms	Table Spoon							
Corn	Cup							
Green Peas	Table Spoon							
Green Beans	Table Spoon							
Squash	One Average Sized							
Green Peppers (Hot or Normal)	One Average Sized							
**Fruits**								
Cantaloupe	One Cut (Album)							
Honeydew	One Cut (Album)							
Watermelon	One Cut (Album)							
Apricot	One Average Sized							
Cherry / Sour Cherry	Fruit Plate							
Peach / Nectarine	One Average Sized							
Green Tomatoes	One Average Sized							
Berries	Fruit Plate							
Strawberries	One Average Sized							
Plums	One Average Sized							
Fig	One Average Sized							
Grapes	Use Album							

PERSIAN Cohort

Pear	One Average Sized							
Apples	One Average Sized							1. With Skin<br>2. Skinless
Kiwi	One Average Sized							
Citrus Fruits	One Average Sized							
Pomegranate	One Average Sized							
Banana	One Average Sized							
Persimmon	One Average Sized							
Dates	One Average Sized							
Natural Fruit Juice	Cup							1. Apple<br>2. Orange<br>3. Cantaloupe<br>4. Others
Dried Figs, Plums, etc.	One							
Raisins / Dried Berries	Table Spoon							
Canned Fruit	Cup							
**Fats, Oils, Nuts**								
Vegetable Butter, Margarine	Tea Spoon							
Butter	Tea Spoon							
Solid, Semi-Solid Fats	Table Spoon							1. Vegetable<br>2. Animal<br>3. Donbeh
Oils	Table Spoon							1. Canola, Soya<br>2. Others<br>3. Frying Oil
Olive Oil	Table Spoon							
Olives	One							
Mayonnaise, Salad Dressing	Table Spoon							
Walnuts	One							
Peanuts	One							
Others Nuts	One							
Seeds	Table Spoon							
Cream	Tea Spoon							

PERSIAN Cohort

**Sugar and Sweeteners**								
Sugar Cubes	One Cube							
Sweeteners (Rock Candy, Noghl, Candy)	One Cube							
Honey	Tea Spoon							
Jam	Tea Spoon							
Sugar	Coffee Spoon							
**Miscellaneous**								
Tea	Cup							1. Light Colored<br>2. Regular<br>3. Dark
Soda	Cup							1. Black<br>2. Orange<br>3. White
Non-Alcoholic Beer	Cup							
Coffee / Nescafe	Cup							
Ice Cream	One / Cup							
Dry Pastries / Cakes	One							
Creamy Pastries / Cakes	One							
Chocolate	One							
Chips	Individual Sized Pack							
Cheese Puffs	Individual Sized Pack							
Halva	Table Spoon							
Pickles	One							
Torshi	½ Cup							
Tomato Paste	Table Spoon							
Juice from Concentrate	Cup							
Pickled Vegetables	½ Cup							
Crackers / Wafers	One							

PERSIAN Cohort

Other Pastes (Pomegranate, etc.)	Table Spoon							
**Spices**								
Salt	Coffee Spoon							

Supplements

Type	Number Consumed per:			
	Day	Week	Month	Year
Multivitamin with Minerals				
Multivitamin (Without Minerals)				
Calcium + D				
Calcium				
Vitamin D (Pills)				
Vitamin D Injection				
Folic Acid				
Omega 3 / Fish Oil				
Iron				
Zinc				
Others				

Drinking Water

Water Drank in Different Seasons	Portion Size	Daily	Weekly	Monthly
Average amount in Summer				
Average amount in other Seasons				

Cooking, Food Storage and Dietary Habits

How many times / day do you eat?

1. 3 meals (breakfast, lunch, and dinner)
2. 4 times (breakfast, lunch, dinner, snack)
3. 5-6 times (3 meals and 2-3 snacks)
4. More than 6 times
5. Less than 3 meals

Do you have a habit of adding salt to your food at the table?

1. Yes 2. Sometimes 3. No

The foods you consume are usually:

1. Low Salt 2. Average 3. Salty

How often do you eat grilled foods?

1. Never
2. 1-3 time/ Week
3. 1-3 time/ Month
4. Less than once/month
5. Daily

How often do you eat fried food?

1. Never
2. 1-3 time/ Week
3. 1-3 time/ Month
4. Less than once/month
5. Daily

To what degree do you fry vegetables such as potatoes, eggplant or squash?

1. Sauté
2. Until it is golden brown
3. Until it is dark brown/burnt

To what degree o you fry onions?

1. Sauté
2. Until it is golden brown
3. Until it is dark brown/burnt

To what degree do you fry herbs?

1. Sauté
2. Until it is golden brown
3. Until it is dark brown/burnt

What type of fat/oil do you use for frying food?

1. Solid fats
2. Semi-solid fat
3. Liquid oil
4. Frying oil
5. Other

Do you re-use the oil after you've used it to fry or cook other foods?

1. Yes 2. No

If yes, up to how many times do you re-use oil?

______________ times type of oil used__________________________

If a food, such as jam, pickles, tomato paste, lemon juice or vinegar has molded, do you throw it out entirely, or do you remove the molded area and use the rest?

1. Use the remaining sections
2. Throw it all out

Do you use smoked foods? (Smoked fish, smoked rice)

1. Never
2. 1-3 time/ Week
3. 1-3 time/ Month
4. Less than once/month
5. Daily

How do you store vegetables?

1. Dried
2. Refrigerated
3. Frozen

If you keep vegetables in the refrigerator or freezer, how do you keep them:

1. Raw
2. Boiled
3. fried

How do you keep meat and meat products in the refrigerator or freezer?

1. Raw
2. Boiled
3. fried

At what temperature do you drink tea or coffee?

1. Very hot, immediately after pouring it
2. Hot
3. Lukewarm
4. Cold

At what temperature do you consume soups or other liquid foods?

1. Very hot, immediately after pouring it
2. Hot
3. Lukewarm
4. Cold

What type of container do you use to store water?

1. Plastic
2. Steel
3. Chinaware
4. Glass
5. Other

How do you store the following food items? (choose 2)

1. Bread
2. Juice/Sour grape Juice
3. Tomato juice/Tomato paste
4. Pickles and pickled vegetables

Codes: 1. Plastic 2. Steel 3. Wrapped in Cloth 4. Glass 5. Other

What types of pots and pans do you use for cooking? (Choose 2-3)

1. Zinc 2. Copper 3. Aluminum 4. Glazed 5. Teflon
6. Cast Iron 7. Steel 8. Pyrex 9. Other

Do you use Teflon pots/pans that have scratches on them?

1. Yes 2. No

In what type of dishes do you eat food? (choose 2-3)

1. China 2. Aluminum 3. Glazed 4. Melamine 5. Steel
6. Plastic 7. Glass 8. Arcopal

In what type of container do you keep left overs? (choose 2-3)

1. Copper 2. China 3. Aluminum 4. Glazed 5. Melamine
6. Steel 7. Plastic 8. Glass

Please list any food allergies:____________________________________

List all spices that you use on a regular basis: ______________________________

Do you use herbal teas or herbal powders?

1. Yes 2. No

If you answered yes to the previous question, what type of herb, and for what reason?

____

PERSIAN Cohort

Mobile Use

For how many years have you been using mobile phones? (Please indicate **zero** if you do not use mobile phones.) ____ ____ Years

During the last 12 month, what has been the **average length** of time you spent **making and receiving calls?** You can answer in minutes or hours per a typical day, week or month (Please indicate **zero** if you do not use mobile phones). You can give me a range, if that's easier.

__________ Minutes	1. Per day
Or	2. Per Week
__________ Hours	3.Per Month

During the last 12 month, what has been the **average length** of time you spent **doing tasks other than making calls (e.g. texting, chatting, gaming, internet browsing)?** You can answer in minutes or hours per a typical day, week or month (Please indicate **zero** if you do not use mobile phones for other tasks than making calls). You can give me a range, if that's easier.

__________ Minutes	1. Per day
Or	2. Per Week
__________ Hours	3.Per Month

Pesticide Use

Is your home near an area where there is agricultural activity (growing fields)?

1. No
2. Yes
   - If yes, how far away?
     1. Distant >200 meters
     2. Quite close (100-200m)
     3. Close (50-100m)
     4. Very close (<50m)

During the last 12 months, have you come into contact with pesticides by:	**1. Yes**<br>**2. No**
a. applying pesticides at farms, green houses, or agricultural fields	
b. applying pesticides at home to your plants	
c. applying insecticides at home (e.g. to kill mosquitoes, flies, ants, cockroaches, etc.)	
d. mixing and loading pesticides/insecticides	
e. entering work areas where pesticides were just applied to perform any duties	

PERSIAN Cohort

f. cleaning equipment contaminated with pesticides (spray equipment, PPE, pesticide containers, storage areas)	
g. repairing or using equipment that has recently been used to apply pesticides	
h. handling stored pesticide products	
i. directing spray operations	

If you have answered "Yes" to any of the activities in question 9, we want to know the **number of times** (**during the last 12 months**), and the **length of each time** (**minutes**) have you do that activity and whether you use personal protective equipments (PPE) such as coveralls or protective suits, footwear, gloves, aprons, respirators, eyewear, and headgear when you are doing that activity.

**Activity**	**Times**	**Length (Minutes)**	**Using PPE**<br>**1. Yes**<br>**2. No**
a. applying pesticides at farms, green houses, or agricultural fields			
b. applying pesticides at home to your plants			
c. applying insecticides at home (e.g. to kill mosquitoes, flies, ants, cockroaches, etc.)			
d. mixing and loading pesticides/insecticides			
e. entering work areas where pesticides were just applied to perform any duties			
f. cleaning equipment contaminated with pesticides (spray equipment, PPE, pesticide containers, storage areas)			
g. repairing or using equipment that has recently been used to apply pesticides			
h. handling stored pesticide products			
i. directing spray operations			

PERSIAN Cohort
