# Supplementary material for: Cohort profile: Bandar Kong prospective study of chronic non-communicable diseases
Source: PLoS One. 2022 May 12;17(5):e0265388. doi: 10.1371/journal.pone.0265388 (PMC9098057; doi:10.1371/journal.pone.0265388)
Supplement: S3 File — (PDF) [file pone.0265388.s003.pdf]

American Journal of Epidemiology
© The Author(s) 2018. Published by Oxford University Press on behalf of the Johns Hopkins Bloomberg School of Public Health. All rights reserved. For permissions, please e-mail: journals.permissions@oup.com.
DOI: 10.1093/aje/kwx314

Study Design

Prospective Epidemiological Research Studies in Iran (the PERSIAN Cohort Study): Rationale, Objectives, and Design

Hossein Poustchi, Sareh Eghtesad, Farin Kamangar, Arash Etemadi, Abbas-Ali Keshtkar, Azita Hekmatdoost, Zahra Mohammadi, Zahra Mahmoudi, Amaneh Shayanrad, Farzin Roozafzai, Mahdi Sheikh, Alireza Jalaeikhoo, Mohammad Hossein Somi, Fariborz Mansour-Ghanaei, Farid Najafi, Ehsan Bahramali, Amirhoushang Mehrparvar, Alireza Ansari-Moghaddam, Ahmad Ali Enayati, Ali Esmaeili Nadimi, Abbas Rezaianzadeh, Nader Saki, Fatemeh Alipour, Roya Kelishadi, Afarin Rahimi-Movaghar, Nayyereh Aminisani, Paolo Boffetta, and Reza Malekzadeh*

* Correspondence to Dr. Reza Malekzadeh, Digestive Diseases Research Institute, Tehran University of Medical Sciences, Shariati Hospital, North Kargar Street, Tehran 1411713135, Iran (e-mail: malek@tums.ac.ir).

Initially submitted December 5, 2016; accepted for publication August 30, 2017.

Noncommunicable diseases (NCDs) account for 76% of deaths in Iran, and this number is on the rise, in parallel with global rates. Many risk factors associated with NCDs are preventable; however, it is first necessary to conduct observational studies to identify relevant risk factors and the most appropriate approach to controlling them. Iran is a multiethnic country; therefore, in 2014 the Ministry of Health and Medical Education launched a nationwide cohort study—Prospective Epidemiological Research Studies in Iran (PERSIAN)—in order to identify the most prevalent NCDs among Iran's ethnic groups and to investigate effective methods of prevention. The PERSIAN study consists of 4 population-based cohorts; the adult component (the PERSIAN Cohort Study), described in this article, is a prospective cohort study including 180,000 persons aged 35–70 years from 18 distinct areas of Iran. Upon joining the cohort, participants respond to interviewer-administered questionnaires. Blood, urine, hair, and nail samples are collected and stored. To ensure consistency, centrally purchased equipment is sent to all sites, and the same team trains all personnel. Routine visits and quality assurance/control measures are taken to ensure protocol adherence. Participants are followed for 15 years postenrollment. The PERSIAN study is currently in the enrollment phase; cohort profiles will soon emerge.

cohort studies; Iran; noncommunicable diseases; Prospective Epidemiological Research Studies in Iran (PERSIAN)

Abbreviations: CVD, cardiovascular diseases; GCS, Golestan Cohort Study; NCDs, noncommunicable diseases; PERSIAN, Prospective Epidemiological Research Studies in Iran; QA, quality assurance; QC, quality control.

Over the past 30 years, Iran's health-care system has mainly focused on controlling infectious diseases, malnutrition, and birth complications, as these conditions have ranked high in the country's top causes of death (1). After the infrastructure to contain those diseases was successfully established, studies indicated a change in disease patterns and causes of death in Iran—which, paralleling worldwide trends, showed a decrease in incidence of communicable diseases and an increase in the prevalence of noncommunicable diseases (NCDs) (1).

NCDs account for almost 76% of deaths in Iran (2), a figure larger than the global figure of 68% (3). Cardiovascular disease (CVD), the leading cause of death worldwide, accounts for 46% of global deaths and 51% of Iran's (3). Cumulative economic losses associated with the most common NCDs in low- and middle-income countries are projected to be over $7 trillion during the years 2011–2025, pushing millions of people below the poverty line (4). Because of the continuing increase in NCDs and their heavy burden, a change in Iran's health-care system is warranted to develop effective prevention and treatment strategies and to decrease the heavy economic burden of NCDs. For health-care policies to be successful, however, they need to be supported by strong evidence.

Downloaded from https://academic.oup.com/aje/advance-article-abstract/doi/10.1093/aje/kwx314/4627678
by Tehran University of Medical Science user
on 24 February 2018

Cohort studies have greatly contributed to the evidence needed to identify risk factors for diseases and disease etiology. Since most NCDs take decades to progress, exposures encountered during that period are substantial to their development. Therefore, only longitudinal surveillance and careful recording of those exposures can help in the identification of possible risk factors and causes (5). For this reason, cohort studies are known as one of the most effective investigative strategies in etiological epidemiology.

Since the start of the Framingham Heart Study, the first modern population-based cohort study, in 1948, this type of research has gained widespread acceptance in developed countries. In developing countries, however, cohort studies are scarce. Many of the results from cohort studies conducted in developed countries can be applied to developing countries such as Iran; however, because of population diversity and differences in genetics, social and environmental exposures, lifestyles, and other factors that can influence disease development, a cohort study focused on the Iranian lifestyle can provide more relevant evidence for Iranian health-care policies. The Golestan Cohort Study (GCS), which includes 50,000 persons in northern Iran, has thus far significantly contributed to knowledge on risk factors associated with gastrointestinal cancer and other conditions, including CVD (6–8). Due to the diverse ethnic population of Iran, however, a study including persons of many ethnicities and cultures was needed. Therefore, in 2013, the Iranian Ministry of Health and Medical Education sought to establish a nationwide cohort study—Prospective Epidemiological Research Studies in Iran (PERSIAN)—to create the framework needed to modify health-care policies while, at the same time, encouraging research at Iranian medical universities by empowering them with the necessary resources to take part in the study. In this article, we briefly provide an overview of the PERSIAN cohort and then focus on the objectives and design of the study.

OVERVIEW OF THE PERSIAN COHORT STUDY

The PERSIAN study started in 2014. Although “PERSIAN Cohort Study” refers to the main adult component of the study, the cohort actually encompasses 3 other components as well, named the PERSIAN Birth Cohort, the PERSIAN Youth Cohort, and the PERSIAN Elderly Cohort; these studies are simultaneously being carried out in different parts of Iran. Details on these cohorts are provided in Table 1. Enrollment has started in all of the study components, and each component is executed and monitored by one designated central committee, to ensure consistent data collection at all sites. Methodology for the PERSIAN Birth, Youth, and Elderly cohorts will be explained elsewhere in future publications. The main and most extensive component, the adult component—which has also started enrollment—is explained in detail below.

OBJECTIVES AND OUTCOMES OF INTEREST

The objectives of the PERSIAN Cohort Study are divided into 2 categories, as follows.

Research and public health objectives

1. To determine cumulative incidence/incidence rates of NCDs or other outcomes.
2. To determine all-cause and cause-specific mortality rates for the leading causes of death.
3. To implement full cohort, nested case-control, and case-control studies of risk and protective factors for NCDs.
4. To compare the relationships between risk factors and NCDs among different Iranian ethnic groups at different cohort sites.
5. To build a population-based research infrastructure for medical universities.

Health system research objectives

1. To design/improve and optimize health information systems such as cancer registries, death registries, hospital information systems, etc.
2. To enhance national and international collaborations between medical universities and research institutes for joint medical and epidemiologic research projects and exchange of students.
3. To establish the largest biobank in Iran for basic and genetic scientific research and discovery of biomarkers and other biological determinants of disease.

Table 1. Characteristics of the PERSIAN Cohort Study Components, Iran, 2014–2017

Component	Sex of Target Population	Age Range, years	Sample Size (*n*)	No. of Cohort Sites	Main Goal
PERSIAN Cohort (main, adult)	Males and females	35–70	180,000	18	See “Objectives and Outcomes of Interest” section in text
PERSIAN Birth Cohort	Males and females	Periconception to 18	12,000	4	To study the influence of various biological and environmental factors on human development
PERSIAN Youth Cohort	Males and females	15–34	9,000	3	To find the prevalence of and risk factors associated with the most common mental health concerns, as well as substance use
PERSIAN Elderly Cohort	Males and females	>50	5,000	1	To study the different aspects of the aging process in order to improve quality of life

Abbreviation: PERSIAN, Prospective Epidemiological Research Studies in Iran.

Downloaded from https://academic.oup.com/aje/advance-article-abstract/doi/10.1093/aje/kwx314/4627678
by Tehran University of Medical Science user
on 24 February 2018

Outcomes of interest

The outcomes of interest in the PERSIAN Cohort Study include: 1) number of deaths, by cause; 2) incidence of major NCDs, such as CVD, cancer, metabolic disorders, digestive, respiratory, renal, and hepatic disorders, neurological diseases, etc.; and 3) trends in major risk factors associated with diseases, including anthropometric factors, physical activity, and many physiological, lifestyle, nutritional, and environmental factors.

METHODS

Design of the PERSIAN Cohort Study

The PERSIAN Cohort Study is a prospective study aiming to include 180,000 Iranians aged 35–70 years from 18 geographically distinct areas of Iran. While the Ministry of Health and Medical Education oversees the project, investigators at local universities carry it out. These include the universities of medical sciences in Ahvaz, Ardabil, Bandar Abbas, Fasa, Guilan, Kermanshah, Mashhad, Mazandaran, Rafsanjan, Sabzevar, Shahrekord, Shiraz (2 sites), Tabriz, Urmia, Yasuj, Yazd, and Zahedan. The design of the PERSIAN Cohort Study was approved by the ethics committees of the Ministry of Health and Medical Education, the Digestive Diseases Research Institute (Tehran University of Medical Sciences), and each participating university.

Site selection. The Iranian people comprise individuals of many ethnicities, including Fars (Persians), Turks/Azari, Kurds, Lurs, Balouch, Arabs, etc., with unique cultural practices and lifestyles, making their exposures different. Additionally, Iran has a unique climate with woodlands and forests in the north, hot, dry deserts in the center of the country, and semitropical regions in the south. Although the incidence of NCDs has increased throughout the country, Iran still shows strong regional differences in the risk of specific diseases (9–12). Therefore, PERSIAN was designed to represent all of the major ethnic groups living in different geographical areas of Iran, enabling it to capture a wide range of environmental diversity, lifestyle, and socioeconomic differences, as well as many other exposures influencing disease patterns that may be very unique to one area or highly prevalent in another. The PERSIAN Cohort sites are shown in Figure 1, and details about each site are given in Table 2. Cohort sites include both urban and rural areas and were selected on the basis of the following criteria: 1) inclusion of the major ethnic groups within Iran; 2) inclusion of a large geographical area encompassing different climates, in order to capture different environmental and occupational exposures; 3) inclusion of areas with low migration rates, in order to limit loss to follow-up; and 4) local disease patterns and exposures that had already been found in previous studies.

Participant selection. Men and women aged 35–70 years residing at the PERSIAN Cohort sites are invited to participate in the study. This age range was chosen for 3 reasons. First, persons in this age group are more likely to have well-established behaviors and lifestyles. Second, they are active and energetic enough to participate in a study; and third, they will, within a reasonable amount of time, incur the study's outcomes of interest. In the smaller cities/urban areas chosen for the cohort, all persons in this age range are included in the study. In larger cities with 1 predominant ethnic group, different districts of the city were chosen to include different socioeconomic status levels; however, in other cities with residents of more than 1 ethnicity, areas known to have better representation of the ethnic groups in mind were chosen.

Other inclusion criteria include being of Iranian descent and living in one of the designated areas for at least 9 months of the year. Persons with physical or psychological disabilities that render them unable to complete the enrollment process are excluded from the study.

Sample size. The PERSIAN Cohort Study is designed to investigate multiple outcomes and exposures. The overall sample size (n = 180,000) and site-specific sample sizes (n = 10,000) are large enough to detect a wide range of associations with adequate statistical power. For example, if the prevalence of an exposure in the population is 10%, at an α level of 0.05, 150 cases of the outcome of interest will give us 90% power to detect a relative risk of 2. To obtain 150 cases over a period of 5 years, the study needs only 30 cases of the outcome of interest per year—equivalent to approximately 18 cases per 100,000 person-years for the entire cohort or 300 per 100,000 person-years for each site-specific cohort. Given the age range of the cohort population, it is expected that the overall mortality rate, the CVD mortality rate, and the cancer mortality rate will be close to 1,000, 500, and 250 per 100,000 person-years, respectively (13–18). The incidence rate of several cancers will be higher than 18 per 100,000 person-years. It is hoped that follow-up will continue for 15 years, so that adequate power to detect associations with incidence rates less than 10 per 100,000 person-years is reached.

Recruitment in PERSIAN. Using regional records, a population census of each study site is generated. Based on the census, trained personnel go door to door to first confirm the presence of persons meeting the study's age requirement and then provide them with an explanation of the study, along with information pamphlets. The door-to-door method was chosen because a face-to-face invitation is more effective in the Iranian culture, improving participation. To further enhance participation, persons known to be trusted in the community, such as religious figures or government officials, are also spoken to; these people subsequently promote participation. If individuals agree to participate, they are later contacted by phone to set an appointment date, when they would have to go to their local cohort center. Recruitment and enrollment occur simultaneously and are expected to take about 24–30 months to complete.

Enrollment phase data- and sample-gathering. The work flow at the cohort centers is shown in Figure 2. Below we explain each step in the same order as shown in the flow chart.

Registration. Upon arrival at the cohort center, written informed consent is obtained from each individual, who is then registered using credible personal identification documents. A unique barcode is given to each individual, which is used to label all biological samples and documentations.

Laboratory procedures. Since enrollees are asked to arrive in a fasting state, the next step after registration is collection of biological samples. Twenty-five milliliters of blood is

Downloaded from https://academic.oup.com/aje/advance-article-abstract/doi/10.1093/aje/kwx314/4627678
by Tehran University of Medical Science user
on 24 February 2018

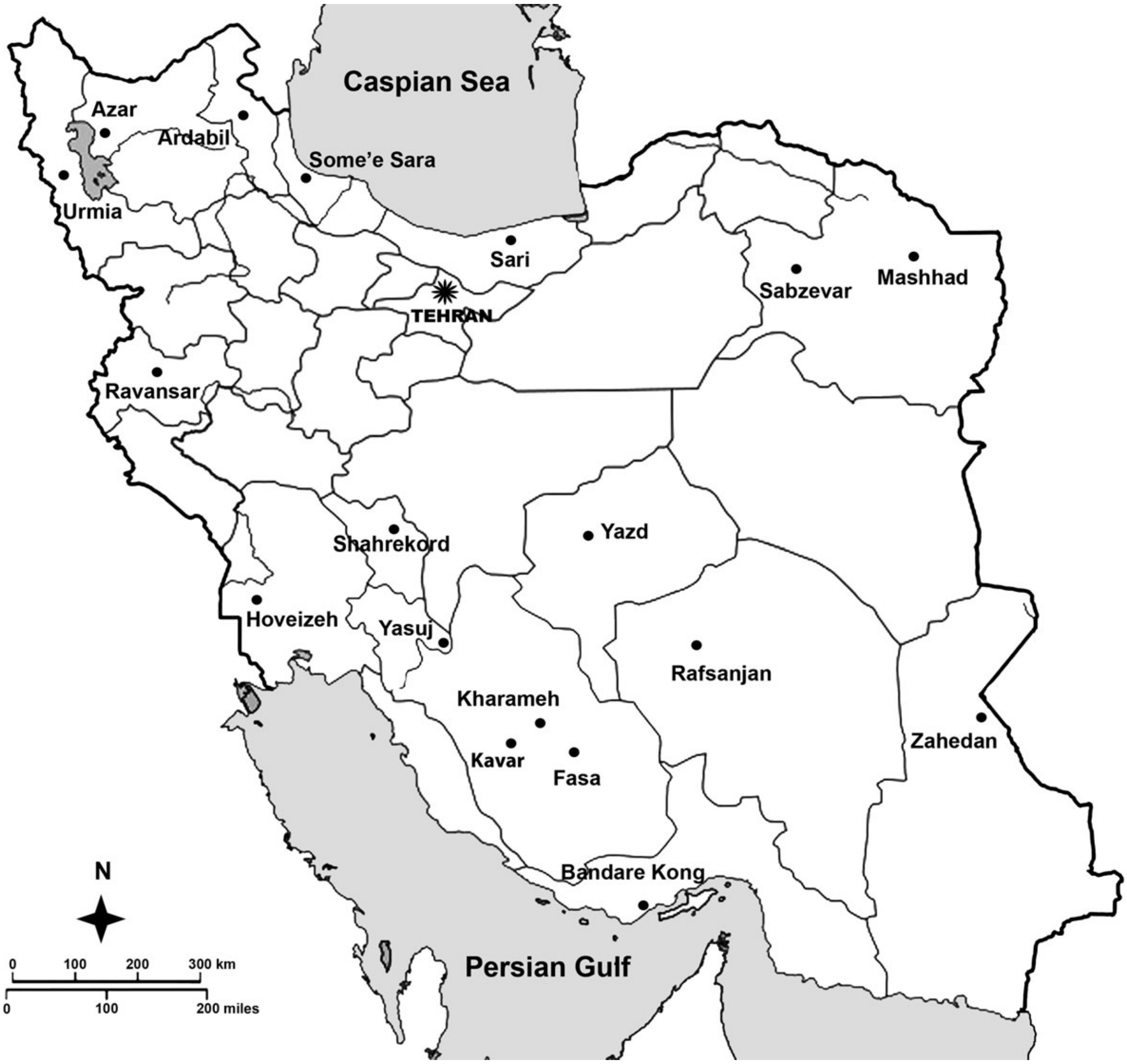

Figure 1. Locations of research centers in the PERSIAN Cohort Study, Iran, 2014–2017. The sites were chosen so that all of the major ethnic groups in Iran, as well as variable climates and geographical regions, are included. PERSIAN, Prospective Epidemiological Research Studies in Iran.

collected from each individual using Vacutainers (Greiner Bio-One International GmbH, Kremsmunster, Austria). Blood is centrifuged and fractioned into different aliquots as shown in Table 3. All aliquots are then labeled with participants' unique barcodes and stored in freezers (−70°C). In addition to storing the samples, a small amount of blood is used to conduct the following biochemistry tests: complete blood count, fasting blood sugar level, and concentrations of total cholesterol, high-density lipoprotein cholesterol, triglycerides, alanine transaminase, aspartate transaminase, alkaline phosphatase, γ-glutamyl transpeptidase, blood urea nitrogen, and creatinine. Urine, hair samples, and nail samples are also collected and stored (Table 3). A urinalysis is performed to assess urine pH, specific gravity, and the presence of blood, protein, glucose, bilirubin, nitrates, ketones, ascorbic acid, leukocytes, and microalbumin. The results of all tests are stored in the database and also given to the participants as an incentive.

Anthropometric characteristics. Height (in cm), weight (in kg), and waist, hip, and wrist circumferences (in cm) are measured using US National Institutes of Health protocols (19). Since measurement error/bias is lowest in the morning, anthropometric data are acquired after sample collection, while the participant is still fasting. Breakfast or a snack is then provided to the participant before proceeding to the next step.

Questionnaires. The PERSIAN Cohort questionnaire consists of 482 items divided into 3 major parts: general, medical, and nutrition. Each part is administered by a trained interviewer. There is no assigned order in which the questionnaire parts need to be completed; therefore, participants are directed to complete those sections based on the availability of the interviewers. The topics covered in the questionnaires are shown in Table 4. The different sections within each part of the questionnaire, such as the socioeconomic status, physical activity,

Downloaded from https://academic.oup.com/aje/advance-article-abstract/doi/10.1093/aje/kwx314/4627678
by Tehran University of Medical Science user
on 24 February 2018

Table 2. Characteristics of the 18 Distinct Study Centers in the PERSIAN Cohort, Iran, 2014–2017

Study Center	Province(s)	Major Ethnic Group(s)	Climate[a]	Biotope	Investigative Institution
Ardabil	Ardabil	Turk	BSk	Forest steppe	Ardabil UMS
Azar	East Azerbaijan	Turk, Azari	BSk	Forest steppe	Tabriz UMS
Bandare Kong	Hormozgan	Arab	BWh	Semi-desert	Hormozgan UMS
Fasa	Fars	Fars/Persian	BSh	Forest steppe	Fasa UMS
Hoveizeh	Khouzestan	Arab	BWh	Semidesert	Ahvaz Jundishapour UMS
Kavar	Fars	Fars/Persian	BSk	Forest Steppe	Shiraz UMS
Kharameh	Fars	Fars, Arab	BSk	Forest steppe	Shiraz UMS
Mashhad	Razavi Khorasan	Fars/Persian	BSk	Semidesert	Mashhad UMS
Rafsanjan	Kerman	Fars/Persian	BWk	Semidesert	Rafsanjan UMS
Ravansar	Kermanshah	Kurd	Csa	Forest steppe	Kermanshah UMS
Sabzevar	Razavi Khorasan	Fars/Persian	BWk	Forest steppe	Sabzevar UMS
Sari	Mazandaran	Tabari	Csa	Forests, woodland	Mazandaran UMS
Shahrekord	Chaharmahal and Bakhtiari	Lur, Bakhtiari	Csa	Forest steppe	Shahrekord UMS
Some’e Sara	Guilan	Gilaki	Csa	Forests, woodland	Guilan UMS
Urmia	West Azerbaijan	Turk, Azari	Csa	Forest steppe	Urmia UMS
Yasuj	Kohgiluyeh and Boyer-Ahmad	Lur	BSk	Forest steppe	Yasuj UMS
Yazd (Shahedieh)	Yazd	Fars/Persian	BWh	Semidesert	Sadoughi Yazd UMS
Zahedan	Sistan and Balouchestan	Balouch	BWh	Desert lowlands	Zahedan UMS

Abbreviations: PERSIAN, Prospective Epidemiological Research Studies in Iran; UMS, University of Medical Sciences.
[a] Based on the Köppen Climate Classification (27): BSh, hot semi-arid climate; BSk, cold semi-arid climate; BWh, hot desert climate; BWk, cold desert climate; Csa, hot-summer Mediterranean climate.

circadian rhythm, or food frequency questionnaire section, were validated alongside the cohort study, and those validation studies will be published separately. Questionnaires are completed online; data are stored in a centralized database and in 3 other locations, being backed up every 30 minutes. All questionnaires are checked for completeness by field supervisors.

At 6 of the cohort study sites (Hoveizeh, Rafsanjan, Azar, Some’e Sara, Yazd, and Zahedan), eye examinations are also performed. This includes a questionnaire regarding eye-specific medical history and a visit by a trained optometrist to assess refractive errors. Persons who meet certain screening criteria receive a complete ophthalmological examination. Fundus photography and slit photos are also obtained for all participants.

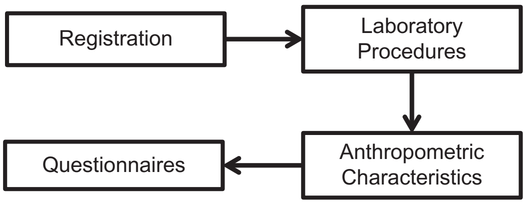

Figure 2. Workflow at the PERSIAN Cohort Study sites, Iran, 2014–2017. Anyone referred to a cohort site will go through these steps to complete the enrollment phase data and sample collection. While the first 3 steps must be followed in order, the general, medical, and nutrition parts of the PERSIAN questionnaire can be completed in any order. PERSIAN, Prospective Epidemiological Research Studies in Iran.

In addition to the standard data collection explained above, which is consistent at all cohort sites, investigators at several sites also perform other assessments based on their research preferences. For example, 3 sites perform electrocardiography, and 2 sites perform bioelectrical impedance analyses for all participants as well.

Follow-up phase data- and sample-gathering. The PERSIAN investigators plan to follow up participants for at least 15 years after enrollment. During this time, participants will receive annual phone calls through which follow-up questionnaires will be completed regarding the occurrence of death or the incidence of any medical events, hospitalizations, or diagnostic/therapeutic care. If a participant has expired or has been diagnosed with a major NCD, investigators will follow the phone call with a house/hospital visit to perform a more thorough follow-up and to collect copies of pertinent medical documents for further evaluation and recording (Figure 3). If needed, medical/physical examinations are performed to formulate a diagnosis. A verbal autopsy form validated in the Iranian population is also completed in the event of death (20). Two trained internists review the medical documents to determine the final diagnosis of disease or cause of death. If a consensus is not reached, a third internist performs a final review of the documents to reach a decision. The same follow-up procedures are followed in the case of self-reported incidence of events.

High migration rates are not anticipated. About 70% of the cohort participants reside in small cities/villages. In general, persons aged 35–70 years who have well-established lifestyles and who live in smaller cities/villages tend to migrate

Downloaded from https://academic.oup.com/aje/advance-article-abstract/doi/10.1093/aje/kwx314/4627678
by Tehran University of Medical Science user
on 24 February 2018

Table 3. Types of Biological Samples Collected From Each Individual in the PERSIAN Cohort and Stored in the Cohort Biobank[a], Iran, 2014–2017

Type of Sample	Amount Collected	Storage Conditions
Blood	25 mL	All blood samples are stored in −70°C freezers
		Whole blood: two 1.5-mL CryoTubes (Micronic, Lelystad, the Netherlands) and one 1-mL CryoTube
		Plasma: two 1.5-mL CryoTubes and five 1-mL CryoTubes
		Buffy coat: three 0.5- to 1-mL CryoTubes
		Serum: two 1-mL CryoTubes
Urine	5 mL	1.5 mL stored in −70°C freezers
Hair	200–300 strands, 1–3 cm long	Stored in foil and zip-lock bags in a cool, dry location
Nail	All fingernails and toenails	Stored in foil and zip-lock bags in a cool, dry location

Abbreviation: PERSIAN, Prospective Epidemiological Research Studies in Iran.
[a] All of the blood samples collected, except for 1 mL of whole blood, plasma, and buffy coat, are stored locally at each study site; the other 3 samples are stored centrally in Tehran.

less. This knowledge comes from our previous experience in the GCS, where use of the same follow-up process in small cities and villages has thus far had a 99% success rate in 10 years (402 of 50,000 lost to follow-up) (15). Nonetheless, since most Iranians currently possess mobile phones, the cell phone numbers of the participant and his/her spouse, as well as contact information from 2 close acquaintances, are obtained at enrollment to recapture participants who may have migrated. These individuals will continue to be followed up. We plan to perform a reassessment in a subsample every 5 years.

Quality assurance and quality control

To ensure that all procedures are performed in accordance with the PERSIAN Cohort protocol, quality assurance (QA) and quality control (QC) measures have been implemented by central and local QA/QC teams. The most important factor in controlling the quality of data collection is a smart data server, which limits many common errors that may be made during data entry. The central QA/QC team remotely monitors daily enrollment on this server, while intermittently checking the data to spot missing information. Reports of these evaluations are sent to the principal investigators at each center. The local QA/QC team also completes monthly evaluations, which are sent to the central team. In addition, to assess interviewer performance, occasional voice/desktop recordings are made without their knowledge but with the consent of the interviewee; these are listened to by the central training team.

Routine surprise inspections are made at each cohort site by the central QA/QC team. During these inspections, besides the evaluation of the cohort personnel and workflow, opinion surveys are completed by random participants. In addition, random individuals who had previously been enrolled are contacted to revisit the cohort site, and they are requestioned and remeasured with regard to some fixed variables so the responses and measurements can be compared with the previously recorded data.

Table 4. Categories[a] of Topics in the PERSIAN Cohort Questionnaire, Iran, 2014–2017

Questionnaire Part	Category Topic
General	Demographic factors
	Socioeconomic status (current)
	Occupational status and history
	Fuel exposures (past and current)
	Lifestyle (past and current)
	Sleep and circadian rhythm (in the past year)
	Physical activity (in the past year)
	Cell phone use (past and current)
	Toxin and pesticide exposure (in the past year)
Medical	Medical history
	Reproductive history (women)
	Medication history
	Family medical history
	Oral and dental health (past and current)
	Personal habits (smoking, alcohol and drug use) (past and current)
	Blood pressure and pulse measurements
	Physical examination
Nutrition	Food frequency questionnaire (130 items—in the past year)
	Dietary habits (current)
	Food preparation and storage techniques (current)
Eye (6 cohort sites only)	Eye-specific medical history
	Eye examination

Abbreviation: PERSIAN, Prospective Epidemiological Research Studies in Iran.
[a] The PERSIAN Cohort questionnaire consists of 482 questions/items in 22 categories. Each part is completed by an interviewer specially trained for its completion.

Downloaded from https://academic.oup.com/aje/advance-article-abstract/doi/10.1093/aje/kwx314/4627678
by Tehran University of Medical Science user
on 24 February 2018

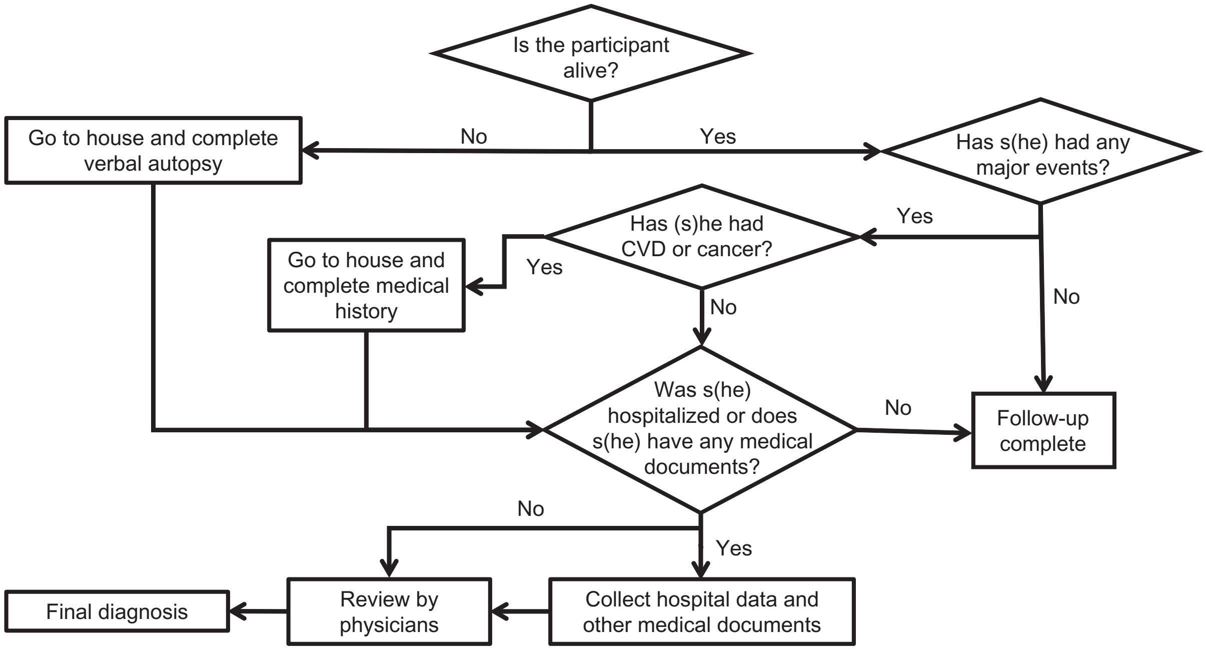

Figure 3. Follow-up procedures used in the PERSIAN Cohort Study, Iran, 2014–2017. As part of the follow-up process, annual phone calls are made to all participants and, based on this flow chart, the necessary questionnaires are completed. CVD, cardiovascular disease; PERSIAN, Prospective Epidemiological Research Studies in Iran.

In addition to these procedures, prearranged national and international experts are also invited to visit and evaluate cohort sites occasionally.

DISCUSSION

In population-based cohort studies, a well-defined population is followed longitudinally for assessment of the relationships between different exposures and outcomes. Due to the longitudinal nature of cohort studies, they are useful in determining the natural history of diseases and their cumulative incidence, incidence rates, and mortality rates and in identifying risk factors associated with diseases, as well as in the study of growth, development, and aging (5).

Famous cohort studies around the world, such as the Framingham Heart Study, the Rotterdam Study, the Asia-Pacific Cohort Studies Collaboration, etc., have greatly contributed to medical and health-related knowledge, and many current health recommendations arose from their findings. In Iran, the GCS has revealed important risk factors associated with esophageal cancer, as northern Iran falls into the Asian esophageal cancer belt, which stretches from Iran to China. Hot tea drinking, opium use, and low fruit/vegetable intake were found to be some of the risk factors associated with esophageal cancer (6). Since the discovery of these risk factors, local health officials have sought to raise public awareness for esophageal cancer prevention.

The leading cause of death in the GCS, however, was not esophageal cancer. Similar to worldwide trends, the leading causes of death were CVD and cerebrovascular disease, accounting for nearly 50% of all deaths, followed by all cancers combined and other NCDs such as renal, hepatic, and respiratory diseases (R.M., unpublished data, 2016). Studies carried out in other regions of Iran have also found CVD to be the leading cause of death; however, there are regional differences in the risks of specific diseases (8–12, 21). Therefore, a study encompassing different regions of Iran with different exposures was needed to first determine the burden of NCDs in Iran and second identify risk factors associated with them, in order to tailor public health policies to the needs of the Iranian population. Thus, the PERSIAN Cohort Study was launched in 2014 in 18 geographically distinct areas of Iran. All major Iranian ethnic groups are included in this study, making it ideal for individualized care. The PERSIAN Cohort will be the largest such cohort in Iran, the Middle East, and northern Africa and will have the largest biobank in the region as well, making it ideal for future studies on genetics, disease biomarkers, risk factors, protective factors, etc.

PERSIAN was designed with careful planning to limit biases. Financial constraints made it impossible to have a fully automated laboratory and biobank, as is seen in some modern biobanks worldwide. Nevertheless, every effort was made to limit variations among the cohort sites by centrally purchasing the same laboratory equipment for all centers and having the same individual train all laboratory technicians on the study protocols. This also applies to other sectors of data collection; all questionnaire interviewers were trained by the same individuals in order to keep data collection as consistent as possible, and this is one of the greatest strengths of the study. A uniform protocol is used in all centers, and all cohort personnel are local to each site to avoid biases caused by cultural or language/dialect barriers.

Downloaded from https://academic.oup.com/aje/advance-article-abstract/doi/10.1093/aje/kwx314/4627678
by Tehran University of Medical Science user
on 24 February 2018

Standardized equipment and protocols are being used to measure anthropometric characteristics. Local and central QA/QC teams conduct routine monitoring and evaluations to ensure that the data/samples are being collected per protocols.

One limitation of PERSIAN is that it does not include a random sample of the Iranian population, which may affect some descriptive measures; however, PERSIAN shares this limitation with other large population-based cohort studies around the world (22–24). Nevertheless, the associations identified in these studies can be generalized to larger populations. In PERSIAN, instead of a random sample, efforts were made to include all of the major ethnic groups living in different geographical regions of Iran, giving researchers an enormous data set on various exposures, such as diet/lifestyle, environmental factors, socioeconomic factors, etc. Adjustments can be made during analysis to obtain better representativeness, if needed. Another limitation is the lack of funding for genetic studies. Genome-wide association studies have been previously performed in the GCS, using international funding (25, 26). If funding becomes available, these types of studies can be in the future of PERSIAN as well.

Ensuring that persons of both sexes participate in the study equally is an enrollment challenge, as lower participation is seen among working men. Special arrangements are made so that people unable to participate in the morning can give only their fasting blood samples at that time and then return in the afternoon to complete the questionnaires. Overcoming the most important challenge—limiting the percentage of participants lost to follow-up—is also ahead. Providing incentives (such as possible free medical care), along with raising awareness about the benefits of the cohort study, is necessary to encourage participation.

In conclusion, the PERSIAN Cohort investigators started enrollment in the year 2014 to assess NCDs in the ethnically diverse population of Iran. Although it is too soon to report results, cohort profiles from each site should emerge soon after the completion of the enrollment phase.

ACKNOWLEDGMENTS

Author affiliations: Liver and Pancreatobiliary Diseases Research Center, Digestive Diseases Research Institute, Tehran University of Medical Sciences (UMS), Tehran, Iran (Hossein Poustchi, Sareh Eghtesad, Zahra Mohammadi, Zahra Mahmoudi, Amaneh Shayanrad, Farzin Roozafzai, Mahdi Sheikh, Alireza Jalaeikhoo); Department of Biology, School of Computer, Mathematical, and Natural Sciences, Morgan State University, Baltimore, Maryland (Farin Kamangar); Digestive Oncology Research Center, Digestive Diseases Research Institute, Tehran UMS, Tehran, Iran (Farin Kamangar, Reza Malekzadeh); Division of Cancer Epidemiology and Genetics, National Cancer Institute, Bethesda, Maryland (Arash Etemadi); Department of Health Sciences Education Development, School of Public Health, Tehran UMS, Tehran, Iran (Abbas-Ali Keshtkar); Department of Clinical Nutrition, School of Nutritional Sciences and Dietetics, Shahid Beheshti UMS, Tehran, Iran (Azita Hekmatdoost); Liver and Gastrointestinal Diseases Research Center, Tabriz UMS, Tabriz, Iran (Mohammad Hossein Somi); Department of Gastroenterology, Gastrointestinal and Liver Diseases Research Center, Guilan UMS, Rasht, Iran (Fariborz Mansour-Ghanaei); Research Center for Environmental Determinants of Health, School of Public Health, Kermanshah UMS, Kermanshah, Iran (Farid Najafi); Noncommunicable Diseases Research Center, Fasa UMS, Fasa, Iran (Ehsan Bahramali); Department of Occupational Medicine, Faculty of Medicine, Shahid Sadoughi UMS, Yazd, Iran (Amirhoushang Mehrparvar); Health Promotion Research Center, Zahedan UMS, Zahedan, Iran (Alireza Ansari-Moghaddam); School of Public Health and Health Sciences Research Center, Mazandaran UMS, Sari, Iran (Ahmad Ali Enayati); Department of Cardiology, School of Medicine, Rafsanjan UMS, Rafsanjan, Iran (Ali Esmaeili Nadimi); Department of Epidemiology, School of Health, Shiraz UMS, Shiraz, Iran (Abbas Rezaianzadeh); Department of Otolaryngology, Head and Neck Surgery, Hearing and Speech Research Center, Ahvaz Jundishapur UMS, Ahvaz, Iran (Nader Saki); Eye Research Center, Farabi Eye Hospital, Tehran UMS, Tehran, Iran (Fatemeh Alipour); Department of Pediatrics, Child Growth and Development Research Center, Research Institute for Primordial Prevention of Non-Communicable Diseases, Isfahan UMS, Isfahan, Iran (Roya Kelishadi); Iranian National Center for Addiction Studies, Tehran UMS, Tehran, Iran (Afarin Rahimi-Movaghar); Department of Statistics and Epidemiology, Faculty of Health Sciences, Tabriz UMS, Tabriz, Iran (Nayyereh Aminisani); and Department of Oncological Sciences, Icahn School of Medicine at Mount Sinai, New York, New York (Paolo Boffetta).

H.P. and S.E. contributed equally to this report and are considered co–first authors.

We thank all of the investigators and personnel at the different PERSIAN cohort centers, whose work made this study possible.

Conflict of interest: none declared.

REFERENCES

1. Institute for Health Metrics and Evaluation. *GBD Profile: Iran. Global Burden of Diseases, Injuries, and Risk Factors Study 2010*. Seattle, WA: Institute for Health Metrics and Evaluation; 2010. http://www.healthdata.org/sites/default/files/files/country_profiles/GBD/ihme_gbd_country_report_iran.pdf. Accessed September 28, 2016.
2. World Health Organization. *Noncommunicable Diseases Country Profiles 2014*. Geneva, Switzerland: WHO Press; 2014. http://apps.who.int/iris/bitstream/10665/128038/1/9789241507509_eng.pdf. Accessed September 28, 2016.
3. World Health Organization. *Global Status Report on Noncommunicable Diseases 2014*. Geneva, Switzerland: WHO Press; 2014. http://apps.who.int/iris/bitstream/10665/148114/1/9789241564854_eng.pdf. Accessed September 28, 2016.
4. World Economic Forum. *From Burden to "Best Buys": Reducing the Economic Impact of Non-Communicable Diseases in Low- and Middle-Income Countries*. Geneva, Switzerland: World Health Organization; 2011.
5. Breslow NE, Day NE, eds. *Statistical Methods in Cancer Research. Vol. 2. The Design and Analysis of Cohort Studies*.

Downloaded from https://academic.oup.com/aje/advance-article-abstract/doi/10.1093/aje/kwx314/4627678
by Tehran University of Medical Science user
on 24 February 2018

(IARC Scientific Publications no. 82). Lyon, France: International Agency for Research on Cancer; 1987.
6. Nasrollahzadeh D, Kamangar F, Aghcheli K, et al. Opium, tobacco, and alcohol use in relation to oesophageal squamous cell carcinoma in a high-risk area of Iran. *Br J Cancer*. 2008; 98(11):1857–1863.
7. Islami F, Pourshams A, Nasorllahzadeh D, et al. Tea drinking habits and oesophageal cancer in a high risk area in northern Iran: population based case-control study. *BMJ*. 2009;338:b929.
8. Malekzadeh F, Marshall T, Pourshams A, et al. A pilot double-blind randomised placebo-controlled trial of the effects of fixed-dose combination therapy ("polypill") on cardiovascular risk factors. *Int J Clin Pract*. 2010;64(9):1220–1227.
9. Sheidaei A, Gohari K, Kasaeian A, et al. National and subnational patterns of cause of death in Iran 1990–2015: applied methods. *Arch Iran Med*. 2017;20(1):2–11.
10. Mousavi SM, Gouya MM, Ramazani R, et al. Cancer incidence and mortality in Iran. *Ann Oncol*. 2009;20(3):556–563.
11. Esmaeimzadeh N, Salahi-Moghaddam A, Khoshdel AR. Geographical distribution of important cancers in Iran. *Hormozgan Med J*. 2015;19(2):74–82.
12. Khazaei S, Ayubi E, Soheylizad M, et al. Incidence and distribution of common cancers among Iranian children. *Middle East J Cancer*. 2017;8(1):39–42.
13. Khademi H, Malekzadeh R, Pourshams A, et al. Opium use and mortality in Golestan Cohort Study: prospective cohort study of 50 000 adults in Iran. *BMJ*. 2012;344:e2502.
14. Etemadi A, Kamangar F, Islami F, et al. Mortality and cancer in relation to ABO blood group phenotypes in the Golestan Cohort Study. *BMC Med*. 2015;13:8.
15. Etemadi A, Khademi H, Kamangar F, et al. Hazards of cigarettes, smokeless tobacco and waterpipe in a Middle Eastern population: a cohort study of 50 000 individuals from Iran. *Tob Control* 2016;26(6):674–682.
16. Pourshams A, Khademi H, Malekshah AF, et al. Cohort profile: the Golestan Cohort Study—a prospective study of oesophageal cancer in northern Iran. *Int J Epidemiol*. 2010; 39(1):52–59.
17. Roshandel G, Sadjadi A, Aarabi M, et al. Cancer incidence in Golestan Province: report of an ongoing population-based cancer registry in Iran between 2004 and 2008. *Arch Iran Med*. 2012;15(4):196–200.
18. Rahmati A, Shakeri R, Khademi H, et al. Mortality from respiratory diseases associated with opium use: a population-based cohort study. *Thorax*. 2017;72(11):1028–1034.
19. Centers for Disease Control and Prevention. *National Health and Nutrition Examination Survey (NHANES). Anthropometry Procedures Manual*. Atlanta, GA: Centers for Disease Control and Prevention; 2007. https://www.cdc.gov/nchs/data/nhanes/nhanes_07_08/manual_an.pdf. Accessed September 28, 2016.
20. Khademi H, Etemadi A, Kamangar F, et al. Verbal autopsy: reliability and validity estimates for causes of death in the Golestan Cohort Study in Iran. *PLoS One*. 2010;5(6):e11183.
21. Keramati AR, Fathzadeh M, Go GW, et al. A form of the metabolic syndrome associated with mutations in *DYRK1B*. *N Engl J Med*. 2014;370(20):1909–1919.
22. Belanger CF, Hennekens CH, Rosner B, et al. The Nurses' Health Study. *Am J Nurs*. 1978;78(6):1039–1040.
23. Schatzkin A, Subar AF, Thompson FE, et al. Design and serendipity in establishing a large cohort with wide dietary intake distributions: the National Institutes of Health-American Association of Retired Persons Diet and Health Study. *Am J Epidemiol*. 2001;154(12):1119–1125.
24. International Agency for Research on Cancer, World Health Organization. EPIC Study. http://epic.iarc.fr/about/about.php. Accessed April 4, 2017.
25. Liu JZ, van Sommeren S, Huang H, et al. Association analyses identify 38 susceptibility loci for inflammatory bowel disease and highlight shared genetic risk across populations. *Nat Genet*. 2015;47(9):979–986.
26. Sitas F, Egger S, Urban MI, et al. InterSCOPE Study: associations between esophageal squamous cell carcinoma and human papillomavirus serological markers. *J Natl Cancer Inst*. 2012;104(2):147–158.
27. Climate-Data.org. Climate: Iran. https://en.climate-data.org/country/66/. Accessed September 28, 2016.

Downloaded from https://academic.oup.com/aje/advance-article-abstract/doi/10.1093/aje/kwx314/4627678
by Tehran University of Medical Science user
on 24 February 2018
