# Supplementary material for: Cohort profile: Bandar Kong prospective study of chronic non-communicable diseases
Source: PLoS One. 2022 May 12;17(5):e0265388. doi: 10.1371/journal.pone.0265388 (PMC9098057; doi:10.1371/journal.pone.0265388)
Supplement: S4 File — (PDF) [file pone.0265388.s004.pdf]

مرحله اول طرح کوهورت کشوری

پرسشنامه اصلی

نام و نام خانوادگی فرد پاسخگو.. IR.PC.□□□□□□□□□□

تاریخ انجام مصاحبه و پرسشگری:	۱۳ / /
محل مصاحبه	استان: .... شهرستان .... شهر ....<br>روستا .... مرکز بهداشتی-درمانی شهری، روستایی ....
نام و نام خانوادگی پرسشگر<br>امضاء	

G. مشخصات عمومی

G 1- نام و نام خانوادگی :	
G2 - کد ملی □□□□□□□□□□	
G3-کدپستی۱۰رقمی □□□□□□□□□□<br>G4- پست الکترونیک	G5-تلفن ثابت<br>□□□□□□□□□□<br>G6- تلفن همراه<br>□□□□□□□□□□
G7- آدرس کامل خانوار: استان.......................شهرستان.........................شهر.............................. روستا.........................خیابان................<br>کوچه...پلاک......................................	
G8- جنس: □ ۱- مرد □ ۲- زن	□
G9- تاریخ تولد (بر حسب سال شمسی)	□□□□/□□/□□
G10-1- تعداد سالهای تحصیل (بدون احتساب سالهای مردودی):<br><br>G10-2- آخرین مدرک تحصیلی دریافت شده؟<br>۱- ابتدایی ۲- راهنمایی ۳- دیپلم۴- فوق دیپلم ۵- لیسانس۶- فوق لیسانس ۷- دکترا	□□<br><br>□
G11- قومیت پدر: ۱- فارس ۲-آذری ۳-بلوچی ۴- کردی<br>۵-لری ۶- عربی ۷- ترکمن ۸- تالش ۹- زابلی ۱۰- گیلکی<br>۱۱- سایر (نام ببرید)	□
G12–قومیت مادر: : ۱- فارس ۲-آذری ۳-بلوچی ۴- کردی<br>۵-لری ۶- عربی ۷- ترکمن ۸- تالش ۹- زابلی ۱۰- گیلکی<br>۱۱- سایر (نام ببرید)	□
G13- محل تولد: استان........................ شهرستان ....<br>شهر .... روستا	

G14- مشخصات دو نفراز اعضای خانواده یا دوستان :

نام و نام خانوادگی	۱.	۲.
نسبت آشنایی	۱.	۲.
تلفن ثابت منزل	۱.	۲.
تلفن همراه	۱.	۲.

G15- موقعیت فرد درخانوار:<br>۱)پدر ۲) مادر ۳) فرزند ۴)پدربزرگ ومادربزرگ ۵) عمه وعمو<br>۶) خاله /دایی ۷) سایر باذکر نسبت.........	☐
G16- وضعیت تأهل: ۱- مجرد(هرگز ازدواج نکرده)۲-متأهل ۳-همسر مرده ، بیوه ۴-مطلقه<br>۵- سایر	☐
G17 – چند بار ازدواج کرده اید؟	☐
G18- آیا ازدواج فامیلی بوده است؟ (فامیل درجه یک) ☐ ۱- بلی ☐ ۲-خیر	☐
G19 – سن در زمان اولین ازدواج؟	☐☐

SE . وضعیت اجتماعی اقتصادی

سوالات و گزینه ها			کد
SE1- وضعیت تملک مسکن مورد استفاده خانوار چگونه است؟<br>☐۱- ملک شخصی خانوار<br>☐۲-ملک رهنی یا استیجاری<br>☐۳- ملک سازمانی (دولتی یا خصوصی)<br>☐۴- ملک خویشاوندان یا سایرین به صورت امانتی (در صورتیکه هیچ وجهی پرداخت نشود)<br>☐۵- سایر			☐
SE2 - متراژ واحد مسکونی چقدر است؟ (بدون در نظر گرفتن محل نگهداری حیوانات،باغچه،ایوان، کارگاه و حیاط وغیره چقدراست)؟ (............................. مترمربع)			☐☐☐☐
SE3-تعداد اتاق‌های خواب محل سکونت فعلی ؟ *(بدون در نظر گرفتن پذیرایی و آشپزخانه)*			☐
SE4- تعداد افرادی از خانواده که با هم در منزل فعلی شما زندگی می‌کنند؟			☐☐
SE5- کدامیک از وسایل زیر را در اختیار دارید؟			
۱- فریزر جداگانه	☐ ۱- دارد	☐ ۲- ندارد	☐
۲- ماشین لباسشویی	☐ ۱- دارد	☐ ۲- ندارد	☐
۳- ماشین ظرفشویی	☐ ۱- دارد	☐ ۲- ندارد	☐
۴- رایانه/لپ تاب	☐ ۱- دارد	☐ ۲- ندارد	☐
۵- دسترسی به اینترنت	☐ ۱- دارد	☐ ۲- ندارد	☐
۶- خودرو سواری:	☐ ۱- دارد	☐ ۲- ندارد	☐
۱)کمتر از ۲۰ میلیون تومان<br>۲)۵۰-۲۰ میلیون تومان<br>۳)۵۰- ۱۰۰میلیون تومان<br>۴)بیشتر از ۱۰۰ میلیون تومان	☐		
۷ـ موتورسیکلیت	☐ ۱- دارد	☐ ۲- ندارد	☐

۸ـ نوع تلویزیون رنگی<br>۱) معمولی<br>۲) پلاسما<br>۹ـ حمام داخل منزل<br>۱۰ـ جارو برقی	☐ ۱- دارد<br>☐<br>☐ ۱- دارد<br>☐ ۱- دارد	☐ ۲- ندارد<br>☐ ۲- ندارد<br>☐ ۲- ندارد	☐<br>☐<br>☐
SE6- کدامیک از وسایل زیر در اختیار فرد قرار داشته و از آن استفاده می کند؟			
۱ـ تلفن همراه<br>۲- رایانه<br>۳- لپ تاپ<br>۴- دسترسی به اینترنت<br>۵- خودرو سواری:<br>۱)کمتر از ۲۰ میلیون تومان<br>۲)۵۰-۲۰ میلیون تومان<br>۳)۵۰- ۱۰۰میلیون تومان<br>۴)بیشتر از ۱۰۰ میلیون تومان	☐ ۱- دارد<br>☐ ۱- دارد<br>☐ ۱- دارد<br>☐ ۱- دارد<br>☐ ۱- دارد<br>☐	☐ ۲- ندارد<br>☐ ۲- ندارد<br>☐ ۲- ندارد<br>☐ ۲- ندارد<br>☐ ۲- ندارد	☐<br>☐<br>☐<br>☐<br>☐
SE7- تعداد کتابهای غیر درسی و غیر شغلی که در یکسال قبل مطالعه کرده اید (شامل کتابهای آسمانی و دعا نمی گردد)<br>SE8ـ تعداد مسافرتهای خارج از ایران در تمام عمر : ☐☐<br>۱) زیارتی ☐☐ ۲) غیر زیارتی ☐☐ (0)هرگز<br>SE9ـ تعداد مسافرتهای داخل ایران در ده سال گذشته (زیارتی وگردشگری که حداقل ۱۰۰ کیلومتر از محل سکونت فاصله داشته است)) (0) هرگز ☐☐			☐☐<br>☐☐<br>☐☐

O . سوابق شغلی

سوالات و گزینه ها	کد
O1- آیا در حال حاضر شاغل هستید؟ ☐ ۱- بلی ☐ ۲-خیر	☐
O2ـ نوع شغل را نام ببرید؟	☐
O3ـ گروه شغلی را مشخص کنید؟ بر اساس ISCO-88	☐☐

O4- تمام شغل‌هایی را که حداقل به مدت **یکسال** بدان اشتغال داشتید، از زمان اولین شغل به ترتیب نام ببرید. در صورت اشتغال به چند شغل در یک دوره زمانی، همه آنها را ذکر کنید. لازم است زمان کاری شغل های ذکر شده حداقل ۸ ساعت در هفته باشد. مشاغلی را که در منزل انجام می‌گیرند (مانند فرشبافی و خانه داری) نیز ذکر نمایید.

ردیف	از سن	تا سن	عنوان شغل	کدگروه شغلی
۱	☐☐	☐☐		☐☐☐
۲	☐☐	☐☐		☐☐☐
۳	☐☐	☐☐		☐☐☐
۴	☐☐	☐☐		☐☐☐

O5ـ منبع اصلی درآمد شما چیست؟ ☐

۱- بیمه ۲- خود فرد ۳- همسر ۴- فرزند یا فرزندان ۵- کمیته امداد ـ بهزیستی ۶ - پدر و مادر ۷- سایر

O6ـ اگر متاهل هستید شغل همسر شما چیست ؟ .... ☐☐☐

کد گروه شغلی مشخص گردد؟ ☐☐☐

F. وضعیت سوخت ومحل زندگی

منطقه و نوع محل سکونت خود را که حداقل یکسال در آن زندگی کرده اید از زمان تولد بنویسید:

ردیف	از سن	تا سن	استان	شهر	روستا	نوع خانه	نوع سوخت گرمایش	نوع سوخت پخت و پز	سیستم گرمایش
	☐☐	☐☐				☐	☐	☐	☐
	☐☐	☐☐				☐	☐	☐	☐
	☐☐	☐☐				☐	☐	☐	☐
	☐☐	☐☐				☐	☐	☐	☐
	☐☐	☐☐				☐	☐	☐	☐
	☐☐	☐☐				☐	☐	☐	☐

کد نوع خانه

۱-آجرو آهن
۲- چوب و آجر
۳- بتون آرمه
۴- گچ و سنگ
۵-سایر

کد سیتم گرمایش

۱-بخاری برقی
۲- بخاری دارای دودکش
۳- شومینه
۴- بخاری بدون دودکش/چراغ
۵- بخاری گازی
۶- شوفاژ
۷- سایر

کد نوع سوخت

۱-نفت/گازوییل
۲- چوب و هیزم
۳- فضولات حیوانی
۴- گاز
۵- زغال سنگ
۶- برق
۷- سایر

l. سبک زندگی

L1- منبع اصلی آب آشامیدنی شما چیست؟<br>۱- چاه ۲- آب رودخانه ۳- آب چشمه 4 - آب لوله کشی<br>۵- آب معدنی ۶- آب تانکر ۷- آب انبار ۸- سایر	☐☐☐
L2- در صورت مصرف آب لوله کشی،چندسال است که از آن استفاده می کنید؟<br>(اگر همیشه از آب لوله کشی استفاده کرده است کد ۹۹ وارد شود)<br>L3-1- قبل از استفاده از آب لوله کشی، منبع اصلی آب آشامیدنی شما چه بوده است؟<br>۱ - چاه ۲- آب رودخانه ، رود ۳- چشمه ۴- سایر<br>L3-2- آیا این منبع بهداشتی بوده است؟ (تحت نظارت آب و فاضلاب/ مرکز بهداشت)۱- بلی ۲- خیر	☐☐<br>☐<br>☐
L4- نوع آشپزخانه : ☐۱- داخل خانه مجزا ☐۲- داخل خانه (open) ☐۳- خارج از خانه	☐
L5- آیا از هود در آشپزخانه استفاده میکنید ؟ ☐ ۱- همیشه ☐ ۲- گاهی ☐۳- ندارد	☐
L6- آیا آشپزخانه پنجره دارد ؟ ☐ ۱- دارد ☐ ۲- ندارد<br>L6-1- آیا بهنگام آشپزی پنجره آن باز است ؟ ☐۱- بلی ☐۲- خیر	☐<br>☐
L7- تعداد کل پنجره ها در منزل ؟	☐☐
L8- بطور معمول روزی چندبار پنجره ها باز می شوند:<br>در فصول گرم --- بار در روز<br>در فصول سرد --- بار در روز	☐<br>☐

L9- آیا سابقه تماس با حیوانات را داشته اید؟ ☐۱- بلی ☐۲- خیر

اگر سابقه تماس با حیوانات داشته اید آن را ذکر نمایید:

☐

از سن	تا سن	سطح تماس	حیوان (حیوانات)
|__||__|	|__||__|	|__|	|__|
|__||__|	|__||__|	|__|	|__|

سطح تماس

(۱) بعضی مواقع (برای مثال محل نگهداری حیوانات در دویست متری محل کار یا زندگی فرد است)

(۲) حداقل یکبار در دو هفته ولی کمتر از یکبار در روز(برای مثال محل نگهداری حیوانات در همسایگی محل کار یا زندگی فرد است)

(۳) تماس روزانه (برای مثال نگهداری حیوانات در محل کار یا زندگی فرد)

(۴) تماس روزانه و نزدیک با حیوانات (شغلهای مربوط به تغذیه و تمیز کردن حیوانات و کشتارگاه)

حیوانات

(۱) اسب سانان (مانند اسب، قاطر، الاغ, شتر) (۲) نشخوار کنندگان (مانند گوسفند، بز، گاو)

(۳) سگ (۴) طیور

PERSIAN Cohort

R. سوالات تاریخچه باروری (مختص زنان)

سؤالات و گزینه‌ها	کد
R1 – سن شروع قاعدگی (به سال): (اگر نمی دا ند ، عدد ۸۸ را وارد نمائید. اگر هرگز پریود نشده عدد ۸۹ وارد شود)	☐☐
R2- آیا در حال حاضر حامله هستید؟<br>☐۱- بلی ☐۲-خیر ☐۳- نمیدانم	☐
R3- تعداد حاملگی های قبلی : ☐☐	☐☐
R4- تعداد زایمانهای موالید زنده : ☐☐<br>R5-سابقه مرده زایی : ☐ ۱- دارد ☐ ۲- ندارد	☐☐<br>☐
R6- سن مادر هنگام اولین تولد نوزاد زنده : ☐☐<br>R7- سن مادر بهنگام اولین حاملگی : ☐☐	☐☐<br>☐☐
R8- تعداد سقط : ☐☐<br>R9- سن بهنگام اولین سقط : ☐☐	☐☐<br>☐☐
R10- مجموع مدت شیردهی (برحسب ماه) : ☐☐	☐☐
R11- سابقه برداشتن تخمدان: ☐ ۱- دارد، یکطرفه ☐ ۲- دارد، دوطرفه ☐ ۳- ندارد	☐
R12- سن در زمان برداشتن تخمدان (به سال) : ☐☐	☐☐
R13- سابقه توبکتومی ☐ ۱- دارد ۲- ندارد<br>R14- سابقه هیسترکتومی: ☐ ۱- دارد ☐ ۲- ندارد<br>R15- سن به سال در زمان هیسترکتومی ☐☐	☐<br>☐<br>☐☐
R16- سابقه نازایی : ☐ ۱- دارد ☐ ۲- ندارد<br>R17- سابقه مصرف داروهای مرتبط با نازایی: ☐ ۱- دارد ☐ ۲- ندارد	☐<br>☐
R18- داروهای ضد بارداری (خوراکی (OCP)، آمپول DMPA، سیکلوفم، ...) مصرف می کرده/می کند؟<br>(اگر در حال حاضر مصرف میکند، حال گزارش شود و اگر در گذشته مصرف میکردند، گذشته.)<br>☐1- بلی ☐ ۲- خیر	☐

روش پیشگیری	۱-بلی ۲- خیر	از سن	تا سن	مدت زمان
قرصهای پیشگیری از بارداری	|__	|__||__|	|__||__|	|__||__| ماه
	|__	|__||__|	|__||__|	|__||__| ماه
ایمپلنت	|__	|__||__|	|__||__|	|__||__| ماه
	|__	|__||__|	|__||__|	|__||__| ماه
تزریق پروژسترون طولانی مدت	|__	|__||__|	|__||__|	|__||__| ماه
	|__	|__||__|	|__||__|	|__||__| ماه
وسایل داخل رحمی (IUD)	|__	|__||__|	|__||__|	|__||__| ماه
	|__	|__||__|	|__||__|	|__||__| ماه

R-19: سن یائسگی (به سال) : (اگر هنوز منوپوز نشده، کد ۸۹ و اگر نمی دا ند ، عدد ۸۸ را وارد نمائید) ☐☐

R-20- آیا یایسگی طبیعی بوده است؟ ☐۱- بلی ☐۲-خیر

R21- داروهای جایگزینی هورمونی (استروژن، پروژسترون) مصرف می کرده/می کند؟ ☐

(اگر در حال حاضر مصرف میکند، حال گزارش شود و اگر در گذشته مصرف میکردند، گذشته.)

☐ ۱- بلی ☐ ۲- خیر ☐ ۳- نمی دانم

در صورت بلی، نوع داروی مورد استفاده (در صورتی که به خاطر دارید)	از سن	تا سن	مدت زمان استفاده (ماه)
	|__||__|	|__||__|	|__||__|
	|__||__|	|__||__|	|__||__|
	|__||__|	|__||__|	|__||__|

R22 –سابقه غربالگری سرطان پستان یا دهانه رحم دارد؟ ☐

☐ ۱- بلی ☐ ۲- خیر ☐ ۳- نمی دانم

در صورت بلی، جدول ذیل تکمیل شود:

نوع غربالگری	بلی	سن آخرین تست	دفعات تست
معاینه پستان توسط پزشک/ماما	|__|	|__||__|	|__|
ماموگرافی	|__|	|__||__|	|__|
پاپ اسمیر	|__|	|__|__	|__|

C. سوابق بیماریهای مزمن که توسط پزشک پرسیده می شود

شماره	بیماری	۱- بلی<br>۲- خیر<br>۳- نمی دانم	مدت زمان بیماری(ماه)	سن در زمان تشخیص	تحت درمان است؟<br>۱-بلی<br>۲-خیر
C1	بیماری دیابت	☐	☐☐	☐☐	☐
C2	افزایش فشار خون	☐	☐☐	☐☐	☐
C3	بیماری ایسکمیک قلبی شامل: نارسائی قلبی و آنژین	☐	☐☐	☐☐	☐
C4	سکته قلبی	☐	☐☐	☐☐	☐
C5	آیا هنگام راه رفتن تند یا بالا رفتن از سربالایی احساس سنگینی،درد یا سوزش در پشت جناغ سینه خود دارید؟ ☐<br>۱- بلی ☐ ۲- خیر				☐
C6	سکته مغزی	☐	☐☐	☐☐	☐
C7	نارسایی کلیه	☐	☐☐	☐☐	☐
C8	آیا سابقه ورم در بدن،خصوصا پاها را داشته اید؟ ☐۱- بلی☐ ۲- خیر				☐
C9	آیا سابقه تغییر رنگ ادرار خصوصا ادرار شبه خونی داشته اید؟ ☐ ۱- بلی☐ ۲- خیر				☐
C10	آیا سابقه سوزش ادرار،تکرر ادرار، شب ادراری و شب شاشی داشته اید؟ ☐۱- بلی☐ ۲- خیر				☐
C11	آیا سابقه آزمایش ادرار غیرطبیعی (خون،رک،پروتین،لکوسیت،RBC) داشته اید؟ ☐۱- بلی☐ ۲- خیر				☐
C12	آیا در یک سال گذشته دچار درد و سوزش زیر جناق سینه شده اید؟ ☐۱- بلی ☐ ۲- خیر				☐
C13	اگر بلی تعداد دفعات آن را مشخص کنید:۱- تقریبا هر روز ۲- چند بار در هفته۳- چند بار در ماه ۴- گاهی				☐
C14	آیا در یک سال گذشته دچار برگشت غذا از معده به مری و حلق شده ید؟ ☐۱- بلی ☐ ۲- خیر				☐
C15	اگر بلی تعداد دفعات آن را مشخص کنید:				
	(۱) تقریبا هر روز (۲) چند بار در هفته (۳) چند بار در ماه (۴) گاهی ☐				☐
C16	آیا تاکنون تشخیص بیماری رفلاکس اسید از معده به مری داشته اید؟ ۱- بلی☐ ۲- خیر				☐
C17	آیا در یک سال گذشته دچار نفخ و اتساع شکم بخصوص بعد از مصرف غذا شده ید؟ ☐ ۱- بلی☐ ۲- خیر				☐
C18	اگر بلی تعداد دفعات آن را مشخص کنید:<br>۱) تقریبا هر روز (۲) چند بار در هفته (۳) چند بار در ماه (۴) گاهی				☐
C19	برنامه اجابت مزاجی شما چگونه است؟<br>۱) روزانه______ بار<br>۲) هفته ای ______ بار<br>۳) ماهانه______ بار				
C20	آیا سابقه دفع خون تازه در مدفوع داشته اید؟ ☐ ۱- بلی☐ ۲- خیر				☐

PERSIAN Cohort

C21	آیا سابقه کاهش وزن ناخواسته داشته اید؟ (بدون رژیم غذایی و بیش از ۵ کیلوگرم در یکماه) ۱- بلی ۲- خیر				☐
C22	آیا سابقه زردی چشم یا پوست بدن در طول زندگی داشته اید؟<br>☐۱- بلی ☐ سن__________هفته/ ماه/سال ۲- خیر				☐
C23	آیا سابقه بیماری کبد چرب (با تشخیص پزشک) دارید؟ ☐۱- بلی ☐ ۲- خیر				☐
C24	آیا سابقه بیماری هپاتیت B دارید؟ ۱- بلی ☐ ۲- خیر				☐
C25	آیا سابقه بیماری هپاتیت C دارید؟ ۱- بلی ☐ ۲- خیر				☐
C26	بیماریهای مزمن ریوی<br>(سل. آسم)	☐	☐☐	☐☐	☐
C27	در یکسال گذشته حداقل به مدت ۲هفته سرفه داشته اید ؟ ☐ ۱- بلی☐ ۲- خیر<br>اگر بلی : ☐۱- خلط دار ☐۲- بدون خلط				☐<br>☐
C28	آیا در یکسال گذشته حداقل به مدت ۲ هفته تنگی نفس به همراه خس خس سینه داشته اید؟۱- بلی ۲- خیر				☐
	بیماری	۱- بلی<br>۲- خیر<br>۳- نمی دانم	مدت زمان بیماری(ماه)	سن در زمان تشخیص	تحت درمان است؟<br>۱-بلی ۲-خیر
C29	بیماریهای تیرویید	☐	☐☐	☐☐	☐
C30	سنگ کلیه	☐	☐☐	☐☐	☐
C31	سنگ کیسه صفرا	☐	☐☐	☐☐	☐
C32	بیماریهای روماتیسمی	☐	☐☐	☐☐	☐
C33	سرطان<br>۱- معده<br>۲- کولورکتال<br>۳- پروستات<br>۴- پستان<br>۵- سایر ذکر گردد	☐<br>☐<br>☐	☐☐	☐☐	☐<br>☐
C34	صرع	☐	☐☐	☐☐	☐
C35	سردرد مزمن و راجعه	☐	☐☐	☐☐	☐
C36	افسردگی با تشخیص پزشک	☐	☐☐	☐☐	☐
C37	هرگونه اختلال روانپزشکی دیگر	☐	☐☐	☐☐	☐
C38	اختلال یادگیری که باعث کاهش پیشرفت در تحصیل شده باشد	☐	☐☐	☐☐	☐
C39	اختلال حافظه که سبب اختلال جدی در عملکرد روزانه شود	☐	☐☐	☐☐	☐☐

PERSIAN Cohort

C40	آیا شما هرگز دچار مشکل در راه رفتن یا کنترل تعادل بطوریکه بیش از یک هفته طول بکشد بوده اید ؟<br>□۱- بلی□ ۲- خیر	□
C41	آیا شما بیش از یک بار بطور موقت و بدون دلیل خاصی و برای مدت کوتاهی بیهوش شده یا غش کرده اید؟<br>□ ۱- بلی□ ۲- خیر	□
C42	آیا شما هرگز دچار اختلال در تفکر، حافظه و یا تکلم بطوریکه بیش از یک هفته طول بکشد شده اید؟<br>□۱- بلی□ ۲- خیر	□
C43	آیا شما هرگز دچار اختلال در بینایی یا دوبینی بطوریکه بیش از یک هفته طول بکشد و خودبخود بهبود یابد شده اید؟ □۱- بلی□ ۲- خیر	□
C44	آیا هرگز دچار ضعف عضلانی در هیچ قسمت بدن بطوریکه بیش از یک هفته طول بکشد شده اید؟<br>□۱- بلی□ ۲- خیر	□
C45	آیا هرگز دچار هیچگونه اختلال در حرکت (کندی و سفتی اندامها)، لرزش دست یا پا که بیش از یک هفته طول بکشد شده اید؟<br>□۱- بلی□ ۲- خیر	□
C46	آیا هرگز در پا ها یا دستهای خود دچار بی حسی یا گزگز بطوریکه بیش از یک هفته طول بکشد شده اید؟<br>□۱- بلی□ ۲- خیر	□
C47	آیا هرگز دچار ضربه به سر بطوریکه برای مدتی هر چند کم بی هوش شوید شده اید؟<br>□۱- بلی□ ۲- خیر	□
C48	آیا هرگز دچار حملات تکرارشونده سردرد که هر حمله حداقل ۴ ساعت طول بکشد (حداقل ۲ بار) شده اید؟<br>□۱- بلی□ ۲- خیر	□
C49	آیا هرگز دچار حملات سرگیجه (احساس دوران) که باعث اختلال جدی در عملکرد روزانه شود شده اید؟<br>□۱- بلی□ ۲- خیر	□
C50	آیا هرگز دچار حملات وزوز گوش که بیش از یک هفته تداوم یابد شده اید؟<br>□۱- بلی□ ۲- خیر	□
C51	آیا در ۵ سال اخیر هرگونه شکستگی داشته اید؟ □ ۱- بلی محل_______________ □ ۲- خیر	□
C52	در چه سنی آخرین بار شکستگی روی داد؟ □□	□□
C53	آیا آخرین شکستگی بخاطر افتادن بوده است؟ ۱- بلی ۲- خیر	□
C54	در طی سال گذشته چند بار زمین خورده اید؟ □□	□□
C55	آیا تا بحال شکستگی لگن یا فمور داشته اید؟ ۱- بلی ۲- خیر	□
C56	آیا تا بحال تشخیص استئوپورز (پوکی استخوان) داشته و یا پزشک به شما گفته است که درخطرآن هستید؟<br>□۱- بلی□ ۲- خیر	□
C57	آیا هرگز دچار کمر دردی که بیش از یک هفته طول بکشد و در کار روزمره اختلال جدی ایجاد کند، شده اید؟<br>□۱- بلی□ ۲- خیر	□
C58	آیا سابقه کمردرد با خشکی صبحگاهی بیشتر از یک ساعت دارید؟ □۱- بلی□ ۲- خیر	□

C59	آیا سابقه درد مفصل دارید؟ ☐۱- بلی☐ ۲- خیر	☐
C60	آیا سابقه درد مفصل با خشکی صبحگاهی بیشتر از یک ساعت دارید؟ ☐۱- بلی☐ ۲- خیر	☐
C61	آیا سابقه آفت مکرر دهان داشته اید؟ ☐۱- بلی☐ ۲- خیر	☐
C62	آیا سابقه آفت مکرر تناسلی داشته اید؟ ☐۱- بلی☐ ۲- خیر	☐
C63	آیا تشخیص روماتیسم مفصلی توسط پزشک داشته اید؟ ☐۱- بلی☐ ۲- خیر	☐

**شماره**	**مداخلات**	**۱- بلی<br>۲- خیر<br>۳- نمی دانم**	**تعداد دفعات**	**کد**
C64	سابقه جراحی		☐☐	
C65	سابقه بستری در بیمارستان		☐☐	
C66	سابقه ترانسفیوژن		☐☐	
C67	سایر:			
C68	سایر:			
C69	سایر:			

D. سوالات مربوط به داروهای مصرفی

**سؤالات و گزینه‌ها<br>دارو**	**۱-بلی<br>۲-خیر**	**مصرف :<br>۱) روزانه<br>۲) هفتگی<br>۳) ماهانه**	**تعداد دفعات مصرف:**	**دوز**	**مدت (سال)**
D1- داروهای قلبی ـ عروقی و ضد فشار خون<br>D1-1ـ آسپرین	☐	☐	☐☐	☐☐☐	☐☐
D1-2 ـ وارفارین / هپارین /پلاویکس	☐	☐	☐☐	☐☐☐	☐☐
D1-3 ـ دیورتیک ها	☐	☐	☐☐	☐☐☐	☐☐
D1-4ـ بتابلاکرها (آتنولول, پروپرانولول, متوپرولول)	☐	☐	☐☐	☐☐☐	☐☐
D1-5ـ ACEi (کاپتوپریل)	☐	☐	☐☐	☐☐☐	☐☐

سؤالات و گزینه‌ها<br>دارو	۱-بلی<br>۲-خیر	مصرف :<br>۱) روزانه<br>۲) هفتگی<br>۳) ماهانه	تعداد دفعات مصرف:	دوز	مدت (سال)
D1-6- دیگوکسین	☐	☐	☐☐	☐☐☐	☐☐
D1-7- ARB .(لوزارتان- والسارتان)	☐	☐	☐☐	☐☐☐	☐☐
D1-8- CCBs	☐	☐	☐☐	☐☐☐	☐☐
D1-9 – نیتروکانتین/TNG	☐	☐	☐☐	☐☐☐	☐☐
D2- داروهای درمان دیابت:<br>D2-1- انسولین	☐	☐	☐☐	☐☐☐	☐☐
D2-2- متفورمین	☐	☐	☐☐	☐☐☐	☐☐
D2-3- گلیبن کلامید	☐	☐	☐☐	☐☐☐	☐☐
D2-4- سایر	☐	☐	☐☐	☐☐☐	☐☐
D3- داروهای درمان هایپر لیپیدمی<br>D3-1ـ استاتین ها (آتوروستاتین و استاتین ها)	☐	☐	☐☐	☐☐☐	☐☐
D3-2ـ جم فیبروزیل	☐	☐	☐☐	☐☐☐	☐☐
D3-3ـ سایر	☐	☐	☐☐	☐☐☐	☐☐
D4- داروهای اعصاب و روان<br>D4-1ـ بنزودیازپین‌ها	☐	☐	☐☐	☐☐☐	☐☐
D4-2ـ والپروات سدیم	☐	☐	☐☐	☐☐☐	☐☐
D4-3ـ ضد تشنج ها	☐	☐	☐☐	☐☐☐	☐☐

سؤالات و گزینه‌ها<br>دارو	۱-بلی<br>۲-خیر	مصرف:<br>۱) روزانه<br>۲) هفتگی<br>۳) ماهانه:	تعداد دفعات مصرف:	دوز	مدت (سال)
D4-4ـ ضد افسردگی	☐	☐	☐☐	☐☐☐	☐☐
D4-5- ضد اضطراب	☐	☐	☐☐	☐☐☐	☐☐
D4-6- روانگردان	☐	☐	☐☐	☐☐☐	☐☐
D5- داروهای کورتیکواستروئیدی<br>D5-1- تزریقی	☐	☐	☐☐	☐☐☐	☐☐
D5-2- خوراکی	☐	☐	☐☐	☐☐☐	☐☐
D5-3- موضعی	☐	☐	☐☐	☐☐☐	☐☐
D6- دارو های گوارشی<br>D6-1- H2Blocker	☐	☐	☐☐	☐☐☐	☐☐
D6-2- PPI	☐	☐	☐☐	☐☐☐	☐☐
D6-3- Clidinium C	☐	☐	☐☐	☐☐☐	☐☐
D7- آنتی بیوتیک ها<br>D7-1- amoxicillin	☐	☐	☐☐	☐☐☐	☐☐
D7-2- Cefexim	☐	☐	☐☐	☐☐☐	☐☐
D7-3- Azithromycin	☐	☐	☐☐	☐☐☐	☐☐
D7-4- Metronidazole	☐	☐	☐☐	☐☐☐	☐☐
D7-5- سایر_______________	☐	☐	☐☐	☐☐☐	☐☐
D8- متفرقه<br>D8-1- استامینوفن	☐	☐	☐☐	☐☐☐	☐☐
D8-2-استامینوفن کدئین	☐	☐	☐☐	☐☐☐	☐☐

سؤالات و گزینه‌ها<br>دارو	۱-بلی<br>۲-خیر	مصرف :<br>۱) روزانه<br>۲) هفتگی<br>۳) ماهانه	تعداد دفعات مصرف:	دوز	مدت (سال)
D8-3-ایبوبروفن	☐	☐	☐☐	☐☐☐	☐☐
D8-4- دیکلوفناک	☐	☐	☐☐	☐☐☐	☐☐
Levothyroxine -D8-5	☐	☐	☐☐	☐☐☐	☐☐
MTX -D8-6	☐	☐	☐☐	☐☐☐	☐☐
Hydroxychloroquine -D8-7	☐	☐	☐☐	☐☐☐	☐☐
Sulfasalazine -D8-8	☐	☐	☐☐	☐☐☐	☐☐
D9- سایر (نام ببرید)	☐	☐	☐☐	☐☐☐	☐☐
D10	☐	☐	☐☐	☐☐☐	☐☐
D11	☐	☐	☐☐	☐☐☐	☐☐
D12	☐	☐	☐☐	☐☐☐	☐☐

FH . سابقه خانوادگی بیماریها

کد نسبت فامیلی: (۱) پدر، (۲) مادر، (۳) برادر تنی، (۴) خواهر تنی، (۵) برادر ناتنی، (۶) خواهر ناتنی، (۷) پسر، (۸) دختر (۹) پدربزرگ یا مادربزرگ، (۱۰) همسر، (۱۱) سایر خویشاوندان خونی درجه دو (عمو، عمه, دایی، خاله، عمو و عمه‌زاده، خاله و دایی‌زاده)

نام بیماری	کد:<br>۱☐- بلی<br>۲☐- خیر<br>۳☐- نمی دانم	نسبت فامیلی<br>در صورت داشتن بیش از یک فرد مبتلا کد مربوط در هر خانه تا ۵ نفر درج گردد.
FH1ـ دیابت	☐	☐/☐/☐/☐/☐
FH2ـ فشار خون	☐	☐/☐/☐/☐/☐
FH3ـ بیماریهای ایسمیک قلب (آنژین/نارسایی)	☐	☐/☐/☐/☐/☐
FH4 سکته قلبی	☐	☐/☐/☐/☐/☐
FHـ سکته مغزی	☐	☐/☐/☐/☐/☐
FH5ـ سابقه ابتلا به سرطان	☐	☐/☐/☐/☐/☐

۱- سرطان معده<br>۲- سرطان کولورکتال<br>۳- سرطان پستان<br>۴- سرطان پروستات		
FH6- سابقه صرع	☐	☐/☐/☐/☐/☐
FH7- سابقه اختلالات روانپزشکی	☐	☐/☐/☐/☐/☐
FH8- سابقه سردرد مزمن و راجعه	☐	☐/☐/☐/☐/☐
FH9- سابقه آلزایمر	☐	☐/☐/☐/☐/☐
FH10- آیا سابقه شکستگی لگن یا فمور	☐	☐/☐/☐/☐/☐
FH11- سایر<br>۱-<br>۲-<br>۳-	☐	☐/☐/☐/☐/☐

BP. اندازه گیری فشار خون

با دو بار اندازه گیری تأیید شود (برحسب میلیمتر جیوه)<br>BP1– بازوی راست : BP-1-1- دیاستولیک /سیستولیک |__||__||__| / |__||__||__| BP-1-2- دیاستولیک /سیستولیک |__||__||__| / |__||__||__|
BP2- بازوی چپ: BP-2-1 - دیاستولیک /سیستولیک |__||__||__| / |__||__||__| BP2-2 - دیاستولیک /سیستولیک |__||__||__| / |__||__||__|
PR1- تعداد ضربان قلب در حالت استراحت: (یک دقیقه) |__| |__||__| PR2- تعداد ضربان قلب در حالت استراحت: (یک دقیقه) |__| |__||__|

OH. بهداشت دهان ودندان

OH1- دفعات مسواک زدن دندانها:<br>۱- یکبار در روز ۲- دو بار در روز ۳- سه بار در روز<br>۴- سایر ۵- مسواک نمی زنم	☐
OH2- معیار DMF<br>OH-2-1- تعداد کل دندانها |__||__| OH-2-2 - تعداد دندانهای پوسیده (D) |__||__| OH-2-3 - تعداد دندانهای کشیده شده (M) |__||__| OH-2-4- تعداد دندانهای پر شده (F) |__||__|	☐☐<br>☐☐<br>☐☐<br>☐☐
OH3-آیا ضایعه یا زخمی در دهان وجود دارد؟ ☐ ۱- بلی ☐ ۲- خیر	☐
OH4- آیا از نخ دندان استفاده میکنید؟ ☐ ۱- بلی - چند بار در هفته؟ ______ ☐ ۲- خیر	☐
OH5- آیا دندان مصنوعی دارید؟ ☐ ۱- بلی ☐ ۲- خیر	
OH6- آیا از دهان شوی استفاده میکنید؟ ☐ ۱- بلی - چند بار در هفته؟ ______ ☐ ۲- خیر	

H- سوالات مربوط به عادات فردی(مصرف الکل و دخانیات)

سؤالات و گزینه‌ها	کد
H1- آیا شما در طول زندگیتان حداقل ۱۰۰ نخ سیگار کشیده اید؟<br>☐ ۱- بلی ، سوالات بعدی پرسیده شود. ☐۲- خیر از H8-1 سوال ادامه دهید. ☐ ۳- مطمین نیستم	☐
H2- اولین سیگار را در چه سنی شروع کردید؟ سن به سال ☐☐	☐☐
H3- شما سیگار را بطور منظم از چه سنی شروع کردید؟ سن به سال ☐☐	☐☐
H4- آیا شما هم اکنون سیگار می کشید؟<br>☐۱- بلی روزانه ☐۲- گاهی اوقات ۳.خیر	☐
H5- بطور متوسط چند بار در شبانه روز سیگار میکشیده یا میکشید ؟☐☐	☐☐
H6* - از چه سنی سیگار کشیدن روزانه را متوقف نموده اید؟☐☐	☐☐

H7- مصرف سیگار را از زمان آغاز مصرف فهرست نمایید:

از سن	تا سن	نوع •	تعداد در روز	روز در هفته
		|__|	|__||__|	|__|
		|__|	|__||__|	|__|
		|__|	|__||__|	|__|
		|__|	|__||__|	|__|
		|__|	|__||__|	|__|

۱- سیگار کارخانه ای ۲- سیگار دست ساز ۳- سیگار برگ

سؤالات و گزینه‌ها	کد
H8-1- آیا شما در معرض تماس با دود سیگار در منزل بوده/هستید؟ ☐ ۱- بلی ....ساعت در روز ☐ ۲- خیر<br>H8-2-چند ساعت در روز در تماس با دود سیگار در محل کارتان هستید؟<br>۱)من در خارج از منزل کار نمی کنم (کدد ۹۹) ۲)تقریبا هرگز (صفر درج گردد) ۳) ☐☐ ساعت در روز	☐
H8-3- آیا فردی در خا نوا ده تان در دوران کودکی تان سیگار می کشیده است؟☐ ۱- بلی ☐ ۲- خیر	☐

H8– آیا تاکنون ناس ، قلیان ، چپق یا پیپ استفاده کرده اید؟

☐ ۱- بلی ☐ ۲- خیر

☐

نوع مصرف	از سن	تا سن	دفعات معمول روزانه	روز در هفته	توضیحات
H8-1- چپق	☐☐	☐☐	☐☐	☐	
H8-2- پیپ	☐☐	☐☐	☐☐	☐	
H8-3- قلیان	☐☐	☐☐	☐☐	☐	
H8-4- ناس	☐☐	☐☐	☐☐	☐	

H9- آیا تا کنون ازمواد مخدر استفاده کرده اید؟ ☐ ۱- بلی ☐ ۲- خیر

(نحوه مصرف: ۱- خوراکی ۲- استنشاقی ۳- تزریقی)

نوع مخدر	نحوه مصرف	سن مصرف	دفعات معمول روزانه	روز در هفته
H9-1- تریاک	☐	از☐☐ تا ☐☐	☐☐	☐
H9-2- هرویین	☐	از☐☐ تا ☐☐	☐☐	☐
H9-3- سوخته	☐	از☐☐ تا ☐☐	☐☐	☐
H9-4 - شیره	☐	از☐☐ تا ☐☐	☐☐	☐
H9-5– شیشه	☐	از☐☐ تا ☐☐	☐☐	☐
H9-6 – کوکایین	☐	از☐☐ تا ☐☐	☐☐	☐
H9-7- کراک	☐	از☐☐ تا ☐☐	☐☐	☐
H9-8– کریستال	☐	از☐☐ تا ☐☐	☐☐	☐
H9-9- پان	☐	از☐☐ تا ☐☐	☐☐	☐
H9-10- سایر	☐	از☐☐ تا ☐☐	☐☐	☐

H-9- آیا تاکنون از مشروبات الکلی استفاده کرده‌اید؟ ☐ ۱- بلی ☐ ۲- خیر

PERSIAN Cohort

نوع	☐ ۱- بلی ☐ ۲- خیر	از سن	تا سن	متوسط میزان مصرف در هر بار مصرف (cc)	تعداد دفعات مصرف در ماه	توضیحات
H9-1- آبجو (۷-۵٪الکل)	☐	☐☐	☐☐		☐☐	
H9-2- مشروبات با الکل<۴۰٪ (ودکا، ویسکی، جین وغیره)	☐	☐☐	☐☐		☐☐	

H9-3- مشروبات دست‌ساز	☐	☐ ☐	☐ ☐		☐ ☐	
H9-4- سایر	☐	☐ ☐	☐ ☐		☐ ☐	

PE.معاینه فیزیکی و معلولیت های جسمی

PE1- طاسی سر<br>PE2- نوع طاسی:	☐ ۱- دارد<br>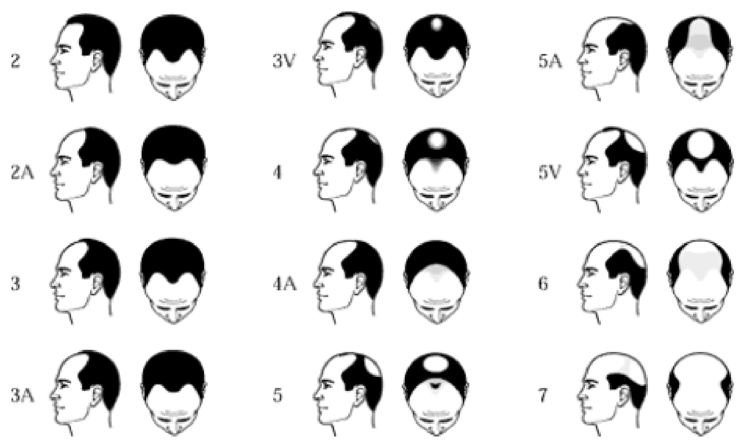 	☐ ۲- ندارد	☐<br>☐☐
PE3- موی زاید صورت (در زنان)	☐ ۱- دارد	☐ ۲- ندارد	☐
PE5- رنگ عنبیه	۱. قهوه ای/ میشی<br>۲. عسلی<br>۳. سبز<br>۴. آبی/ خاکستری		
PE6- قطع/ فلجی اندام:	☐ ۱- دارد (سوال های زیر را پاسخ دهید)	☐ ۲- ندارد	☐
PE7- اندام های درگیر	۱. فوقانی راست<br>۲. فوقانی چپ<br>۳. تحتانی راست<br>۴. تحتانی چپ<br>۵. انگشت/های دست<br>۶. انگشت/ های پا<br>۷. چشم<br>۸. گوش		☐/☐
PE8- علت:	۱. مادرزادی<br>۲. جنگی		☐/☐

	۳. شغلی/ حوادث<br>۴. به علت بیماری (دیابت)		
PE9- اختلالات ستون فقرات	☐ ۱- دارد (سوال های زیر را پاسخ دهید)	☐ ۲- ندارد	☐
PE10- نوع:	۱. اسکولیوز (کژپشتی)<br>۲. لوردوز (کاوپشتی)<br>۳. کیفوز (گوژپشتی)	☐	☐

CR. خواب

CR1 - معمولاً شبها چه ساعتی خوابتان می برد؟ |__||__| CR2- معمولاً صبحها چه ساعتی از خواب بیدار می شوید؟ |__||__| CR3- دوست دارید صبحها چه ساعتی ازخواب بیدار شوید؟ |__||__| CR4- آیا در طول روز (سه بار یا بیشتر در هفته) هم می خو ابید؟<br>☐۱- بلی ☐ ۲- خیر اگر بلی ،هر بار چند دقیقه؟ |__||__||__| CR5- آیا در طول یکسال گذشته کارشبانه (حداقل ۶ ساعت در فاصله ۹ شب تا ۶ صبح) داشته اید؟<br>☐۱- بلی ☐ ۲- خیر اگربلی، چند شب در ماه ؟ |__||__| CR6- آیا در طول خواب پاهایتان زیاد حرکت می کند بطوریکه فرد کنار شما ممکن است از خواب بیدار شود؟<br>☐۱- بلی ☐۲- خیر ☐۳- نمیدانم<br>CR7- آیا در طول روز وقتی فعالیتی ندارید بدون اختیار چرت می زنید؟ ☐ ۱- بلی ☐ ۲- خیر<br>CR-8- آیا از دارو های خواب آور بطور مداوم (بیشتر از ۲ بار در هفته) استفاده میکنید؟ ☐ ۱- بلی ☐ ۲- خیر	|__||__
__		__
__||__| |__||__||__| |__| |__| |__|

P. پرسشنامه فعالیت فیزیکی

این فرد دارای الگوی فعالیت فیزیکی ثابت / متفاوت در طول سال است.

دسته بندی	نوع فعالیت فیزیکی	شکل	دقیقه / ساعت
PA1	در طول شبانه روز، خواب شبانه شما، بطور متوسط چقدر است؟	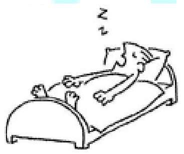	|__||__|/|__||__|
PA2	در طول ۲۴ ساعت، خواب عصر هنگام یا بین روز شما چقدر است؟		|__||__|/|__||__|
PA3	در طول شبانه روز، اگر مدت زمان بیش از ۱۰ دقیقه، در خواب نیستید، اما در حالت دراز کشیده (بر روی تخت، رختخواب یا زمین) استراحت مینمایید، آنرا نیز مشخص نمایید؟		|__||__|/|__||__|
PB1	در طول شبانه روز، چه مدت زمانی را به تماشای تلویزیون، گوش دادن موسیقی، تماشای فیلم با کامپیوتر و موارد مشابه، مشغول هستید؟	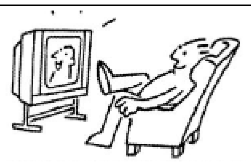	|__||__|/|__||__|
PB2	در طول شبانه روز، چه مدت زمانی را به مطالعه کتاب، مجله، روزنامه و یا موارد مشابه، مشغول هستید؟		|__||__|/|__||__|
PC1	در طول شبانه روز، چه مدت زمانی را به انجام کار اداری پشت میز و نشسته، مشغول هستید؟	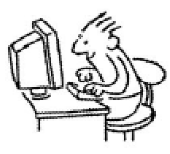	|__||__|/|__||__|
PC2	در طول شبانه روز، چه مدت زمانی را به کار با رایانه (پشت میز یا هر وضعیت دیگری)، مشغول هستید؟		|__||__|/|__||__|

<table>
<tr><td>PC3</td><td>در طول شبانه روز، چه مدت زمانی را به غذا خوردن، نشستن در جلسات، میهمانیها، همایشها و مشابه آن، مشغول هستید؟</td><td></td><td>|__||__|/|__||__|</td></tr>
<tr><td>PD1</td><td>در طول شبانه روز، چه مدت زمانی را به آشپزی، طبخ انواع غذاها، شستن ظرفها، فعالیتهای ایستاده و مشابه آن، مشغول هستید؟</td><td rowspan="2">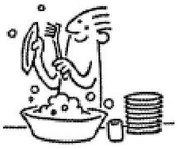</td><td>|__||__|/|__||__|</td></tr>
<tr><td>PD2</td><td>در طول شبانه روز، چه مدت زمانی را به رانندگی (موتور، خودروی سبک یا سنگین)، کارهای اپراتوری نشسته (با فعالیت فیزیکی مشابه رانندگی) و مشابه آنها، مشغول هستید؟</td><td>|__||__|/|__||__|</td></tr>
<tr><td>PE1</td><td>در طول شبانه روز، چه مدت زمانی را به کارهای سبک نظافت منزل، شستن انواع البسه، جارو کردن و مشابه آن، مشغول هستید؟</td><td rowspan="3">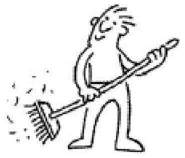</td><td>|__||__|/|__||__|</td></tr>
<tr><td>PE2</td><td>در طول شبانه روز، آیا مدت زمانی را به انجام کارهایی مانند فروشندگی مواد غذایی سیار، هر نوع فروشندگی در حد آن یا مشابه آن، مشغول هستید؟ اگر بلی، چه مدت زمانی؟</td><td>|__||__|/|__||__|</td></tr>
<tr><td>PE3</td><td>در طول شبانه روز، چه مدت زمانی را به پیاده روی یا راه رفتن آهسته، پایین رفتن از پله، حرکات ورزشی یا حرکات موزون آرام و مشابه آن، مشغول هستید؟</td><td>|__||__|/|__||__|</td></tr>
<tr><td>PF1</td><td>در طول شبانه روز، آیا مدت زمانی را به پیاده روی سریع، انجام ورزش ائوربیک سبک، دوچرخه سواری تفریحی، دوچرخه سواری بمنظور تردد بین منزل تا محل کار یا مشابه آن، مشغول هستید؟ اگر بلی، چه مدت زمانی؟</td><td rowspan="4">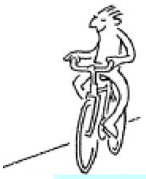</td><td>|__||__|/|__||__|</td></tr>
<tr><td>PF2</td><td>در طول شبانه روز، آیا مدت زمانی را به انجام کارهایی مانند رانندگی ادوات کشاورزی، ماشین آلات راهسازی، یا هر نوع فعالیت مشابه، مشغول هستید؟ اگر بلی، چه مدت زمانی؟</td><td>|__||__|/|__||__|</td></tr>
<tr><td>PF3</td><td>آیا در طول شبانه روز، مدت زمانی را به مشاغل فنی سبک (تعویض روغن، سرویس خودرو، کارواش، خدمات رنگ خودرو، باتریسازی و ...) مشغولید؟ اگر بلی، چه مدت زمانی؟</td><td>|__||__|/|__||__|</td></tr>
<tr><td>PF4</td><td>در طول شبانه روز، آیا به انجام کارهایی مانند مشاغل بنایی سبک (گچکاری، نقاشی، کاشیکاری)، نجاری یا هر نوع فعالیت مشابه، مشغول هستید؟ اگر بلی، چه مدت زمانی؟</td><td>|__||__|/|__||__|</td></tr>
<tr><td>PG1</td><td>در طول شبانه روز، آیا زمانی را به انجام کارهایی مانند حمل اسباب و اثاثیه، حمل اشیاء سبک از پله ها، یا هر نوع فعالیت مشابه، مشغول هستید؟ اگر بلی، چه مدت زمانی؟</td><td rowspan="3">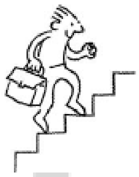</td><td>|__||__|/|__||__|</td></tr>
<tr><td>PG2</td><td>در طول شبانه روز، آیا زمانی را به انجام کارهایی مانند باغبانی، فعالیتهای کشاورزی سبک، یا هر نوع فعالیت مشابه، مشغول هستید؟ اگر بلی، چه مدت زمانی؟</td><td>|__||__|/|__||__|</td></tr>
<tr><td>PG3</td><td>در طول شبانه روز، آیا زمانی را به انجام مشاغلی مانند مشاغل فنی سنگین (خدمات موتور خودرو، جلوبندی، آهنگری، تراشکاری، ریخته گری و ...)کار در کارگاههای چوب بری، یا هر نوع فعالیت مشابه، مشغول هستید؟ اگر بلی، چه مدت زمانی؟</td><td>|__||__|/|__||__|</td></tr>
<tr><td>PH1</td><td>در طول شبانه روز، آیا زمانی را به انجام فعالیتهای کارگری یا کشاورزی سنگین (توام با یکی از موارد بیل زدن، تبر زدن، پتک زدن، چکش زدن)، پارو کردن برف، حمل اشیاء سنگین از پله ها یا هر نوع فعالیت مشابه، مشغول هستید؟ اگر بلی، چه مدت زمانی؟</td><td rowspan="2">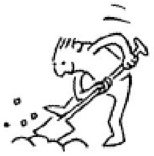</td><td>|__||__|/|__||__|</td></tr>
<tr><td>PH2</td><td>در طول شبانه روز، آیا زمانی را به انجام ورزشهایی مانند بدنسازی، ورزش ائوربیک سنگین یا هر نوع فعالیت مشابه، مشغول هستید؟ اگر بلی، چه مدت زمانی؟</td><td>|__||__|/|__||__|</td></tr>
<tr><td>PI</td><td>چنانچه در طول یک هفته، ورزش انجام میدهد، ابتدا کد ۵ رقمی آنرا مشخص</td><td>|__||__||__||__||__|</td><td></td></tr>
</table>

<table dir="rtl">
<tr><td></td><td>نموده، سپس بطور متوسط، مدت زمان آن ورزش در هفته را نیز مشـخص نماییـد (حداکثر تا ۳ ورزش):
نام ورزش ۱:..........................
نام ورزش ۲:..........................
نام ورزش ۳:..........................</td><td>|__||__||__||__||__|
|__||__||__||__||__|</td><td>|__||__|/|__||__|
|__||__|/|__||__|
|__||__|/|__||__|</td></tr>
<tr><td></td><td colspan="2">جمع ساعات در شبانه روز</td><td>۲۴/ ۰۰</td></tr>
</table>

E. معاینه آنتروپومتریک

<table dir="rtl">
<tr><td colspan="2">وزن و قد باید بدون کفش ولباسهای سنگین اندازه گیری شود.</td></tr>
<tr><td>E1- قد به سانتیمتر</td><td>|__||__||__|</td></tr>
<tr><td>E2- وزن به کیلوگرم</td><td>|__||__||__|</td></tr>
<tr><td>E3- دور شکم به سانتیمتر</td><td>|__||__||__|</td></tr>
<tr><td>E4- دور باسن به سانتیمتر</td><td>|__||__||__|</td></tr>
<tr><td>E5-دور مچ به سانتیمتر</td><td>|__||__|</td></tr>
</table>

PERSIAN Cohort

F. پرسشنامه بسامد مصرف خوراک

ردیف	مواد غذایی	مقدار	متوسط بار مصرف در سال گذشته				میزان مصرف هر بار	ماه در سال	ملاحظات
			روز	هفته	ماه	سال			
**F1**	**نان و غلات**								
F۱-1	**نان لواش**	کف دست / کامل							
F1-۲	**نان بربری/تافتون**	کف دست / کامل							
F1-۳	**نان سنگک**	کف دست / کامل							
F1-۴	**نان باگت و فانتزی**	یک عدد							۱. با سبوس<br>۲. سفید
F1-۵	**برنج پخته**	کفگیر / بشقاب							
F1-6	**ماکارونی پخته/لازانیا**	کفگیر / بشقاب							
F1-7	**جو پخته/بلغور**	قاشق غذاخوری							
F1-8	**گندم پخته**	قاشق غذاخوری							
F1-9	**نان تیری**	یک عدد							

ردیف	مواد غذایی	مقدار	متوسط بار مصرف در سال گذشته				میزان مصرف هر بار	ماه در سال	ملاحظات
			روز	هفته	ماه	سال			
**F2 حبوبات**									
F2-۱	لوبیا	قاشق غذاخوری							
F2-2	نخود	قاشق غذاخوری							
F2-3	ماش/ عدس / دال عدس	قاشق غذاخوری							
F2-4	لپه	قاشق غذاخوری							
F2-5	سویا (پروتئین سویا، دانه سویا)	قاشق غذاخوری							
F2-6	باقالی پخته	قاشق غذاخوری							
F2-7									
**F3 گوشت و فرآورده های آن**									
F3-۱	گوشت قرمز (آبگوشتی، خورشتی، چرخ کرده، کبابی)	قوطی کبریت							۱. کم چرب<br>۲. پر چرب
F3-۲	گوشت مرغ	قوطی کبریت							۱. با پوست<br>۲. بدون پوست
F3-۳	سایر قسمتهای مرغ (جگر، دل، سنگدان)	قوطی کبریت							۱. سرخ شده<br>۲. آ ب پز<br>۳. کبابی
F3-۴	تخم مرغ	عدد							
F3-۵	ماهی	قطعه متوسط							

ردیف	مواد غذایی	مقدار	متوسط بار مصرف در سال گذشته				میزان مصرف هر بار	ماه در سال	ملاحظات
			روز	هفته	ماه	سال			
F3-۶	تن ماهی (کنسرو)	قاشق غذاخوری							۱. با روغن<br>۲. بدون روغن
F3-۷	سوسیس، کالباس	یک واحد							
F3-۸	کباب لقمه / همبرگر	یک واحد							
F3-۹	دل ، جگر و قلوه (گوسفند،گوساله و..)	قوطی کبریت							۱. کبابی<br>۲. سرخ شده<br>۳. آب پز
F3-۱۰	مغز	عدد							
F3-۱۱	زبان گوسفند	عدد							
F3-۱۲	کله، پاچه /سیرابی /شیردان	بشقاب غذاخوری							
F3-۱۳	پیتزا	برش							
F3-14	گوشت ماکیان (غیر از مرغ)	قوطی کبریت							
F4 شیر و لبنیات									
F4-۱	شیر	لیوان							۱. کم چرب<br>۲. پرچرب /محلی<br>۳. شیر کاکائو
F4-۲	ماست	لیوان							۱. کم چرب<br>۲. پرچرب/ محلی<br>۳. خامه ای<br>۴. چکیده
F4-۳	پنیر	قوطی کبریت							۱. معمولی<br>۲. خامه ای<br>۳. محلی

ردیف	مواد غذایی	مقدار	متوسط بار مصرف در سال گذشته				میزان مصرف هر بار	ماه در سال	ملاحظات
			روز	هفته	ماه	سال			
F4-4	**پنیر پیتزا**	قاشق غذاخوری							
F4-5	**دوغ**	لیوان							
F4-6	**کشک**	قاشق غذاخوری							
F4-7	**آغوز**	لیوان							
**F5 سبزی ها**									
F5-۱	**کاهو خردشده**	۱ لیوان/ پیش دستی							
F5-۲	**کلم (شامل کلم سفید، قرمز، کلم قمری، گل کلم و کلم بروکلی)**	۱ لیوان							
F5-۳	**گوجه فرنگی**	عدد متوسط							
F5-۴	**خیار**	عدد متوسط							
F5-۵	**سبزی خوردن**	ا لیوان/ پیش دستی							
F5-۶	**سبزی خورشتی/آش/ کوکو/دلمه/اسفناج**	پیش دستی / لیوان							
F5-۷	**کدو / بادمجان خورشتی**	عدد متوسط							
F5-۸	**کرفس خام یا پخته / کنگر**	۱ لیوان/ پیش دستی							
F5-۹	**چغندر (لبو)/شلغم**	عدد متوسط							
F5-۱۰	**سیب زمینی** (آب پز و سرخ کرده)	عدد متوسط							

ردیف	مواد غذایی	مقدار	متوسط بار مصرف در سال گذشته				میزان مصرف هر بار	ماه در سال	ملاحظات
			روز	هفته	ماه	سال			
F5-۱۱	هویج	عدد متوسط							۱. خام<br>۲. پخته<br>۳. سرخ شده<br>۴. آب هویج
F5-۱۲	سیر	حبه							
F5-۱۳	پیاز	عدد متوسط							۱. پیاز داغ<br>۲. پیاز پخته<br>۳. پیاز خام
F5-14	فلفل دلمه ای / فلفل سبز	۱ لیوان/ پیش دستی							
F5-15	قارچ پخته	قاشق غذاخوری							
F5-16	ذرت و بلال	۱ لیوان / ۱ عدد							
F5-17	نخود سبز	قاشق غذاخوری							
F5-18	لوبیا سبز	قاشق غذاخوری							
F5-19	کدو حلوایی	قطعه ۶x۶							
F5-20	سبزیجات محلی	پیش دستی / لیوان							
F5-21	آبلیمو / آبغوره / آب نارنج	قاشق غذاخوری							

ردیف	مواد غذایی	مقدار	متوسط بار مصرف در سال گذشته				میزان مصرف هر بار	ماه در سال	ملاحظات
			روز	هفته	ماه	سال			
**F6 میوه ها**									
**F6-۱**	**طالبی و گرمک (شمام)**	قاچ متوسط							
**F6-2**	**خربزه**	قاچ متوسط							
**F6-3**	**هندوانه**	قاچ متوسط							
**F6-4**	**زردآلو**	عدد متوسط							
**F6-5**	**گیلاس / آلبالو**	پیش دستی							
**F6-6**	**هلو/شلیل/شفتالو**	عدد							
**F6-7**	**گوجه سبز**	عدد متوسط							
**F6-8**	**انواع توت تازه** (سفید، قرمز، شاه توت)	پیش دستی							
**F6-9**	**توت فرنگی**	عدد متوسط							
**F6-10**	**آلو** (زرد و قرمز)	عدد متوسط							
**F6-11**	**انجیر تازه**	عدد متوسط							
**F6-12**	**انگور**	خوشه متوسط							
**F6-13**	**گلابی**	عدد متوسط							
**F6-14**	**سیب**	عدد متوسط							۱. با پوست<br>۲. بدون پوست
**F6-15**	**کیوی**	عدد متوسط							
**F6-16**	**مرکبات** (پرتقال، نارنگی، لیمو ترش، لیموشیرین، گریپ فروت)	عدد متوسط							

PERSIAN Cohort

ردیف	مواد غذایی	مقدار	متوسط بار مصرف در سال گذشته				میزان مصرف هر بار	ماه در سال	ملاحظات
			روز	هفته	ماه	سال			
F6-17	انار	عدد متوسط							
F6-18	موز	عدد متوسط							
F6-19	خرمالو	عدد متوسط							
F6-20	خرما	عدد							
F6-21	آبمیوه طبیعی	۱ لیوان							۱. سیب<br>۲. پرتقال<br>۳. طالبی<br>۴. سایر موارد
F6-22	خشکبار (انجیر ، هلو، آلو،زردآلو)	عدد متوسط							
F6-23	کشمش ، مویز ، توت	قاشق غذاخوری							
F6-24	انبه	عدد متوسط							
F6-25	چاغاله بادام	۵ عدد متوسط							
F7	انواع روغن، دانه های روغنی و کره								
F7-۱	مارگارین / کره گیاهی	قاشق مرباخوری							
F7-2	کره، خامه و سرشیر	قاشق مرباخوری							
F7-۳	روغن جامد/ نیمه جامد	قاشق غذاخوری							۱. گیاهی<br>۲. حیوانی<br>۳. دنبه
F7-۴	روغن مایع	قاشق غذاخوری							۱. کانولا/سویا<br>۲. سایر روغنهای مایع (آفتابگردان/ذرت)<br>۳. روغن سرخ کردنی

ردیف	مواد غذایی	مقدار	متوسط بار مصرف در سال گذشته				میزان مصرف هر بار	ماه در سال	ملاحظات
			روز	هفته	ماه	سال			
**F7**	**انواع روغن، دانه های روغنی و کره**								
**F7-۵**	**روغن زیتون**	قاشق غذاخوری							
**F7-۶**	**زیتون**	عدد							
**F7-۷**	**سس مایونز/سالاد**	قاشق غذاخوری							
**F7-۸**	**گردو**	عدد							
**F7-9**	**بادام زمینی**	عدد							
**F7-10**	**سایر مغزها (بادام / بادام هندی / پسته / فندوق)**	عدد							
**F7-11**	**مغز تخمه (کدو، آفتابگردان، هندوانه)**	قاشق غذاخوری							
**F7-12**	**بنه**	نصف لیوان							
**F8**	**قند ها**								
**F8-۱**	**قند / شکر**	حبه/ قاشق چای خوری							
**F8-2**	**نبات/ آبنبات / نقل / شکرپنیر**	حبه							
**F8-3**	**عسل**	قاشق مرباخوری							
**F8-4**	**مربا**	قاشق مرباخوری							
**F8-5**	**مسقطی**	قوطی کبریت							
**F9**	**متفرقه**								
**F9-1**	**چای**	**لیوان**							۱. کمرنگ<br>۲. معمولی<br>۳. پررنگ

ردیف	مواد غذایی	مقدار	متوسط بار مصرف در سال گذشته				میزان مصرف هر بار	ماه در سال	ملاحظات
			روز	هفته	ماه	سال			
F9-2	نوشابه	لیوان							
F9-3	ماءالشعیر	لیوان							
F9-4	قهوه / نسکافه	لیوان							
F9-5	بستنی (سنتی /غیر سنتی/کیم)	لیوان / عدد							
F9-6	شیرینی خشک / کیک خشک/ بیسکویت / سوهان	یک واحد							
F9-7	شیرینی خامه دار / کیک خامه دار	عدد/ برش							
F9-8	شکلات / شکلات صبحانه	عدد / قاشق غذاخوری							
F9-9	انواع چیپس	بسته متوسط							
F9-10	پفک	بسته متوسط							
F9-11	انواع حلوا (شکری،کنجد ،خانگی)	قاشق غذاخوری							
F9-12	خیارشور /شور	یک پیاله							
F9-13	انواع ترشی	یک پیاله							
F9-14	رب/سس گوجه فرنگی	قاشق غذاخوری							
F9-15	آبمیوه صنعتی (ساندیس، رانی و سایر)	قوطی / لیوان							
F9-16	گز	قوطی کبریت							
F9-17	سکنجبین	قاشق غذاخوری							

ردیف	مواد غذایی	مقدار	متوسط بار مصرف در سال گذشته				میزان مصرف هر بار	ماه در سال	ملاحظات
			روز	هفته	ماه	سال			
**F10** ادویه جات									
F10-۱	نمک	۱ قاشق چای خوری							
F10-2									
F10-3									

F11 جدول مکمل های غذایی (داده ها بر اساس تعداد وارد گردد نه بسته کامل قرص یا کپسول)

ردیف	مکمل ها		روز	هفته	ماه	سال
F11-۱	مولتی ویتامین و مینرال					
F11-۲	مولتی ویتامین					
F11-۳	کلسیم + ویتامین D					
F11-۴	کلسیم					
F11-۵	ویتامین D	قرص				
		آمپول				
F11-۶	اسید فولیک					
F11-۷	امگا ۳ / روغن ماهی					
F11-۸	آهن (فروسولفات / ففول)					
F11-۹	روی یا زینک (زینک سولفات / زینک گلوکونات)					
F11-۱۰	سایر ویتامین ها					

F12. مصرف آب

ردیف	مصرف آب در فصلهای مختلف سال	مقدار	روز	هفته	ماه
F12-۱	میزان آب مصرفی در تابستان	لیوان			

**F12-۲**	**میزان آب مصرفی در سایر فصلها**	**لیوان**			

F13 عادات غذایی

F13-1- روزانه شما غذای خود را در چند وعده می خورید؟<br>۱) ۳ وعده (صبحانه،نهار، شام) ۲) ۴ وعده (صبحانه، نهار، شام، میان وعده)<br>۳) ۶-۵ وعده (صبحانه، نهار، شام، ۳-۲ میان وعده) ۴) بیش از ۶ وعده ۵) کمتر از ۳ وعده	☐
F13-2آیا شما عادت به اضافه کردن نمک به غذا سر سفره دارید؟ ☐ بلی ☐بعضی مواقع ☐ خیر	☐
F13-3 - هر چند وقت یکبار غذاهای کبابی می خورید؟<br>۱)هرگز ۲)کمتر از یکبار در ماه ۳) ۳-۱ بار در ماه ۴) ۳-۱ بار در هفته<br>۵)روزانه	☐
F13-4 - هر چند وقت یکبار شما از غذاهای سرخ شده استفاده می کنید؟<br>۱) هرگز ۲) کمتر از یکبار در ماه ۳) ۳-۱ بار در ماه ۴) ۳-۱ بار در هفته ۵)<br>روزانه	☐
F13-5- نحوه سرخ کردن سیب زمینی، بادمجان، کدو، و پیاز چگونه می باشد؟<br>۱) تفت دادن ۲) طلایی شدن ۳) قهوه ای شدن	☐
F13-6 - نحوه سرخ کردن سبزیجات چگونه می باشد؟<br>۱) تفت دادن ۲) طلایی شدن ۳) قهوه ای شدن	☐
F13-7 -از چه نوع روغنی برای سرخ کردن مواد غذایی استفاده می کنید؟<br>۱)روغن جامد ۲)روغن نیمه جامد ۳)روغن مایع ۳) روغن مایع مخصوص سرخ کردن ۴)سایر روغن ها	
F13-8 - آیا از روغن های مانده پس از سرخ کردن یا پختن سایر غذاها دوباره استفاده می کنید؟<br>۱) بلی ۲) خیر	☐
F13-9 - در صورت مثبت بودن سوال قبلی، تا چند بار از آن استفاده می کنید؟<br>________بار	☐
F13-10 - اگر غذایی مثل رب، مربا، ترشی، آبغوره، و سرکه کپک زده بود، قسمتی از آن را برداشته و بقیه را مصرف می کنید یا کلا دور می رزید؟ ۱) مصرف می کنید ۲) دور می ریزید	☐
F13-11 - آیا شما از غذاهای دودی مثل برنج دودی و ماهی دودی استفاده می کنید؟<br>۱) هرگز ۲) کمتر از یکبار در ماه ۳) ۳-۱ بار در ماه ۴) ۳-۱ بار در هفته ۵) روزانه	☐
F13-12 - سبزیجات را به چه صورت نگهداری می کنید؟<br>۱) خشک شده ۲) یخچال ۳) فریزر	☐
F13-13 - در صورت نگهداری سبزیجات در یخچال یا فریزر ، آنها را به چه صورتی نگه می دارید؟<br>۱) خام ۲) آبپز شده ۳) سرخ شده	☐

F13-14 - مواد گوشتی را در یخچال یا فریزر به چه صورتی نگه می دارید ؟<br>۱) خام ۲) آبپز شده ۳) سرخ شده	☐

F13-15 - چای و قهوه را با چه دمایی می خورید؟<br>۱) داغ ۲) ولرم ۳) سرد	
F13-16 - سوپ، آش و سایر مواد غذایی آبکی و مایع را با چه دمایی می خورید؟<br>۱) داغ ۲) ولرم ۳) سرد	
F13-17 - از چه نوع ظروفی برای نگهداری آب استفاده می کنید؟<br>۱) پلاستیکی ۲) استیل ۳) چینی ۴) شیشه ای ۵) سایر موارد	
F13-18 - مواد غذایی را در چه ظروفی نگهداری می کنید؟ (۲ انتخاب)<br>۱) پلاستیکی ۲) استیل ۳) پارچه ۴) شیشه ای ۵) سایر موارد<br>F13-18-1نان<br>F13-18-2آبلیمو،آبغوره<br>F13-18-3رب وآب گوجه فرنگی<br>F13-18-4 ترشی و خیارشور	
F13-19 از چه نوع ظروفی برای پخت و پز استفاده می کنید؟ (۲-۳ انتخاب)<br>۱) مس ۲) آلومینیوم ۳) لعابی ۴) تفلون ۵)چدن<br>۶)استیل 7) پیرکس ۸) سایر موارد	
F13-20 آیا از ظروف تفلون که سطوح شان خراش دار است استفاده می کنید؟<br>۱) بلی ۲) خیر	
F13-21 در چه ظروفی غذا می خورید؟ (۲-۳ انتخاب)<br>۱) چینی ۲) آلومینیوم ۳) لعابی ۴) ملامین ۵) استیل<br>۶) پلاستیکی ۷)شیشه ای ۸)آرکوپال	
F13-22 غذاهای مانده را در چه ظروفی نگهداری می کنید؟ (۲-۳ انتخاب)<br>۱) مس ۲) چینی ۳) آلومینیوم ۴) لعابی<br>۵) ملامین ۶) استیل ۸) پلاستیکی ۹) شیشه ای	
F13-23 آیا نسبت به غذای خاصی آلرژی یا حساسیت دارید؟<br>________________	
F13-24 -آیا از دم کرده های//داروهای گیاهی ، عرقیجات و یا پودرهای گیاهی (مثل پونه ، گل گاوزبان و...) استفاده می کنید ؟<br>بلی--- خیر---<br>F13-25 در صورت مثبت بودن پاسخ بیشتر از چه نوع گیاهی و به چه دلیلی استفاده می کنید؟.نوع................<br>دلیل	

B. نمونه های بیولوژیک

B1- نمونه ادرار گرفته شد؟	۱) بلی	۲) خیر	☐
B2- نمونه بزاق گرفته شد؟	۱) بلی	۲) خیر	☐
B3- نمونه خون گرفته شد؟	۱) بلی	۲) خیر	☐
B4- نمونه مو گرفته شد؟	۱) بلی	۲) خیر	☐
B5 - نمونه ناخن گرفته شد؟	۱) بلی	۲) خیر	☐
B6- نمونه مدفوع گرفته شد؟	۱) بلی	۲) خیر	☐
