# Supplementary material for: Cohort profile: Bandar Kong prospective study of chronic non-communicable diseases
Source: PLoS One. 2022 May 12;17(5):e0265388. doi: 10.1371/journal.pone.0265388 (PMC9098057; doi:10.1371/journal.pone.0265388)
Supplement: Attachment — Submitted filename: Reponse to reviewers and editor.docx [file pone.0265388.s005.docx]

The Editor-in-chief

*PLOS ONE*

With respect,

Responses to the referee's comments:

Manuscript Number: PONE-D-20-32677

Title: Reply to Manuscript Number: PONE-D-20-32677Title: Cohort Profile: The Bandar Kong Prospective Cohort Study

Article Type: Original research paper (Cohort profile)

Thanks a lot for your critical and peer review of our manuscript (ms). Our manuscript has been accepted with revision. We have provided a point-by-point response to all comments from the editors and reviewers and have revised the manuscript accordingly.

Therefore, we resubmit our manuscript (ms) titled “**Cohort Profile**: **The Bandar Kong Prospective Cohort Study**”, authored by Azim Nejatizadeh *et al*. Efforts have been made to keep the text to minimum with inclusion of the most relevant references. We edited the text and made the corrections and needful modifications as per the reviewer comments. In fact, we shortened the manuscript drastically and made well-defined focus objectives.

We have incorporated the editorial corrections in the revised version.

**Responses to the** **Journal Requirements:**

**Requirement 1**: Please ensure that your manuscript meets PLOS ONE's style requirements, including those for file naming.

**Reply 1:** We appreciate your suggestion. Of course, we followed the journal’s style requirements, such as file naming and so on.

**Requirement 2**: We suggest you thoroughly copyedit your manuscript for language usage, spelling, and grammar.

**Reply 2:** Thanks for the valuable comment. We do agree with the point which you raised. We took your point into consideration and modified/copyedited the entire text meticulously, spelling and then corrected grammar errors. We kept the manuscript as short as possible. We are not averse to further modifications of the language or the text.

**Requirement 3:** Please include a copy of the questionnaire in the original language, as Supporting Information, or include a citation if it has been published previously.

**Reply 3:** We appreciate your suggestion. Earlier, we uploaded the English version of it as “S1 File. Questionnaire. (PDF)”. We take your point into consideration while submission of the manuscript and will add and upload a copy of original language of the questionnaire in Persian.

**Requirement 4:** In statistical methods, please clarify whether you corrected for multiple comparisons.

**Reply 4:** I would like to take your kind consideration to the point that multiple correction is used mainly in a group of conditions to correct experiment-wise error rate once applying multiple “t” tests or as a post-hoc procedure to correct the cluster-wise error rate subsequent to analysis of variance (ANOVA). Rather, that is an adjustment to restrict the state of false positive and to avoid data from mistakenly looking to be statistically significant. Therefore, we didn’t correct for multiple comparison, since this is a cohort profile and we only reported the main characteristics of the population.

**Requirement 5:** In your statistical analyses, please state whether you accounted for repeated measurements in your statistical models.

**Reply 5:** Thanks a lot for raising this issue. To the best of my knowledge, the prospective cohort studies mainly underpin investigating the relation between exposure measure at baseline and occurrence of the health outcome of interest throughout follow up period. Repeated measurements can be made of both exposures and outcomes. However, at the time being, we completed the enrollment phase and started follow up phases. Of note, the submitted manuscript is just the cohort profile of the ongoing study and now, we used the data on baseline for the cohort profile and therefore we didn’t perform repeated measurement analysis.

**Requirement 6:** We note that you have indicated that data from this study are available upon request. PLOS only allows data to be available upon request if there are legal or ethical restrictions on sharing data publicly.

**Reply 6:** Yes. As you have noted, we have incorporated a section referred to as “Access to the data” at the end of the manuscript which has been set according to the Journal instructions to the authors. The respective section has also appeared below for your kind consideration.

**Access to the data**

The study is a part of the PERSIAN Cohort Study, the kind applicants should follow the respective rules and the protocol of data sharing and scientific collaboration. In case of any application or query, you may kindly email the principle investigator. All interested investigators in Iran and worldwide would have free access to the data of this study, and necessary processes are available at the Cohort website to reproduce the project, participate in collaborative research projects, and use the data. Access to the data is available from corresponding cohort ([azimnejate@yahoo.com](mailto:azimnejate@yahoo.com)) or from [http://persiancohort.com/access](http://persiancohort.com/access/)

**Requirement 7:** In your revised cover letter, please address the following prompts:

a) If there are ethical or legal restrictions on sharing a de-identified data set, please explain them in detail (e.g., data contain potentially identifying or sensitive patient information) and who has imposed them (e.g., an ethics committee). Please also provide contact information for a data access committee, ethics committee, or other institutional body to which data requests may be sent.
b) If there are no restrictions, please upload the minimal anonymized data set necessary to replicate your study findings as either Supporting Information files or to a stable, public repository and provide us with the relevant URLs, DOIs, or accession numbers.

**Reply 7:** As we addressed the matter in “**Reply to** **Requirement 6**”, there are some legal restrictions on sharing a de-identified data set, if requested without prior permission. We emphasized that access to the data is available through corresponding author (Email: [azimnejate@yahoo.com](mailto:azimnejate@yahoo.com); Phone number of the cohort center: +98-7644235008) or via <http://persiancohort.com/access> , in order to get your request fulfilled. Furthermore, you communicate or contact the vice chancellery of Hormozgan university of medical sciences from <http://resv.hums.ac.ir/> for further administrative processing of your application.

**Requirement 8:** We note that Figure 1 in your submission contains map images which may be copyrighted.

**Reply 8:** In fact, the map, i.e. Figure 1, is a part of a detailed plan of Bandar Kong Municipality (<https://www.cityhallworldwide.com/iran-town-hall/bandar-kong-municipality>) designed and drawn by Hormozgan Road and Urban Development Administration, then approved by the Supreme Council of Architecture and Urban Planning of Iran (<https://www.mrud.ir/>). We described in “method and materials” section of the manuscript that the function and field activity of the cohort center was launched and coordinated through Bandar Kong Municipality as a main stakeholder and sponsor of the study. Of course, once the study started, the concerned authority provided us all the needful requirements including the maps and so on.

Regarding “Figure” representing the sites of PERSIAN Cohort as well as Kong Cohort Study across country ([http://persiancohort.com/access](http://persiancohort.com/access/)). In introduction of the manuscript, we explained that our study is a part of PERSIAN Cohort study, that is why, we incorporated this map from PERSIAN Cohort. If you are not satisfied with the justification, you may remove this figure.

**Requirement 9:** PLOS requires an ORCID iD for the corresponding author in Editorial Manager on papers submitted after December 6th, 2016. Please ensure that you have an ORCID iD and that it is validated in Editorial Manager.

**Reply 9:** We appreciate your suggestion to ensure to have a validated ORCID iD. Of course, my ORCID iD is <https://orcid.org/0000-0001-6347-0163>. I checked it out and that is validated in Editorial Manager.

**Reviewers'comments:

Reviewer#1**: Dear respected authors,
The authors of this manuscript present the results of about two years precise work to launch a prospective cohort study in southern Iran. The efforts of these colleagues and their supporter team in the Ministry of Health are commendable.

Please note the following:
**Comment 1**: I suggest that the manuscript should be read by a native English language editor to check and correct the grammatical mistakes. For example, in line 27, “in” was repeated twice, or at the beginning of many sentences, digits were seen, which must be written in letters, and ...

**Reply 1:** We welcome your suggestion. With respect to your valuable comment, we corrected the grammatical errors throughout the text. As much as possible, we did our best to shorten the text given that the concept and the essence of the study under the defined objectives remain intact. Based on the principles of good practice for academic writing, all the authors with aid of English native colleagues who are scientist and expert in the field and academic writing, read the manuscript thoroughly and meticulously for several times.

**Comment 2:** According to the 2016 census in I R of Iran, the M/F ratio in Hormozgan province was 51% to 49%, in comparison the ratio in this cohort study, where the ratio of men to women is 42.5% to 57.5%. There is a big difference. The study population may not represent the main population in the province.

**Reply 2:** We appreciate your meticulous comment. As we stated in the subheading “Strengths and Limitations”, the sample size is not that much large for a population-based prospective cohort; that is why, perhaps, this weakness being the argument to the observed difference. To tackle the issue, we started increasing the sample size twofold to normalize the male to female ratio as the main population of the province. Second, we realize that the province possesses a different cultural behavior and the major of the men are in sea-related industry such as fishing, ship making and etc. whereas most of the women are housewives. Therefore, the ratio of women participated in the study isn’t the same as the sex ratio of the province. Most probably, One of the main reasons is men’s employment and their limited time for participation.

**Comment 3:** In general, the manuscript is too long. It is better to reduce words to about 3000 to 3500.

**Reply 3:** We agree with your comment. We also like to keep the manuscript as short as possible, however, in this particular study, we have to maintain the concept and essence of the study according to the pre-defined objectives. Explaining the importance of each section was expected to give a look of lengthy text especially in the Method and materials. However, we revised/modified and tried our best to shorten it much more. Kindly note we have now reshaped and shortened the manuscript largely by deleting text where most appropriate as per the reviewers’ valuable suggestions. We really hope that you will be considerate to it and our explanations satisfy you.

**Comment 4:** Comparison of significance level between men and women in cohort profile manuscripts is usually not required.

**Reply 4:** This has been done like some other studies listed below (1,2). However, if you think it should be removed to improve the manuscript, we will delete it.

1. Pasdar Y, Najafi F, Moradinazar M, Shakiba E, Karim H, Hamzeh B, Nelson M, Dobson A. Cohort profile: Ravansar Non-Communicable Disease cohort study: the first cohort study in a Kurdish population. International journal of epidemiology. 2019 Jun 1;48(3):682-3f.
2. Mirzaei M, Salehi-Abargouei A, Mirzaei M, Mohsenpour MA. Cohort Profile: The Yazd Health Study (YaHS): a population-based study of adults aged 20–70 years (study design and baseline population data). International journal of epidemiology. 2018 Jun 1;47(3):697-8h.

**Comment 5:** It is written in line 152: At the beginning, and scientific committee…. Which does not seem to be correct.

**Reply 5:** Thanks for your comment. We corrected that.

**Comment 6:** What were the selection criteria of 4200 participants out of about 6000 people at the beginning of the study?

**Reply 6:** We have clarified that in the text as follows:

“At first, the scientific committee enrolled 4200 participants out of 6000 permanent residents aged 35-70 years subjects in accordance with PERSIAN Cohort agreement. The inclusion criteria were: (i) willingness to participation, (ii) aged 35-75, (iii) residency for one year and at least nine months each year, (iv) written informed consent, (v) Iranian national. Exclusion criteria included: (i) no interest to attend, (ii) being guest, and (iii) mental or physical disabilities.”

**Comment 7:** Are the demographic differences between those who participated in the study and those who did not, compared?

**Reply 7**: It has not done. We have no meticulous and detailed information about the people who didn’t participate in the study.

**Comment 8:** Line 234, what diagnostic code is used in the study.

**Reply 8**: As we stated in text, two external internists independently review all available clinical documents and allocate a disease code and a date of occurrence to each outcome. The two disease codes are compared, and if they are different, a third, more senior internist reviews the data and makes the final decision on the code. The “code” was assigned based on ICD10 (international classification of diseases).

**Comment 9:** In the statistical analysis section, several tests are mentioned, but there is no report of some of them in the result section. Of course, there is no need for statistical analysis in the cohort profile manuscripts.

**Reply 9**: We appreciate your comment and agree with that. Hence, we removed the unnecessary tests in this phase of the cohort study. Therefore, we modified statistical analysis as per your suggestion.

**Reviewer #2**: this research is unique in south of Iran but in case of statistical analysis, an expert scientist should evaluate the results, however, there are several cohort studies in Iran. I think it is worth nothing that this manuscript describes these types of diseases in specific part of Iran and compared with another region of Iran.

**Reply:** Thanks for your comments.

First, we described in the text under the subheading of “Method and materials” that a panel of experts and academics (scientists) were engaged in the design and conducting the study. Furthermore, we included one epidemiologist and one biostatistician in the scientific committee so as they used to evaluate the results and monitor the progress of the ongoing cohort in a regular basis.

Second, as per your suggestion, we compared some of the important demographic and clinical characteristics at base line among current running various cohorts at national level, in the Discussion section.

Upon acceptance of the manuscript, the raw data will be made available at this site and the access would be free for all academic users under a click wrap protection.

We thank the reviewers and editorial team for helping us improve our manuscript.

We are highly hopeful that the modifications in the manuscript and our explanations satisfy the referees. Kindly note we have now reshaped the manuscript by adding and deleting text where most appropriate as per the reviewers’ valuable suggestions.

However, we are afraid now with so many of your suggestions that have been incorporated in the text, it has shortened largely, although we revised and tried our best to shorten it much more. We really hope that you will be considerate to it.

We are not averse to further modifications of the language or the text.

We are highly hopeful that the ms finds potential, could fulfill the high standards of the journal and gets positive review.

Warm regards,

Azim Nejatizadeh, MD., PhD.

Corresponding author

Hormozgan University of Medical Sciences,

Bandar Abbas, Iran

Mobile: +98-9179564291

E-mail: azimnejate@yahoo.com
