# Supplementary material for: Cohort profile: Bandar Kong prospective study of chronic non-communicable diseases
Source: PLoS One. 2022 May 12;17(5):e0265388. doi: 10.1371/journal.pone.0265388 (PMC9098057; doi:10.1371/journal.pone.0265388)
Supplement: Attachment — Submitted filename: Response to Reviewers-18 June 2021.docx [file pone.0265388.s006.docx]

The Editor-in-chief

*PLOS ONE*

With respect,

Responses to the referee's comments:

Manuscript Number: PONE-D-20-32677R1

Title: Reply to Manuscript Number: PONE-D-20-32677R1

Title: Cohort Profile: The Bandar Kong Prospective Cohort Study

Article Type: Original research paper (Cohort profile)

Thanks a lot for your critical and peer review of our manuscript (ms). Our manuscript has been accepted with revision. We have provided a point-by-point response to all comments from the reviewer and have revised the manuscript accordingly. Therefore, we resubmit our manuscript (ms) titled “**Cohort Profile: The Bandar Kong Prospective Cohort Study**”, authored by **Azim Nejatizadeh, *et al***. Efforts have been made to keep the text to minimum with inclusion of the most relevant references. We edited the text and made the corrections and needful modifications as per the reviewer's comments. Furthermore, we have incorporated the editorial corrections in the revised version.

**Academic Editor**
One of the reviewers still has major problem with the statistical analysis; Please revisit his concern as follow:

For example, without using OR, it is mentioned again and sample size selection eligibility criteria. And the repeated-measurement phase. Also addressing the issue of external validity. How many strategies was used to control of many kind of bias in the study.

**Reply:** I would like to thank you again for raising and reflecting the reviewer's concern.

1. At first, regarding OR, I explained and added more findings by performing multivariable analysis represented as "**Adjusted OR (95%CI) and P-value"** shown in **Table 8** on some of factors affecting hypertension, diabetes, and heart diseases in the present study. We embedded the Table 8 within the manuscript followed by interpretation of the generated results as follow:

**Determinates of some of factors affecting hypertension, diabetes, and cardiac vascular disease:** Association between the increase of risk of hypertension and age, being married, being a widow/divorced, and living in a rural area was found by adjusted ORs (Table 8). Based on table2, the aged 55-64 people were more likely to be at risk of hypertension compared to the aged 35-44 people (AOR=1.30, 95% CI: 1.07,1.57). The odds to be at risk of hypertension was 1.81 and 2.93 times higher for married and widowed/divorced people than the single ones, respectively (AOR =1.81, 95% CI: 1.06,3.09; AOR =2.93, 95% CI: 1.64,5.22). Also, living in a rural area increased the odds of having hypertension by 1.33 (AOR=1.33,95% CI: 1.09,1.61).

Moreover, association between the increase of risk of diabetes and being female, being married, being a widow/divorced, and living in a rural area was found. By adjusting the effect of other variables, being female was related to a higher risk of diabetes (AOR=1.38, 95% CI: 1.16,1.64). The odds to be at risk of diabetes was 2.02 and 2.59 times higher for married and widowed/divorced people than the single ones, respectively (AOR =2.02, 95% CI: 1.04,3.94; AOR =2.93, 95% CI: 1.27,5.28). Also, living in a rural area increased the odds of having hypertension by 1.33 (AOR=1.33,95% CI: 1.09,1.61).

In addition, age, being a widow/divorced and BMI increased the odds of having cardiac vascular disease. By controlling the effect of other variables, the aged 55-64 people were more likely to be at risk of cardiac vascular disease compared to the aged 35-44 people (AOR=1.58, 95% CI: 1.17,2.13). Also, the odds of having cardiac vascular disease was 4.42 times higher for widowed/divorced people than the single ones (AOR=4.42, 95% CI: 1.33,14.72). Furthermore, the odds of having the cardiac vascular disease was higher for people who had BMI≥25 than people who had 18.5≤BMI≤ 24.9.

2. Regarding "sample size selection eligibility", within the text under the subtitle " Study population" we explained that the scientific committee enrolled 4200 participants in accordance with PERSIAN Cohort agreement. Should the prevalence of the outcome in the exposed group is 20%, 270 cases of interest would offer 90% power to detect a relative risk of 2. To obtain 270 cases over a period of 5 years, only 54 cases of interest per year is needed. The inclusion criteria were: (i) willingness to participation, (ii) aged 35-75, (iii) residency for one year and at least nine months each year, (iv) written informed consent, (v) Iranian national. Exclusion criteria included: (i) no interest to attend, (ii) being guest, and (iii) mental or physical disabilities**.** Furthermore, we started increasing the sample size twofold to normalize the male to female ratio as the main population of the province.
3. "Regarding repeated measurements" it is worth clarifying that analysis can be used to assess changes over time in an outcome measured serially or to test for differences in 1 or more treatments based on repeated assessments in the same subjects. Hence, we planned and initiated to follow up the participants each year by phone to look for: a) to renew the phone calls and addresses, b) reexamination of past medical history, c) investigating any recent treatment, hospitalization, NCDs or death. Therefore, the whole recruitment phases and repeated measurements every 5-year for 15 years with annual follow-up according to the standard PERSIAN Cohort protocol. We, now, have completed the third annual follow-up and just started the second 5-year recruitment phase and repeated measurements.
4. …. Also addressing the issue of external validity. How many strategies was used to control of many kind of bias in the study.

In this study, in order to improve the external validity and to increase the generalizability of the study results to the target community, we selected eligible individuals from both sex groups with diverse education and jobs as well as unlike socio-economic status to represent that the designated individuals to be the appropriate representative from the reference population. To control and monitor carefully the quality and accuracy of measuring devices such as scales and blood pressure, we calibrated all the concerned devices periodically according to the standard protocol. Furthermore, training and certifying the observers' skills and standardizing the measurement methods were the two other strategies to control information bias that we used to regularly carry out and practice these approaches in our ongoing cohort study.

To the best of my knowledge, one practical method to identify confounders is to analyze data with or without potential confounders. If the estimation of the association among the independent variables and the outcomes varies considerably by controlling a third variable, then the third variable would be confounding. Therefore, in the present study, using statistical methods such as regression models, the relationship between the independent variable and the desired outcome in different categories of confounding variables is calculated separately or controlled by statistical methods such as analysis stratification. We really hope that you will be considerate to it and our explanations satisfy the Reviewer.

**Review Comments to the Author**

**Reviewer#1:** Dear Authors,
Thank you for paying attention to my comments. Some of my comments have not been corrected. Including in the statistical analysis section. For example, without using OR, it is mentioned again. In general, in my opinion, it is better to first try to publish this study as a protocol of the cohort, and then after obtaining more results and publishing articles from the study results, proceed to publish the profile. For instance, after the repeated-measurement phase.
Strengths and weaknesses: I would recommend addressing the issue of external validity. How many strategies was used to control of many kinds of bias in the study.

**Reply to Reviewer#1 comments:**

At first, regarding "OR", we explained and added more findings by performing multivariable analysis represented as "**Adjusted OR (95%CI) and P-value"** shown in **Table 8** on some of factors affecting hypertension, diabetes, and heart diseases in the present study. We embedded Table 8 within the manuscript followed by interpretation of the generated results as follow:

**Determinates of some of factors affecting hypertension, diabetes, and cardiac vascular disease:** Association between the increase of risk of hypertension and age, being married, being a widow/divorced, and living in a rural area was found by adjusted ORs (Table 8). Based on table2, the aged 55-64 people were more likely to be at risk of hypertension compared to the aged 35-44 people (AOR=1.30, 95% CI: 1.07,1.57). The odds to be at risk of hypertension was 1.81 and 2.93 times higher for married and widowed/divorced people than the single ones, respectively (AOR =1.81, 95% CI: 1.06,3.09; AOR =2.93, 95% CI: 1.64,5.22). Also, living in a rural area increased the odds of having hypertension by 1.33 (AOR=1.33,95% CI: 1.09,1.61).

Moreover, association between the increase of risk of diabetes and being female, being married, being a widow/divorced, and living in a rural area was found. By adjusting the effect of other variables, being female was related to a higher risk of diabetes (AOR=1.38, 95% CI: 1.16,1.64). The odds to be at risk of diabetes was 2.02 and 2.59 times higher for married and widowed/divorced people than the single ones, respectively (AOR =2.02, 95% CI: 1.04,3.94; AOR =2.93, 95% CI: 1.27,5.28). Also, living in a rural area increased the odds of having hypertension by 1.33 (AOR=1.33,95% CI: 1.09,1.61).

In addition, age, being a widow/divorced and BMI increased the odds of having cardiac vascular disease. By controlling the effect of other variables, the aged 55-64 people were more likely to be at risk of cardiac vascular disease compared to the aged 35-44 people (AOR=1.58, 95% CI: 1.17,2.13). Also, the odds of having cardiac vascular disease was 4.42 times higher for widowed/divorced people than the single ones (AOR=4.42, 95% CI: 1.33,14.72). Furthermore, the odds of having the cardiac vascular disease was higher for people who had BMI≥25 than people who had 18.5≤BMI≤ 24.9.

We also added the following more findings to the table 6.

| **Domain** | **Variable** | **Men** | **Women** | **P-value** |
| --- | --- | --- | --- | --- |
| Self-reported  disease | Fracture | 106(2.6%) | 141(3.5%) | 0.947 |
|  | Osteoporosis | 20(0.5%) | 262(6.5) | <0.001 |

We appreciate the reviewer's comments, however, we do believe that the respected reviewer agrees with us that at present, the ongoing study owns enough findings and has generated adequate data, so as, it has merit and could get published as a "**Cohort profile**". We really hope that you will be considerate to it and our explanations satisfy you.

In this study, in order to improve the external validity and to increase the generalizability of the study results to the target community, we selected eligible individuals from both sex groups with diverse education and jobs as well as unlike socio-economic status to represent that the designated individuals to be the appropriate representative from the reference population.

To control and monitor carefully the quality and accuracy of measuring devices such as scales and blood pressure, we calibrated all the concerned devices periodically according to the standard protocol. Furthermore, training and certifying the observers' skills and standardizing the measurement methods were the two other strategies to control information bias that we used to regularly carry out and practice these approaches in our ongoing cohort study.

To the best of my knowledge, one practical method to identify confounders is to analyze data with or without potential confounders. If the estimation of the association among the independent variables and the outcomes varies considerably by controlling a third variable, then the third variable would be confounding.

Therefore, in the present study, using statistical methods such as regression models, the relationship between the independent variable and the desired outcome in different categories of confounding variables was calculated separately or controlled by statistical methods such as analysis stratification.

We thank the reviewers and editorial team for helping us improve our manuscript.

We are highly hopeful that the modifications in the manuscript and our explanations satisfy the reviewer. Kindly note we have now reshaped the manuscript by adding and deleting text where most appropriate as per the reviewer's suggestions.

We really hope that you will be considerate to it.

We are not averse to further modifications of the language or the text.

We are highly hopeful that the ms finds potential, could fulfill the high standards of the journal and gets positive review as well as acceptance.

Warm regards,

Azim Nejatizadeh, MD., PhD.

Corresponding author

Hormozgan University of Medical Sciences,

Bandar Abbas, Iran

Mobile: +98-9179564291

E-mail: azimnejate@yahoo.com
