# Supplementary material for: Cohort profile: Bandar Kong prospective study of chronic non-communicable diseases
Source: PLoS One. 2022 May 12;17(5):e0265388. doi: 10.1371/journal.pone.0265388 (PMC9098057; doi:10.1371/journal.pone.0265388)
Supplement: Attachment — Submitted filename: Response to Reviewers.docx [file pone.0265388.s007.docx]

The Editor-in-chief

*PLOS ONE*

With respect,

Responses to the referee's comments:

Manuscript Number: PONE-D-20-32677

Title: Reply to Manuscript Number: PONE-D-20-32677Title: Cohort Profile: The Bandar Kong Prospective Cohort Study

Article Type: Original research paper (Cohort profile)

Thanks a lot for your critical and peer review of our manuscript (ms). Our manuscript has been accepted with revision. We have provided a point-by-point response to all comments from the reviewers and have revised the manuscript accordingly.

Therefore, we resubmit our manuscript (ms) titled “**Cohort Profile**: **The Bandar Kong Prospective Cohort Study**”, authored by Azim Nejatizadeh *et al*. Efforts have been made to keep the text to minimum with inclusion of the most relevant references. We edited the text and made the corrections and needful modifications as per the reviewer comments. Notably, we have incorporated the all raised corrections in the revised version.

**Answers to each point raised by the academic editor and reviewer(s)**

**Journal Requirements:**

Please review your reference list to ensure that it is complete and correct. If you have cited papers that have been retracted, please include the rationale for doing so in the manuscript text, or remove these references and replace them with relevant current references. Any changes to the reference list should be mentioned in the rebuttal letter that accompanies your revised manuscript. If you need to cite a retracted article, indicate the article’s retracted status in the References list and also include a citation and full reference for the retraction notice.

**Responses to the** **Journal Requirements:**

Thanks for the reminder. We went through the reference list and found it comprehensive, however, reference No. 30 was in duplicate the same as reference No. 11. We deleted reference No.30 and kept reference No.11 in the list. The rest of the reference list was accurate and none of the cited papers were retracted.

Additional Editor Comments (if provided):

Please see the statistical revision that the reviewer requested you to revisit. This is the last time we can consider the manuscript to be re-reviewed. Please make sure you respond to the minor revision that the reviewer asks for.

**Reply:**

We have provided a point-by-point response to all comments from the reviewers and have revised the manuscript accordingly. We edited the text and made the corrections and needful modifications as per the reviewer comments. Notably, we have incorporated the all raised corrections in the revised version.

**Reviewers'comments:**

**Reviewer #3**:

**The statistical analysis is acceptable. However**,

**Comment 1**: I have some concerns about sample size calculations, I think the collected sample size is too small and setting and OR of 2 or more is rather big difference to detect. I need to see robust evidence that the calculated sample size over 5 years is supported by statistical methods, even for detecting a change in OR of 2.

**Reply 1:** About the appropriate sample size, the required sample size was calculated by Fleiss with continuity correction formula, with a two-sided significance level(1-alpha) = 0.95, power (1-beta, % chance of detecting) = 85%, the ratio of sample size of unexposed/exposed = 1, percent of unexposed with outcome = 5%, and risk/ prevalence ratio or odds ratio =1.5. So, it was estimated 2017 people for the exposed group, 2017 non-exposed group, and hence total sample size is 4034 people [Reference]. Accordingly, we have now added the above-mentioned explanations within the "**Biostatistics Analysis section**" as per the reviewers’ valuable suggestion.

Reference:

Dean AG, Sullivan KM, Soe MM. OpenEpi: Open Source Epidemiologic Statistics for Public Health, Version. www.OpenEpi.com, updated 2013/04/ 06, accessed 2017/05/06.

**Comment 2**: Data collection section is not clearly explained, the population that the data was collected from seems to be different from "'normal" population. Authors claimed that ''70% of the participants were affected by at least one NCD.'' this cannot be a normal population.

**Reply 2:** Thanks for your comment. We did our best to keep the manuscript as short as possible, however, in this particular study, we have to maintain the concept and essence of the study according to the objectives. However, according to your valuable suggestion, we now explained in detail and clearly "**Data collection section**" as well as **"Enrollment phase sections"**. Of note, we revisit and checked out the data analysis carefully and found that you are right. The claim ''70% of the participants were affected by at least one NCD", was a rough incorrect estimation and expressed by mistake; hence, we apologize for that. Accordingly, we have now deleted that sentence and revised/modified the "**Findings to date and Abstract**" sections where most appropriate.

Subsequently, the prevalence of self-reported health conditions of study participants and the most common chronic NCDs diagnosed during the first annual follow-up were listed in Tables 6 and 7 in detail. Thereby, these tabulated data, here, are our reference and quite valid. We really hope that you will be considerate to it and our explanations satisfy you.

**Comment 3**: P-values reported without explaining what they are testing, for example ''Over 23% of men and 17.3% of women were Hookah smoker (p-value <0.001)'' what is this p-value?

**Reply 3:** Thanks for your valuable comment. Below the tables, it was explained which tests were used, as following:

**^1^** The independent samples t-test was used to determine if there is a significant difference between the means of two groups, the chi-squared test was used to determine whether there is an association between 2 categorical variables.

**Comment 4**: Tables are confusing, not enough or missing explanation in the title or body of the tables; for example, table 4 and 6 no units are given for variables. Are they percentage or mean or mixed?

**Reply 4:** Thanks for your valuable comment; you are correct. The title of the tables was modified and units were added within the Tables. Where appropriate, missed percentage symbols were incorporated within the Table 6.

**Regarding conclusion section:**

We, again, did our best to draw and support appropriate conclusion based on the findings to date. Now, we included more data and inferred proper conclusions that have been argued in conclusion section. Here, we stressed that prevention of premature deaths due to NCD and decrease of associated health care expenses will be the main objectives of health policy. The consequences of such investigations would aid policy makers to plan and implement profitable facility within the situation of primary health care in Iran, serving as an instance for neighboring countries too.

We also like to keep the manuscript as short as possible, however, in this particular study, we have to maintain the concept and essence of the study. Kindly note we have now revised/modified and reshaped the "**conclusion as well as strengths and limitations**" sections where most appropriate as per the reviewers’ valuable suggestions. We really hope that you will be considerate to it and our explanations satisfy you.

Upon acceptance of the manuscript, the raw data will be made available at this site and the access would be free for all academic users under a click wrap protection.

We thank the reviewers and editorial team for helping us improve our manuscript.

We are highly hopeful that the modifications in the manuscript and our explanations satisfy the referees. Kindly note we have now reshaped the manuscript by adding and deleting text where most appropriate as per the reviewers’ valuable suggestions.

However, we are afraid now with so many of your suggestions that have been incorporated in the text, it has shortened largely, although we revised and tried our best to shorten it much more. We really hope that you will be considerate to it.

We are not averse to further modifications of the language or the text.

We are highly hopeful that the ms finds potential, could fulfill the high standards of the journal and gets positive review.

Warm regards,

Azim Nejatizadeh, MD., PhD.

Corresponding author

Hormozgan University of Medical Sciences,

Bandar Abbas, Iran

Mobile: +98-9179564291

E-mail: azimnejate@yahoo.com
