# Supplementary material for: Cohort profile: Bandar Kong prospective study of chronic non-communicable diseases
Source: PLoS One. 2022 May 12;17(5):e0265388. doi: 10.1371/journal.pone.0265388 (PMC9098057; doi:10.1371/journal.pone.0265388)
Supplement: Attachment — Submitted filename: Response to reviewers-final.docx [file pone.0265388.s008.docx]

The Editor-in-chief

*PLOS ONE*

With respect,

Responses to the referee's comments:

Manuscript Number: PONE-D-20-32677R3

Title: Reply to Manuscript Number: PONE-D-20-32677R3

Title: Cohort Profile: **Cohort profile:** [**Bandar Kong Prospective study of Chronic Non-Communicable Diseases**](https://bmcpublichealth.biomedcentral.com/articles/10.1186/1471-2458-12-803)

Article Type: Original research paper (Cohort profile)

Thanks a lot for your critical and peer review of our manuscript (ms). Our manuscript has been accepted with minor revision. We have provided a point-by-point response to all comments from the reviewers and have revised the manuscript accordingly.

Therefore, we resubmit our manuscript (ms) titled “**Cohort profile:** [**Bandar Kong Prospective study of Chronic Non-Communicable Diseases**](https://bmcpublichealth.biomedcentral.com/articles/10.1186/1471-2458-12-803) ”, authored by Azim Nejatizadeh *et al*. Efforts have been made to keep the text to minimum with inclusion of the most relevant references. We edited the text and made the corrections and needful modifications as per the reviewer comments. Notably, we have incorporated the all raised corrections in the revised version.

**Answers to each point raised by the academic editor and reviewer(s)**

**Journal Requirements:**

Please review your reference list to ensure that it is complete and correct. If you have cited papers that have been retracted, please include the rationale for doing so in the manuscript text, or remove these references and replace them with relevant current references. Any changes to the reference list should be mentioned in the rebuttal letter that accompanies your revised manuscript. If you need to cite a retracted article, indicate the article’s retracted status in the References list and also include a citation and full reference for the retraction notice.

**Responses to the** **Journal Requirements:**

Thanks for the reminder. We have not cited papers that have been retracted. while undertaking corrections and modification in the text, to improve the quality of the evidence of the manuscript, we decided to include eight new references to the reference list which have been embedded within the text. These are references No. 2, 5, 27, 33-35, 37, 38.

**Review Comments to the Author**

**Reviewer #3:**

**Comment 1**: The manuscript still needs some corrections and editing.

**Reply 1:** Thanks for your valuable comment; you are correct. We are highly hopeful that the modifications in the manuscript and our explanations satisfy you. Kindly note we have now reshaped the manuscript by adding and deleting text where most appropriate as per your valuable suggestions. We edited and corrected the text thoroughly, however, in this particular study, we have to maintain the concept and essence of the study.

**Comment 2**: The sample size explained to my previous comment looks OK but it is very different from what is explained under "Study Population" in line 136.

**Reply 2:** Thanks for your valuable comment. It is modified. The modifications were highlighted in green from line 229 to 233.

**Comment 3**: Some tables are out of format (table 4 and table 8) and unreadable.

**Reply 3:** Thanks for your comment. You are correct. We corrected format of the tables 4 and 8 and now are readable. Remaining tables also were organized appropriately.

**Comment 4**: The NCD should be somehow in the title of this study.

**Reply 4:** Thanks for your valuable comment. As per your suggestion, we incorporated NCD in the title of the manuscript. Now, it seems much better.

**Comment 5**: SPSS is not any more Statistical Package for Social Science, please correct it.

**Reply 5:** Thanks for your comment. We corrected and modified it.

**Comment 6**: line 246 to 249 is not technically correct, rewrite it

**Reply 6:** Thanks for your comment. We have revised and modified it.

**Comment 7**: You need to edit this manuscript carefully if you want to get it published in PLOS ONE.

**Reply 7:** Thanks for your comment. Now and again, we further did our best to edit the text carefully and keep the manuscript as short as possible, however, in this particular study, we have to maintain the concept and essence of the study according to the objectives. Therefore, we modified and edited the text wisely and whole manuscript thoroughly as per your suggestion. We really hope that you will be considerate to it where the edited text and our explanations satisfy you.

Upon acceptance of the manuscript, the raw data will be made available at this site and the access would be free for all academic users under a click wrap protection.

We thank the reviewers and editorial team for helping us improve our manuscript.

We are highly hopeful that the modifications in the manuscript and our explanations satisfy the referees. Kindly note we have now reshaped the manuscript by adding and deleting text where most appropriate as per the reviewers’ valuable suggestions. We really hope that you will be considerate to it.

We are not averse to further modifications of the language or the text.

We are highly hopeful that the ms finds potential, could fulfill the high standards of the journal and gets positive review.

Warm regards,

Azim Nejatizadeh, MD., PhD.

Corresponding author

Hormozgan University of Medical Sciences,

Bandar Abbas, Iran

Mobile: +98-9179564291

E-mail: azimnejate@yahoo.com
