# Supplementary material for: Correction: Cohort profile: Bandar Kong prospective study of chronic non-communicable diseases
Source: PLoS One. 2024 Dec 5;19(12):e0315430. doi: 10.1371/journal.pone.0315430 (PMC11620435; doi:10.1371/journal.pone.0315430)
Supplement: S1 File — (PDF) [file pone.0315430.s001.pdf]

PLOS ONE

Correction: Cohort profile: Bandar Kong prospective study of chronic non-communicable diseases

--Manuscript Draft--

**Manuscript Number:**	
**Article Type:**	Correction
**Corresponding Author:**	Nicholas Wrigley, M.F.A.<br>Public Library of Science<br>San Francisco, CA UNITED STATES OF AMERICA
**First Author:**	Nicholas Wrigley, M.F.A.
**Order of Authors:**	Nicholas Wrigley, M.F.A.

Powered by Editorial Manager® and ProduXion Manager® from Aries Systems Corporation
